# A New Eocene Casquehead Lizard (Reptilia, Corytophanidae) from North America

**DOI:** 10.1371/journal.pone.0127900

**Published:** 2015-07-01

**Authors:** Jack L. Conrad

**Affiliations:** 1 Department of Anatomy, NYIT College of Osteopathic Medicine, Northern Boulevard, Old Westbury, NY, 11568, United States of America; 2 Department of Vertebrate Paleontology, American Museum of Natural History, Central Park West at 79^th^ Street, New York, NY, 10024, United States of America; State Natural History Museum, GERMANY

## Abstract

A new fossil showing affinities with extant *Laemanctus* offers the first clear evidence for a casquehead lizard (Corytophanidae) from the Eocene of North America. Along with *Geiseltaliellus* from roughly coeval rocks in central Europe, the new find further documents the tropical fauna present during greenhouse conditions in the northern mid-latitudes approximately 50 million years ago (Ma). Modern Corytophanidae is a neotropical clade of iguanian lizards ranging from southern Mexico to northern South America.

## Introduction

Fossil members of various animal, plant, fungal, and other clades currently confined to the tropics or subtropical areas are often found in the mid-to-high latitudes during warm periods in Earth history [1, 2]. Future global climate change may disproportionately affect tropical species [3, 4], so it is important to understand previous biotic responses to climate perturbation [5]. When extended to understanding some infectious disease (e.g., malaria) distributions, this importance is magnified by medical implications.

The mean average global temperature was approximately 9°C higher in the mid-Eocene than today [6]. Fossils from these and similarly aged strata suggest a lush, closed-canopy, tropical environment with a diverse reptile fauna along the eastern flank of the ancestral Rocky Mountains [7–9].

**Fig 1:**
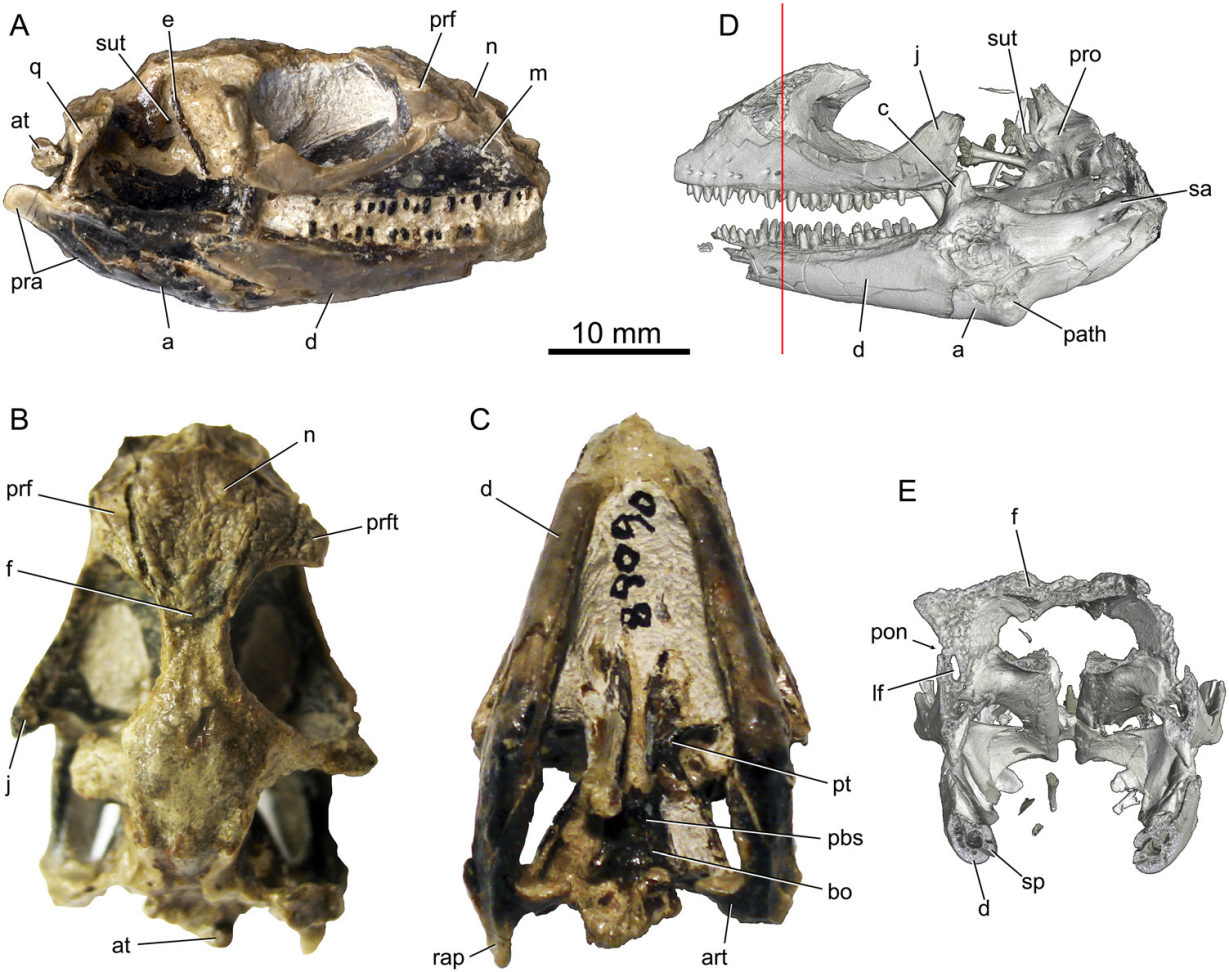
Holotype (UWBM 89090) specimen for *Babibasiliscus alxi* nov. taxon. Photographs in (A) right lateral, (B) dorsal, and (C) ventral views. Digital reconstructions derived from HRXCT in (D) left lateral view and (E) transverse section. The vertical red line in (D) indicates the plane of section in (E).

**Fig 2:**
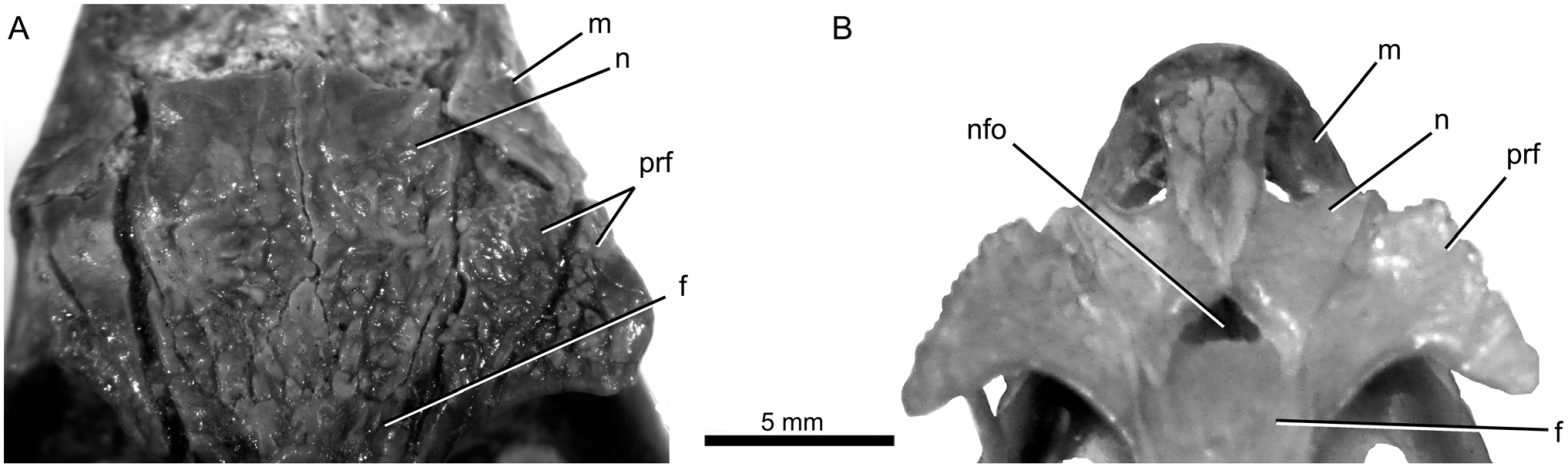
Comparison of the nasal and prefrontal regions of (A) *Babibasiliscus alxi* nov. taxon (UWBM 89090)and (B) *Corytophanes hernandezi* (AMNH R 147880) in dorsal view.

A new fossil corytophanid from the Bridger Formation (Fig 1) represents the sister-taxon to extant *Laemanctus* (Fig 2). Although modern corytophanids are restricted to Central and South America (Fig 2A and 2B), I posit that the group had a Euramerican/Laurasian origin and became restricted to lower latitudes after post-Eocene global cooling.

## Materials and Methods

### Institutional Abbreviations

AMNH, American Museum of Natural History (New York, NY); UWBM, Burke Museum of the University of Washington (Seattle, WA).

### Nomenclatural Acts

The electronic edition of this article conforms to the requirements of the amended International Code of Zoological Nomenclature, and hence the new names contained herein are available under that Code from the electronic edition of this article. This published work and the nomenclatural acts it contains have been registered in ZooBank, the online registration system for the ICZN. The ZooBank LSIDs (Life Science Identifiers) can be resolved and the associated information viewed through any standard web browser by appending the LSID to the prefix http://zoobank.org/. The LSID for this publication is: urn:lsid:zoobank.org:pub:F173E894-97D5-4C42-B760-BD7D7876656F. The electronic edition of this work was published in a journal with an ISSN, and has been archived and is available from the following digital repositories: PubMed Central and LOCKSS.

Clade names follow recent phylogenetic definitions [10, 11].

### Phylogenetics

The present phylogenetic analysis includes morphological data for 81 iguanomorph species and an anguimorph outgroup (Dataset A in S1 File). Characters and character states are largely from published analyses (Conrad et al. [12] and Gauthier et al. [13]), but with updates and modifications (Text B in S1 File). Supplementing the morphological characters are the molecular data included by Vieira et al. [14].

I used *NEXUS Data Editor* (*NDE*)[15] to assemble and manage the data matrix and performed an analysis using the New Technology Search in the computer program *T*.*N*.*T*: *Tree analysis using new technology*. One-thousand 1000 replicates were run with “ratchet” and “drift” options employed. I used PAUP* to reconstruct the Adams consensus.

### Anatomical Abbreviations

a, angular; aiac, anterior internal alveolar canal; art, articular tubercle; bo, basioccipital; at, atlas; chg, choanal groove; d, dentary; e, epipterygoid; f, frontal; ioc, infraorbital canal; j, jugal; L, left; l, lacrimal; lf, lacrimal foramen; m, maxilla; n, nasal; nfo, nasofrontal fontanelle; pa, palatine; path, pathological area; pbs, parabasisphenoid; pon, preorbital notch; pra, prearticular; prf, prefrontal; prft, prefrontal tubercle; pro, prootic; pt, pterygoid; q, quadrate; R, right; rap, retroarticular process; sa, surangular; sp, splenial; sut, supratrigeminal process.

## Results

### Systematic paleontology

Squamata Oppel [16]

Iguania Cuvier [17]

Corytophanidae

*Babibasiliscus* gen. nov.

urn:lsid:zoobank.org:act:CB390F92-FDDC-4CD8-B6A5-4E4C12836686

*Babibasiliscus alxi*, gen. et sp. nov.

urn:lsid:zoobank.org:act:F9FB7055-76FE-4BBB-8DD6-7B795B01BB41

Figs 1, 2A and 3–6.

#### Holotype

UWBM 89090; a nearly complete skull with lower jaws and parts of first two cervical vertebrae (Fig 1).

#### Etymology

*Babi*- (Shoshoni) meaning “older male cousin” and *Basiliscus*, a corytophanid lizard. The generic name is meant to honor the Shoshone people who originally inhabited the areas in which the specimen was discovered and to refer to the relationship of the lizard with corytophanid lizards. At the request of Christian A Sidor, the species name honors John P. Alexander, who discovered the holotype.

#### Locality and Age

Lucky Lizard Locality (UWBM C1046), Uinta County, Wyoming. Blacks Fork Member of Bridger Formation (Bridger B), Green River Basin, late Early Eocene, approximately 48 Ma.

#### Diagnosis

**Fig 3:**
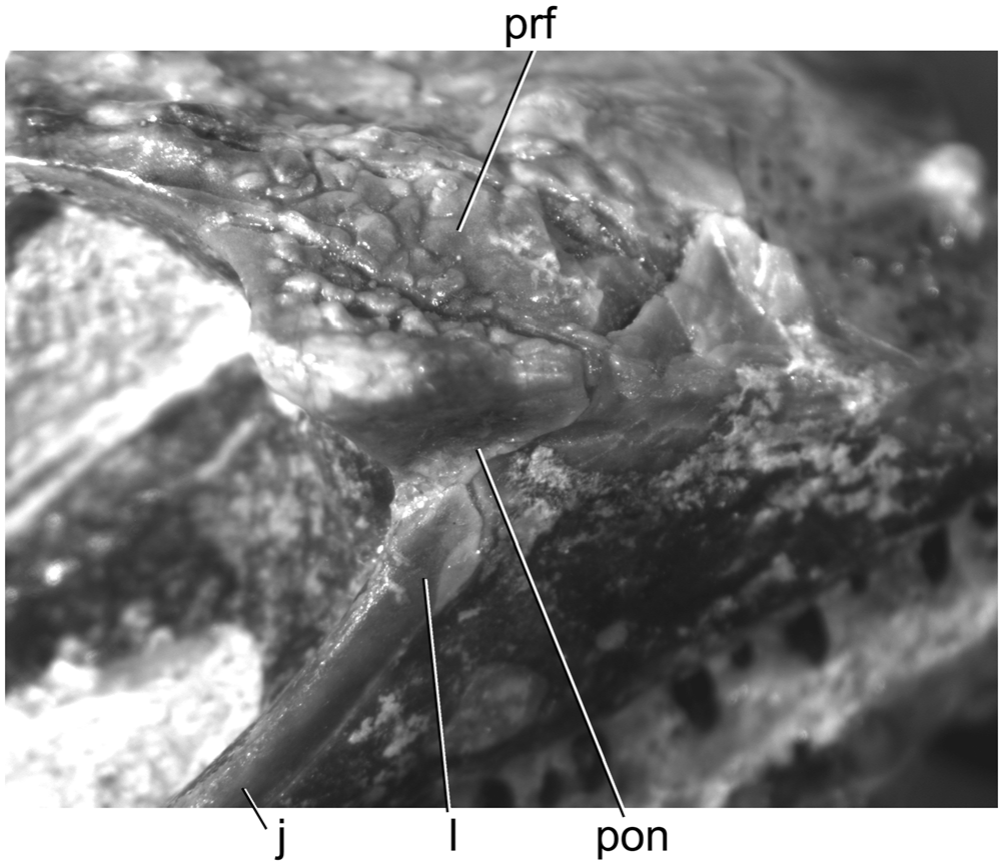
Right antorbital region of *Babibasiliscus alxi* nov. taxon (UWBM 89090) in dorsolateral view.

**Fig 4:**
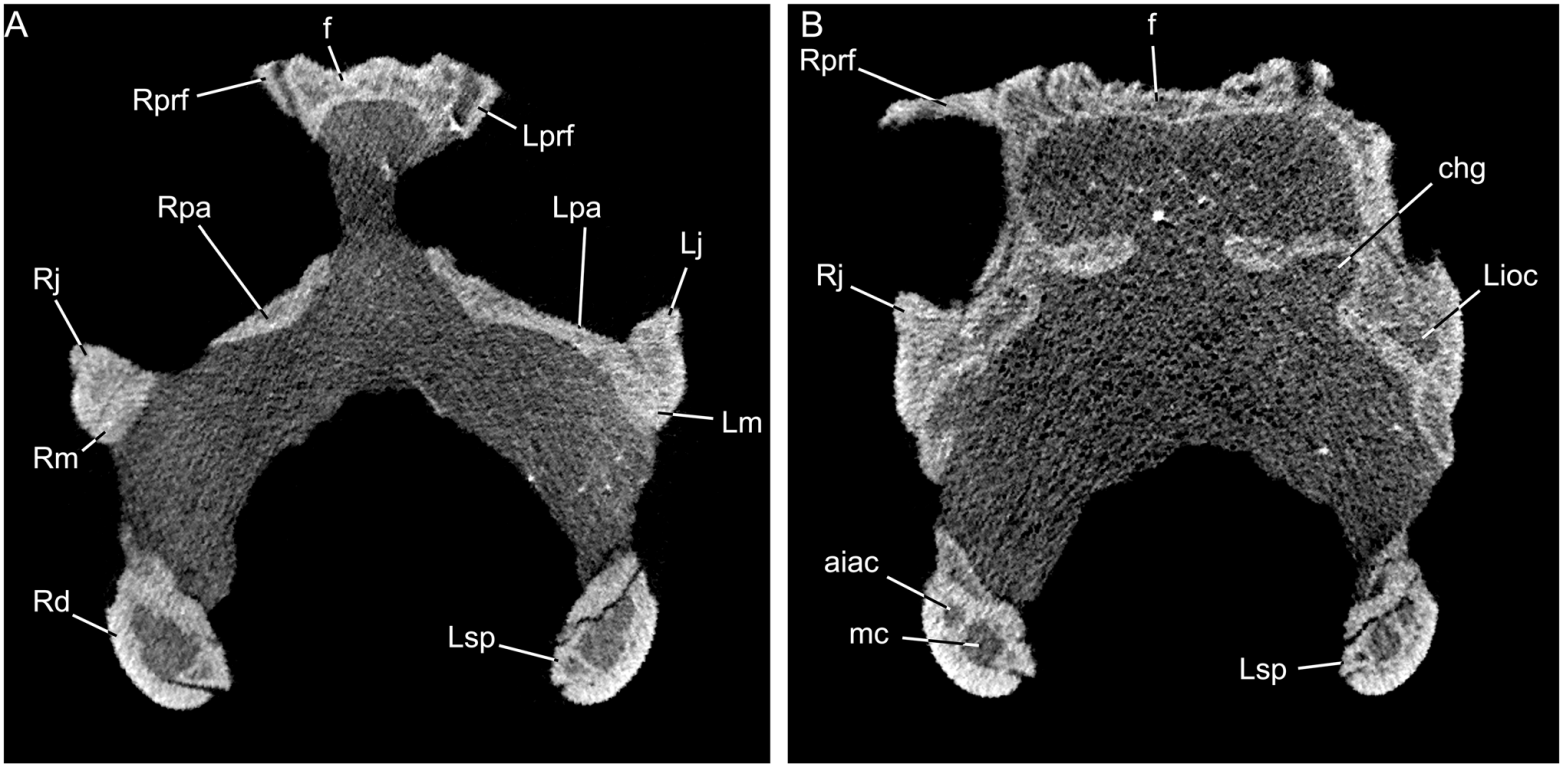
Transverse sections through the (A) orbital region and (B) preorbital region of *Babibasiliscus alxi* nov. taxon (UWBM 89090) illustrating details of bones and their contacts.

UWBM C1046 is a corytophanid iguanian possessing the following combination of character states: Smoothly curved posterior nasal margins (Figs 1B and 2B); prefrontal-lacrimal groove (Fig 3); absence of a defined entocarotid fossa; and absence of a retroarticular process pit. UWBM C1046 differs from *Geiseltaliellus* in possessing dermal sculpturing on the prefrontal (Figs 1A, 1B, 2A and 3). It differs from *Basiliscus* and *Laemanctus* in possessing an extensive internasal contact. It differs from *Laemanctus* in lacking posteriorly elongated osseous external nares, and in possessing a jugal that lies mostly dorsal to the maxilla; from *Corytophanes* and *Laemanctus* in possessing a well-developed septomaxilla, a reduced crista prootica, and a splenial that terminates anterior to the coronoid apex; from *Corytophanes* in lacking a nasofrontal fontanelle (also present in *Laemanctus lagnipes*), absence of a contact between the prefrontal and the osseous external naris, in possessing distinct supratrigeminal process of the prootic, and in possessing a ventromedial process of the pterygoid (also absent in some *Basiliscus*). It differs from *Basiliscus* in possessing a medially flared palatine flange of the maxilla (Fig 4) and anterolateral processes of the frontal that terminate at about the same anterior level as the midline frontal process (Figs 1B and 2A; rather than extending anterior to that level or terminating posterior to that level).

### Description and comparisons

#### Preservation

The specimen is an articulated, mostly complete skull with mandibles and the first two vertebrae preserved without evidence of plastic deformation (Fig 1). The premaxilla, posterior half of the frontal, parietal, postfrontals, postorbitals, squamosals, supratemporals, supraoccipital, dorsal half of each quadrate, and the anterior tips of both dentaries have been eroded away. The mandibles remain in articulation and are symmetrical in their alignment as preserved.

#### Cranial form

The preserved portion of the skull is approximately 42 mm from the anterior tip of the maxilla to the posterior margin of the occipital condyle. The complete skull would likely have been 2–3 mm longer.

**Fig 5:**
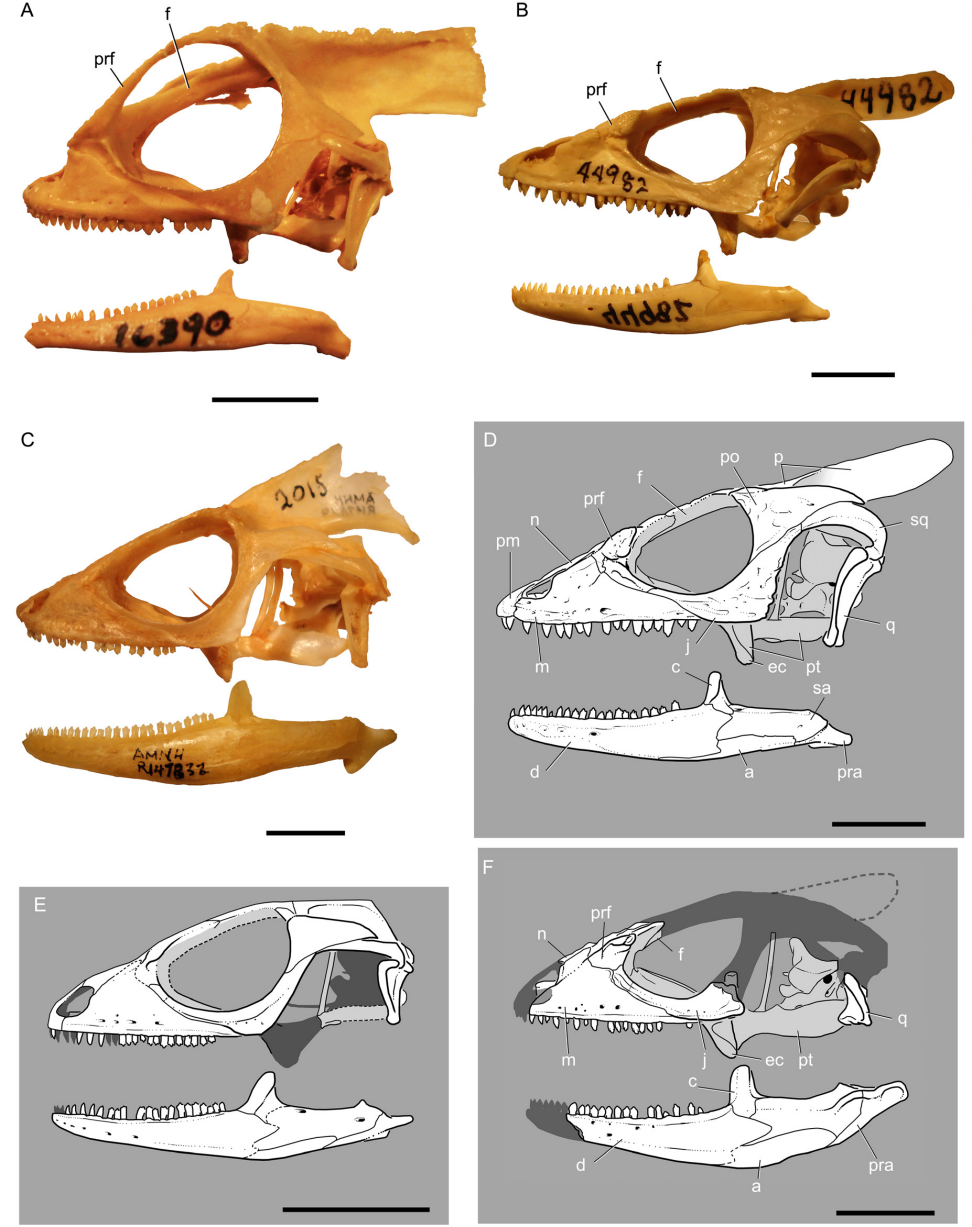
Photographs (A-C) and line drawings (D-F) of the skulls of selected corytophanid species in left lateral view. (A) *Corytophanes cristatus* (AMNH R 16390), (B) *Laemanctus serratus* (photograph; AMNH R 44982), (C) *Basiliscus vittatus* (AMNH R 147832), (D) *Laemanctus serratus* (line drawing), (E) *Geiseltaliellus maarius*, and (F) *Babibasiliscus alxi* taxon nov. (UWBM 89090). Note that it is unclear whether *Babibasiliscus alxi* taxon nov. had a parietal crest. Reconstructed areas are represented as semi-opaque areas and/or dotted lines. Scale bars equal 10mm.

Three-dimensional preservation of the nearly complete skull allows a confident reconstruction (Fig 5F), although the presence or absence of a parietal crest like that of extant corytophanids (Fig 5A–5D) is uncertain.

#### Maxilla

The apex of the nasal process on the unsculptured maxilla is located anterior to the midpoint of the tooth row. The maxilla does not contact the vomer posterior to the vomeronasal opening.

There are seven labial ethmoidal foramina on the left maxilla and five on the right. Two additional, more dorsally located, ethmoidal foramina are preserved on the left maxilla and one is present on the right. These are present in many iguanians, including some *Basiliscus* and *Laemanctus*, but were not observed in available *Corytophanes*. A small posterolateral apex of the maxilla partly invades the anterior part of the prefrontal-lacrimal suture. Posterorventrally, the dentigerous part of the maxilla broadly underlies the orbit and lies completely lateral to the ectopterygoid. Thus, there is no lateral exposure of the ectopterygoid on the external skull surface.

The maxilla narrowly contributes to the anterolateral margin of the suborbital fenestra between the ectopterygoid and palatine. The small palatine flange extends dorsomedially from the medial part of the maxilla.

#### Nasal

The highly sculptured nasals are incomplete anteriorly, but preserve their contacts with the maxillae, prefrontals, and frontal. They have slight dorsal bowing and their posterior margins are U-shaped in dorsal view. The nasofrontal suture is W-shaped and there is no nasofrontal fontanelle like that seen in some other pleurodontan iguanians (e.g., *Corytophanes hernandezi*, Fig 2B).

Each nasal preserves an anterolateral process that partly underlies the maxillary nasal process. This is similar to the condition in *Anolis carolinensis* [18] and *Laemanctus*, but not *Basiliscus* or *Corytophanes*.

#### Prefrontal

The left prefrontal is damaged somewhat anterolaterally, but the right is complete. The prefrontals bear strong dermal rugosities.

The right prefrontal demonstrates the presence of a distinct tuberosity that is expanded posterolaterally (Figs 1B and 2A). Although many iguanians possess a dorsolateral tuberosity, that of *Corytophanes*, *Basiliscus basiliscus*, and some phrynosomatids (e.g., *Phrynosoma asio*;) is posterolaterally developed such that it forms a partial or complete lateral supraorbital arch [14]. *Laemanctus* and *Babibasiliscus alxi* lack such a lateral supraorbital arch, but show a more strongly developed tuberosity than in most squamates. This may be an incipient morphology (or a vestige) of the condition seen in some other corytophanids. *Geiseltaliellus* does not show development of this morphology.

A prefrontal-lacrimal groove, a corytophanid synapomorphy (reversed in *Corytophanes*;) extends anteriorly from the orbital margin (Fig 3).

#### Lacrimal

The lateral margins of the lacrimals are obscured because of partial fusion with the jugal, but the margins are visible posteriorly. The lacrimal forms the anterolateral and lateral margins of the lacrimal foramen, the lateral part of the anterodorsal margin of the palatine foramen, and the ventral part of the antorbital margin.

#### Jugal

Both jugals preserve the suborbital processes, but more of the postorbital process is preserved on the right jugal. The maxilla-jugal contact is a butt-joint lacking strong the tongue-in-groove contact (Fig 4) seen in some squamates [13]. The base of the postorbital process is laterally flattened and broader than the suborbital part, with some development of anterior and posterior flanges.

#### Frontal

Approximately the anterior one-third of the azygous frontal is preserved and bears extensive dermal ornamentation. The W-shaped nature of the nasofrontal suture suggests the possibility of nasal shelf of the frontal as in some other iguanians, but HRXCT imagery demonstrates the absence of this shelf as in modern corytophanids (e.g., [19, 20]). The prefrontal narrowly overlies the anterolateral process of the frontal. The median, internasal, process of the frontal extends to approximately the same anterior level as the anterolateral processes (Figs 1B and 2A). In contrast to some extant pleurodontans, such as dactyloids, the frontal lacks dorsoventral inflation (Fig 4).

#### Parietal

Although the parietal is not preserved, impressions on the matrix that originally underlied that bone reveal a few details. The parietal fossa was located at about the level of the dorsal end of the epipterygoid. The postfoveal crests were well-developed and approached posteriorly, but it is unclear if they converged. However, lack of preservation of the parietal precludes comparison to the crested anatomy seen in extant corytophanids (Fig 5).

#### Vomer

The broad, plate-like vomers are preserved, but lack their anterior tips. A parasagittal crest extends from the most anteromesial preserved tip of the vomer to a point near the posteromedial vomeropalatine contact. This crest defines a shallow, dorsally open groove on the dorsal surface of the vomer. The vomerine canal passes posterodorsally through the vomer. The vomers contact along their entire length and vomerine teeth are absent.

#### Palatine

The elongate, toothless palatines form the anterior margin of the pyriform recess. There is no palatine-ectopterygoid contact. A short, but well-defined, choanal groove is present (Fig 4). A short palatine canal is present.

The anteromedially tapering vomerine process has a broad contact with the vomer. The maxillary process dorsally and ventrally overlaps the palatine flange of the maxilla. The dorsomedially oriented prefrontal process is distinct, but relatively short, not approaching the frontal. Thus, there is a broad exposure of the prefrontal in the orbitonasal fenestra.

The pterygoid process has a narrow ventrolateral ramus that partly clasps the pterygoid. Palatine teeth generally are absent in all observed extant corytophanids, but have been found in at least one specimen of *Laemanctus longipes*.

#### Pterygoid

Each ptergyoid contacts the palatine, ectopterygoid, parabasisphenoid, and quadrate. The anteriorly attenuated palatine process forms much of the lateral margin of the pyriform recess and largely underlies the posterior part of the palatine. As with modern corytophanids, the transverse process is short. Its main body is dorsoventrally broad and gently anterolaterally oriented. A thin horizontal crest forms a sort of horizontal bony webbing between the transverse process and the palatine process. At the confluence of the palatine, transverse, and quadrate processes, the main body of the pterygoid is cylindrical anteriorly and then dorsally excavated in a columellar fossa. The dorsoventrally deep quadrate process is medially concave.

The body of the pterygoid bears a strongly developed basisphenoid buttress. No pterygoid teeth are preserved, but there are shallow, ovoid depressions that would have been tooth attachment points just lateral to a weak longitudinal ridge. Thus, modified pleurodont attachment of pterygoid teeth (sensu [21]) was present.

#### Ectopterygoid

The mediolaterally-oriented ectopterygoid is expanded at both ends and constricted in the middle. The lateral facet is the shape of an acute triangle facing anteriorly; thus the facet is anteroposteriorly broad. The medial (pterygoid) facet is dorsoventrally broader than its anteroposterior breadth. It clasps the anterior surface of the transverse process of the pterygoid.

#### Quadrate

About half of each quadrate is preserved, including the condylar part. Pterygoid flanges are, apparently, absent (Fig 1). The tympanic crest is robust as in extant corytophanids and *Geiseltaliellus maarius*.

#### Epipterygoid

The right epipterygoid is preserved in articulation with the pterygoid and demonstrates that the epipterygoid is somewhat posterodorsally inclined. The epipterygoid is elongate (Figs 1A, 1D and 5), not foreshortened as in *Corytophanes* (Fig 5A).

#### Parabasisphenoid

The parabasisphenoid is well-preserved except for the parasphenoid rostrum. It possesses robust basipterygoid processes and well-developed posterolateral processes that ventrolaterally overlie the anterior part of the basioccipital. The cristae trabecularis are proximally joined with the parasphenoid rostrum.

The basipterygoid processes are set at about 80 degrees from one another. Their articular surfaces are only moderately expanded such that the basipterygoid processes do not appear to have the extreme neck present in some squamates (e.g., *Scincus scincus*). The length of the basipterygoid process is subequal to its distal width, contrasting the condition seen in extant corytophanids wherein the basipterygoid processes are usually somewhat longer than their distal breadths.

The pituitary fossa is indistinct. The anterior openings of the Vidian canals lie immediately lateral to the cristae trabecularis. The posterior opening of the Vidian canal lies posterior to the level of the trigeminal notch of the prootic, just anterior to the level of the facial foramen. There is no entocarotid fossa.

#### Basioccipital

The basioccipital is complete and well preserved (Fig 1C). Although there is no fenestra basicranialis, the basioccipital is very thin anteromedially. The spheno-occipital tubercles are robust and posteroventrally-oriented with broad proximal bases and narrow distal tips. The ventral part of the mediolaterally broad crista interfenestralis lies near the anterodorsal part of the spheno-occipital tubercle. The basioccipital-otooccipital and basioccipital-prootic sutures occur ventrally within the occipital recess. The basioccipital apparently has no contribution to the inner ear or even the medial aperture of the recessus scalae tympani (sensu [22]). The basioccipital contribution to the occipital condyle is wedge-shaped in cross-section and is slightly larger than either of the otooccipital contribution. Dorsomedially, the basioccipital part of the occipital condyle is gently concave and the dorsolateral otooccipital sutures are ventrolaterally oriented.

#### Prootic

Each prootic is damaged dorsally and lack the supraoccipital contacts (Fig 1D), but are otherwise preserved in contact with the surrounding bones. A short, dorsally-oriented alar crest is present on the dorsal surface of the bulbous auditory bulla. Such a crest is uncommon amongst iguanians, but is also present in *Basiliscus* and *Corytophanes*. The shallow trigeminal notch (sensu [23]; the incisura prooticum of some authors) is dorally bordered by the auditory bulla and ventrally by a broad, ventromedially-oriented, inferior process. The prootic paroccipital process is elongate. The prootic forms the anterior and anterodorsal margins of the fenestra ovalis.

The broad prootic crest (crista prootica) originates near the posteroventral border of the trigeminal notch and extends posterolaterally onto the prootic paroccipital process. It possesses lateral and descending ventrolateral processes. The inferior process extends anterior to the level of the auditory bulla and alar crest. An elongate supratrigeminal process overhangs the inferior process (Fig 1A and 1D).

#### Otooccipital

Both otooccipitals are damaged laterally and their preserved contacts are partly obscured by fusion. However, a remnant of the basioccipital suture demonstrates that the otooccipital forms most of the margins of the occipital recess, and the posterior and ventral margins of the fenestra ovalis. Distinct vagus and hypoglossal foramina are present posteriorly. The occipital condyle parts of the otooccipitals are separated at midline by the basioccipital. Lack of clear sutural contacts caused by bone inter-growth make the exact nature of the otooccipital contributions to the inner ear cavities unclear.

The lateral margin of the crista interfenestralis is straight and extends from a point just dorsal to the spheno-occipital tubercle to the base of the paroccipital process immediately posterior to the fenestra ovalis. This contrasts the condition observed in extant corytophanids wherein the lateral margin of the crista interfenestralis is slightly medially concave.

The perilymphatic foramen is located anterodorsally and somewhat superficially within the occipital recess. The medial aperture of the recessus scalae tympani occurs dorsally within the occipital recess at about the level of the extreme ventral convexity of the spheno-occipital tubercle.

#### Dentary

Both dentaries are damaged anteriorly (Fig 1A, 1C and 1D). The preserved portion constitutes approximately 60 percent of the preserved mandible. When complete, the dentary probably constituted about two-thirds of the total mandibular length. There is no coronoid process of the dentary.

The right dentary has been damaged posteriorly such that the nature of its contact with the postdentary bones is unclear. The left dentary is pathological posteriorly (see below). A short dental shelf is present dorsal to the splenial contact. Meckel’s canal is open for the entire preserved length of the dentary and extends along the medial surface of the dentary, approaching (but not extending onto) the ventral mandibular surface. This contrasts the condition in extant *Basiliscus* and *Corytophanes* where the dentary partly closes Meckel’s canal.

#### Splenial

Both splenials are preserved in articulation and lack only their anterior tips. They close Meckel’s canal for its entire preserved length. Their preserved portions demonstrate that they are more elongate than the splenials of extant corytophanids. The splenial completely houses the inferior alveolar foramen and the anterior mylohyoid foramen. Posteriorly, the splenial-dentary contact narrowly overlies the anterior tip of the coronoid.

#### Coronoid

Both coronoids are well preserved, although the left shows some deformation associated with a jaw pathology (Fig 1D). This pathology presents as an offset of the bones at the level of the coronoid with additional bone growth associated excess bone deposition. The coronoid forms the anterior margin of the adductor fossa (there is no exposed surangular-prearticular contact anterior to the adductor fossa). The coronoid is shaped like an inverted-V in lateral view, but also partly overrides the dorsal surface of the surangular at the level of the coronoid eminence.

The coronoid eminence is tall and anteroposteriorly narrow. Its dorsal margin is rounded. A broad labial flange is present, but does not extend far posteriorly.

#### Angular

Little can be determined regarding the morphology of the left angular (Fig 1D). The right angular is well preserved posteriorly, but somewhat eroded anteriorly. It spans approximately 40 percent of the mandibular height and tapers posteriorly. Anteriorly, it contributes a small part to the ventral margin of Meckel’s canal. The posterior mylohyoid foramen is present within the angular on the ventromedial surface of the mandible, posterior the anterior margin of the coronoid eminence.

#### Surangular

The well-preserved surangular is robust (Figs 1A, 1D, and 5F). It does not extend anteriorly to the level of the division between Meckel’s canal and the anterior alveolar canal. Posteriorly, there is a small buttress at the anterior margin of the mandibular glenoid. The surangular is narrowly exposed on the medial surface of the mandible between the anterior and posterior descending processes of the coronoid dorsally, and the angular ventrally. A posterolateral crest is well developed and extends laterally and dorsolaterally (Fig 5F).

The inferior alveolar canals passes anteriorly through the surangular from the posterior surangular foramen at the level of the glenoid fossa. The anterior surangular foramen is located on the dorsolateral surangular surface, just posterior to the coronoid apex. Its margins are formed exclusively by the surangular. The anterior surangular foramen and, therefore, the inferior alveolar canal connect with the posterior part of Meckel’s canal.

#### Prearticular and articular

The prearticular and articular form a fused unit hereafter referred to as the prearticular. It forms the ventral margin of the unexpanded mandibular fossa, the mandibular glenoid, and the retroarticular process. The mandibular glenoid is W-shaped in cross section with a weak mesial ridge and subequal medial and lateral facets. In dorsal view, the mandibular glenoid is rounded anteriorly with a straight posterior margin.

Most of the left retroarticular process is damaged and deformed, but the right is well preserved (Fig 1A and 1D). The retroarticular process is a little longer than the length of the mandibular glenoid. The posterior end of the retroarticular process is rounded and posteriorly directed with a slight posterodorsal incline. A robust, finger-like retroarticular process is present (Fig 1C).

#### Dentition

Eighteen tooth positions are evident on each dentary and between five and eight more tooth positions were likely present in a complete lower jaw. The teeth themselves are pleurodont with large resorption pits present at the tooth bases. The most anterior preserved teeth are conical. More posteriorly, the teeth develop weak shoulders. The last six tooth positions are weakly tricuspid with a strong central cusp and weakly developed anterior and posterior cusps. These cusps do not flare and there are no clear wear facets.

#### Mandibular pathology

The left mandible shows signs of injury and healing (Fig 1D). The posterior margin of the dentary shows extensive intergrowth with the more posterior mandibular elements. There is fusion with the angular. The coronoid is severely deformed. A large, bulbous mass of bone is present near the base of the coronoid. Posterior to the dentary and ventral to the coronoid, there are bone fragments that have been partly fused with the surangular dorsolaterally, perhaps representing a shattered posterior part of the dentary. Although the inferior alveolar canal appears to be intact and normal, the surangular foramina are enlarged and irregularly shaped. The angular shows excessive deposition of bone on the ventral mandibular surface such that the left angular is approximately six times the thickness of the right.

#### Atlas and Axis

The left atlantal arch, the atlas intercentrum, and the odontoid process of the axis are preserved. The atlas has a short and sharp lateral process and a short ventral keel.

### Phylogeny

**Fig 6:**
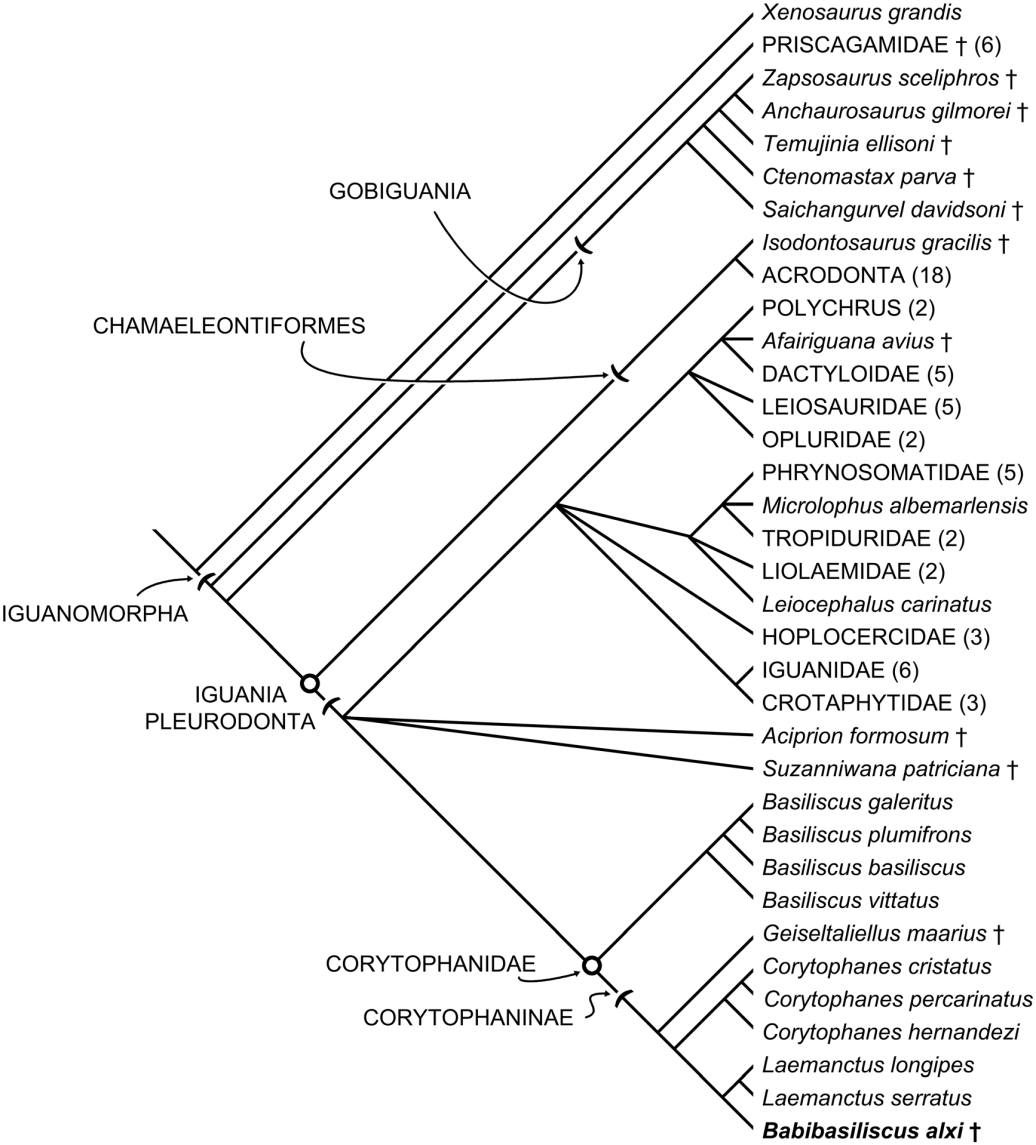
Phylogenetic hypothesis of Iguanomorpha based on the current analysis. This is the Adam’s consensus of 98 trees. *Suzanniwana patriciana* and *Aciprion formosum* are the taxa with the most volatile positions within this analysis.

The phylogenetic analysis recovered 98 shortest-recovered trees of 3153 steps; the rescaled consistency index of 0.2288, and a retention index of 0.5157 (Fig 6). *Babibasiliscus alxi* is the sister taxon to extant *Laemanctus* in all of the recovered trees. *Babibasliscus alxi* and *Laemanctus* form a clade supported by three unambiguous synapomorphies. The jugal lies mostly dorsal to the maxilla, rather than medial to it as in other corytophanids (character 52, state 1). This is also presence in *Aciprion formosum*, sometimes considered to be a corytophanid. A ventromedial process is present on the pterygoid (character 117, state 1). *Aciprion formosum* and *Basiliscus vittatus* also possess this character state. There is no pit on the retroarticular process (character 208, state 1).

**Fig 7:**
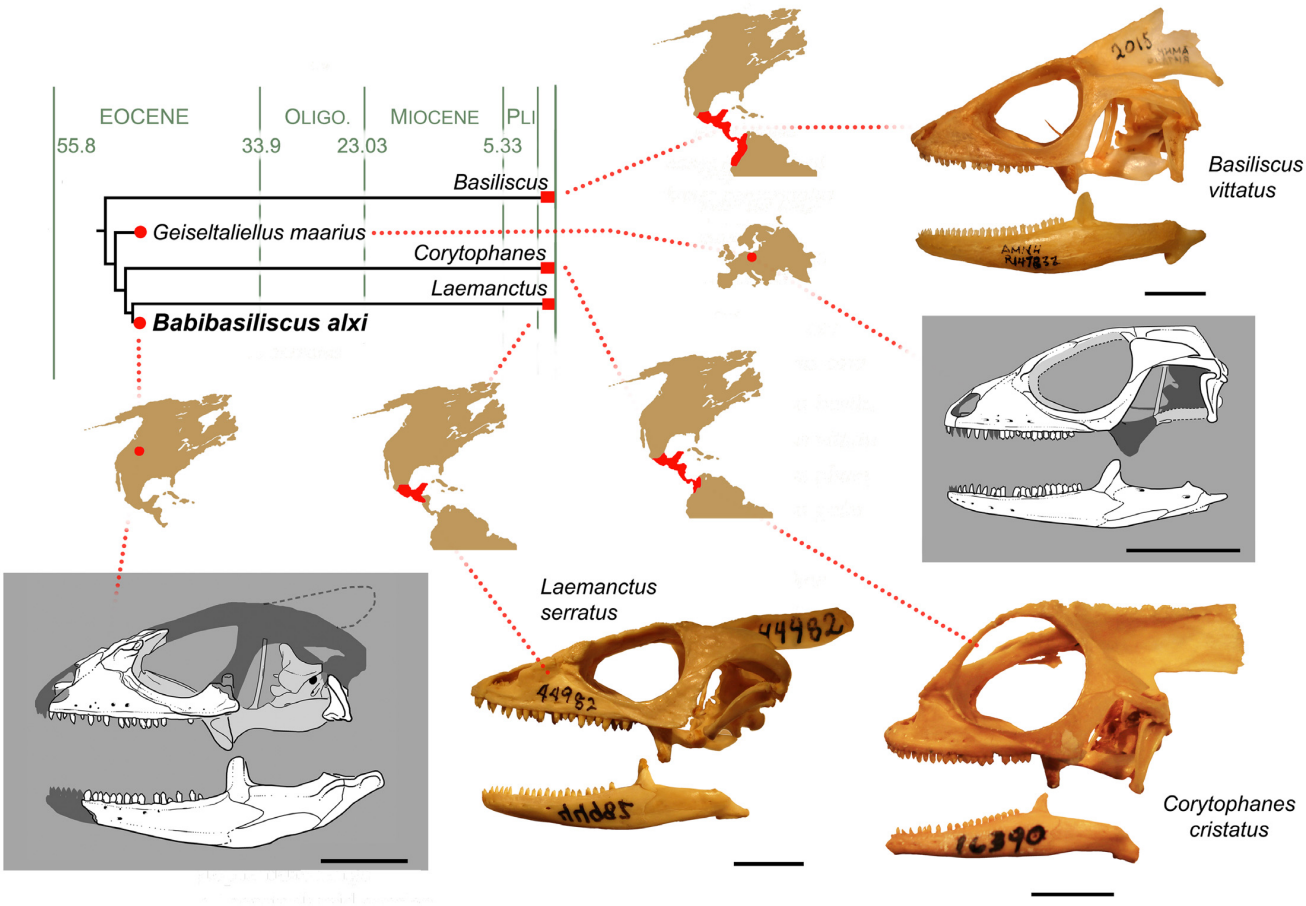
Phylogeny of corytophanids and distribution of anoles, para-anoles, corytophanids, and some malarial infection. (A) Time-calibrated phylogeny of the corytophanid genera with representative illustrations of their skull morphologies.

*Babibasiliscus alxi*, *Laemanctus*, and *Corytophanes* are united to the exclusion of all other taxa in this analysis (Figs 6 and 7) by four unambiguous synapomorphies. Dermal sculpturing is present on the prefrontal (character 9, state 1). The jugal possesses anterior and posterior flanges (character 49, state 1). This character cannot be confidently coded in *Babibasiliscus alxi* and has, therefore, been left coded as “?” in this matrix. Also, this character state is present in *Basiliscus basiliscus* and some specimens of *Basiliscus vittatus*. Rugosities are present on the postorbital process of the jugal (character 50, state 1). This character cannot be coded in *Babibasiliscus alxi*. The postorbital clasps the frontoparietal suture (character 89, state 1). This character cannot be coded in *Babibasiliscus alxi* because the relevant morphology is not preserved.

The three species of *Corytophanes* are united to the exclusion of *Laemanctus* and *Babibasiliscus alxi* in this analysis (Fig 6) by 16 unambiguous synapomorphies. The snout is relatively short, making up less than 30 percent of the basicranial length (character 1, state 0). This character cannot be coded in *Babibasiliscus alxi*. The nasals contact for less than one-half of their total length (character 21, state 1). This condition is also present in some specimens of *Basiliscus vittatus*, and it cannot be scored based on available data for *Geiseltaliellus maarius* (character 24, state 1). The prefrontal extends anteriorly to the external naris, blocking the maxilla from contact with the nasal (character 38, state 0). *Corytophanes* species possess no lateral expansions of the parietals at the frontoparietal sutures (character 72, state 0). This character cannot be confidently coded in *Babibasiliscus alxi*. The pineal foramen occurs within the frontal in *Corytophanes* (character 77, state 1). This character state also occurs in *Basiliscus*. It is considered a convergence between *Corytophanes* and *Basiliscus* because pineal foramen occurs at the frontoparietal suture in the corytophanines *Laemanctus* and *Geiseltaliellus maarius*. This character cannot be scored in *Babibasiliscus alxi*. There is no transverse margin of the parietal between the supratemporal processes (character 82, state 1). Because the parietal is not preserved in *Babibasiliscus alxi*, this character cannot be coded in that taxon. The postorbital is elongate, extending for more than three-quarters of the length of the supratemporal fenestra, in *Corytophanes* (character 96, state 2). This state is also present in *Basiliscus*, but is interpreted as having evolved convergently in that taxon because the postorbital is relatively shorter in *Laemanctus* and *Geiseltaliellus maarius*. This character cannot be coded in *Babibasiliscus alxi*. Meckel’s canal is closed and fused anteriorly/medially in *Corytophanes* (character 181, state 2). This character state is also present in *Basiliscus basiliscus*. The anterior inferior alveolar foramen is not present as a distinct and separate opening in *Corytophanes* (character 183, state 3). There are no distinct surangular and angular dentary processes in *Corytophanes* (character 185, state 0). The clavicles are rod-like, rather than possessing expanded proximal ends, in *Corytophanes* (character 260, state 0). This character cannot be coded in *Babibasiliscus alxi*. The pelvis is strongly sutured, but the individual bones remain partly discernible in *Corytophanes* (character 284, state 1). Most corytophanids possess pelves in which the sutures are obliterated by intergrowth of the bones in adults. This character was not coded in *Basiliscus galeritus*, *Babibasiliscus alxi*, or *Geiseltaliellus maarius*. It was coded as polymorphic for *Basiliscus plumifrons* and *Basiliscus vittatus*. The presence of a complete secondary dorsolateral orbital margin formed by the union of the posterolateral process of the prefrontal with the postorbital (character 373, state 2) is recovered as an unambiguous synapomorphy of *Corytophanes* here. Even so, there is variation within *Corytophanes hernandezi* and that species is coded as polymorphic. A broad jugal-squamosal contact (character 389, state 1) is a *Corytophanes* synapomorphy. This character could not be coded in *Babibasiliscus alxi* or *Geiseltaliellus maarius*. There is no ventral squamosal process (character 390, state 2) in *Corytophanes*. This character could not be coded in *Babibasiliscus alxi* or *Geiseltaliellus maarius*.

I apply the name Corytophaninae to the stem-based clade containing all taxa sharing a more recent common ancestor with *Corytophanes cristatus* than with *Basiliscus basiliscus* (Fig 6). In the present analysis, that clade includes *Geiseltaliellus maarius*, *Corytophanes*, *Laemanctus*, and *Babibasiliscus alxi*, and is united by four unambiguous synapomorphies. The jugal is angulated (character 47, state 0) rather than curved as in *Basiliscus plumifrons* and *Basiliscus galeritus*. Even so, the other two species of *Basiliscus*. There is no postorbital tubercle (character 90, state 0), as there is in all *Basiliscus basiliscus*. This character cannot be scored in *Babibasiliscus alxi*. Absence of an anterior groove associated with the anterior surangular foramen (character 174, state 0) is recovered as an unambiguous corytophanine synapomorphy in this analysis. This means that this character is reversed in *Laemanctus* and convergent in *Basiliscus basiliscus*. The splenial extends for less than one-half of the dentary tooth row (character 189, state 0). This character is reversed in *Babibasiliscus alxi*, which has a relatively elongate splenial.

## Discussion

Iguania (the crown node for Iguanomorpha) has a basal dichotomy (Fig 6) between chameleons, agamas, and their relatives (Chamaeleontiformes), and iguanas, basilisks, anoles, fence lizards, and their relatives (Pleurodonta). Modern iguanians are widely distributed, but the basal phylogenetic dichotomy is reflected in the biogeography of the group, with chamaeleontiforms occurring in the Old World and pleurodontans occurring only in the Americas (except Opluridae from Madagascar and *Brachylophus* from Fiji) [24].

Iguania probably originated in mid- to high-latitudes in Eurasia before dispersal. Modern iguanians occur on every continent except Antarctica and range from southern Canada and central Russia in the north to the southern tip of South America in the south, but their greatest species richness occurs in the tropics [24–26]. The earliest known iguanians come from Cretaceous deposits in Asia [27, 28] and North America [29, 30]. Although a variety of Cretaceous squamates are known from North America [31–34], only a few iguanians have been found there. By contrast, many Cretaceous iguanians or iguanomorphs are known from Asia [10, 27, 28]. Conrad and Norell [27] suggested a rapid iguanomorph diversification during the Cretaceous.

By the Eocene, crown-group pleurodontans must have been relatively diverse as evinced by the presence of the nested *Afairiguana avius* and *Babibasiliscus alxi*, as well as less phylogenetically well-resolved *Suzanniwana patriciana* and *Aciprion formosum* (Fig 6). The Americas have no extant chamaelontiform iguanians, but fragmentary remains demonstrate their presence during the Eocene, suggesting their dispersal across Beringia during the Paleocene and/or Eocene [35].

Perhaps an early pulse of diversification resulted in many basal radiations of Iguanomorpha occurring in Asia, with a few forms dispersing to North America. Subsequent diversification may have led to the origin of many modern pleurodontan groups during the Paleogene. This may have happened primarily in North America based on modern distributions.

The fossil record demonstrates that some terrestrial animals currently confined to the tropics were present in mid- to high-latitudes during warm periods in Earth history [36]. Varanids and shinisaurids are tropics loving taxa that occur primarily in the Old-World [25, 37, 38]. Even so, *Saniwa ensidens* (Varanidae) [31, 39, 40] and *Bahndwivici ammoskius* (Shinisauridae) [41] demonstrate dispersal of these clades into North America before the mid-Eocene. *Necrosaurus cayluxi*, ‘*Necrosaurus*’ *eucarinatus*, and *Necrosaurus feisti* (=*Saniwa feisti*) appear to be European Eocene varanids [11, 12]. These dispersals may also have been via Beringia, indicating that at least varanids, chamaeleontiforms, and shinisaurids possessed much broader Paleogene ranges than they currently enjoy. This may be related to the warmer global temperatures during the Paleogene [42], and their modern (more restricted) ranges may be a product of tropical/equatorial crowding associated with lower global temperatures.

*Babibasiliscus alxi* represents the first conclusive record of a crown-group corytophanid from extratropical North America. Phylogenetic analyses excluding fossils have uniformly proposed a tropical origin for the group [43–45]. This is unsurprising since all extant corytophanids range from equatorial western South America north through most of tropical Central America (Fig 7A and 7B). Consequently, the corytophanid range might be expected to have never been more extensive than the range it currently inhabits, but discovery of *Babibasiliscus alxi* from North America offers additional complexity for the biogeographic history of this clade. The Eocene corytophanids *Geiseltaliellus* and *Babibasiliscus alxi* suggest an ancestral distribution in extratropical Euramerica.

The current distribution of Corytophanidae may represent relicts of southwardly spreading clades (*Basiliscus*, *Corytophanes*, and *Laemanctus*; Fig 7A and 7B). Perhaps some northern iguanian clades went extinct (e.g., Priscagamidae) at the Cretaceous-Tertiary boundary, and others were pushed southward with the global cooling of the Late Tertiary (e.g., Opluridae and Chamaeleontiformes in the Old World; the remainder of Pleurodonta in the Americas).

When viewed in the context of other Eocene fossils representing typically tropics-living squamates, *Babibasiliscus alxi* adds important new data regarding modern distributions of those clades. Corytophanids, along with shinisaurids and varanids, may have been in mid- to high-latitudes because those environments were warmer than today, but also because they were cooler than the (possibly) prohibitively hot equatorial ages in the early Paleogene [46]. Later members of those clades were likely pushed toward the Equator as that area became more suitable and the higher latitudes cooled.

## Supporting Information

S1 File Supporting Information. Dataset A, Morphology Character-By-Taxon Matrix. Here is the full morphological data matrix. Text A, Comparative material. Observations on the following specimens and publications were used for this study. Institutional abbreviations: AMNH, American Museum of Natural History, New York, NY; FMNH, The Field Museum, Chicago, IL; IGM, Institute of Geology, Mongolian Academy of Sciences, Ulan Bator, Mongolia; MCZ, Museum of Comparative Zoology, Harvard University, Cambridge, MA; REE, Richad E Etheridge collection; UCMP, University of California Museum of Paleontology, Berkeley, CA; UC MVZ, Museum of Comparative Zoology, University of California, Berkeley, CA; UF, Florida State Museum, University of Florida, Gainesville, FL; USNM, United States National Museum of Natural History, Smithsonian Institution, Washington, DC; UWBM, Burke Museum of Natural History and Culture, University of Washington, Seattle, WA; YPM, Yale Peabody Museum, New Haven, CT; ZPAL, Zakład Paleobiologii, Polska Akademmia Nauk (Paleobiological Institute, Polish Academy of Sciences), Warsaw, Poland. Text B, Descriptions of Added Morphological Characters. Morphological characters and character-states here newly added to the phylogenetic analysis.
(DOCX)
