# Supplementary material for: A New Eocene Casquehead Lizard (Reptilia, Corytophanidae) from North America
Source: PLoS One. 2015 Jul 1;10(7):e0127900. doi: 10.1371/journal.pone.0127900 (PMC4489568; doi:10.1371/journal.pone.0127900)
Supplement: S1 File — Dataset A, Morphology Character-By-Taxon Matrix. Here is the full morphological data matrix. Text A, Comparative material. Observations on the following specimens and publications were used for this study. Institutional abbreviations: AMNH, American Museum of Natural History, New York, NY; FMNH, The Field Museum, Chicago, IL; IGM, Institute of Geology, Mongolian Academy of Sciences, Ulan Bator, Mongolia; MCZ, Museum of Comparative Zoology, Harvard University, Cambridge, MA; REE, Richad E Etheridge collection; UCMP, University of California Museum of Paleontology, Berkeley, CA; UC MVZ, Museum of Comparative Zoology, University of California, Berkeley, CA; UF, Florida State Museum, University of Florida, Gainesville, FL; USNM, United States National Museum of Natural History, Smithsonian Institution, Washington, DC; UWBM, Burke Museum of Natural History and Culture, University of Washington, Seattle, WA; YPM, Yale Peabody Museum, New Haven, CT; ZPAL, Zakład Paleobiologii, Polska Akademmia Nauk (Paleobiological Institute, Polish Academy of Sciences), Warsaw, Poland. Text B, Descriptions of Added Morphological Characters. Morphological characters and character-states here newly added to the phylogenetic analysis. (DOCX) [file pone.0127900.s001.docx]

**A new Eocene casquehead lizard (Reptilia, Corytophanidae] from North America and its biological implications**

**Author:** Jack L. Conrad^1,2,*^

**Affiliations:** ^1^Department of Anatomy, New York College of Osteopathic Medicine of the New York Institute of Technology, Northern Boulevard, Old Westbury, NY 11568, USA

^2^Department of Vertebrate Paleontology, American Museum of Natural History, Central Park West at 79^th^ Street, New York, NY 10024, USA

**SUPPORTING INFORMATION**

**DATASET A: Morphological Character-by-Taxon Matrix**

Here is the full morphological data matrix.

*Xenosaurus grandis* 1000000111 1101000000 113000?000 0100000010 1000000011 0011102100 0?0?010001 0110?10101 0011010010 0?11001000 1000100110 0010101210 0000010000 0000002001 0010001000 0010000011 2000010001 10[15]011?100 0011100010 01[12]0100000 000?100101 0300000000 1000001101 2001122?00 0011010000 0200000000 1100001000 0001000000 0000201000 11000001?1 0000100000 1000032110 0101?????? 0000?00001 010110000? 011??????? ???3000000 1000111000 000?12?011 000??0?000 ?01?0310?0 02?3600210 0001000?00 04?0??1000 ??000????0 03?00000?? ?????????? ???????0?? ????0010?? ?????????? ??????00?0 1000011100 01001?0000 02000?0101 0000?000?? 00??1??200 100110000? 0010000000 1000000000 00101100?0 0000100000 0000011001 ?2?1100?00 001010010? 0??0000010 0000200010 0001000000 00000?100? 000?000000 0001000010 0011000011 1111220000 0000000000 00?00101?? ??3?0?0000 1100[01]00000 1100111010 1000000000 1100011001 0011000010 011

*Zapsosaurus sceliphros* 1?0???0010 ?????????? ?????????0 ??01?????? 10??001100 01?110210? ???????0?1 111?002?00 ??200001?0 ?1??01?00? ?????00010 00001?1210 00000?0000 000?0??001 0????010?0 0?000???11 ?000?0000? ?000100100 10?1100001 0110?0?000 ?00?000020 0400?00000 0??000???1 2????????? ???1?????? 01???????? ?????????? ?????????? ?????????? ?????????? ?????????? ?????????? ?????????? ?????????? ?????????? ?????????? ???80????? 1?00???0?0 ?0???????? ?????????0 ???10????? ?????????? ?????????? ?????????? ?????????? ?????????? ?????????? ?????????? ?????????? ?????????? ?????????? ?????????? ?????????? ?????????? ???0?000?? 00001??1?? ?000??0??? ?010?000?0 ???00?0000 0?00?????? ????1?0??? ?????????? ?????????? ?????????? ?????????0 ?000000?00 ?0?1?000?? 00???????? 000??????? ?0??0?0??? ????0000?? 011?20?010 00??0000?0 00?0?????? ????0????? ?????????? ?????????? ?????????? ?????????? ?????????? ???

*Anchaurosaurus gilmorei* 100??0?000 110?000000 0030?1?010 ??010000?0 1000001100 01?1102100 0?0?000001 1110002?00 10200001?0 ?????????? ????0????? ?????012?? ??0?000000 0?000??00? ?0?????0?0 00000???11 ?00000000? ?000??0100 11021?0001 011010000? ?00?000020 0400000000 000000???1 2?000?2??? ?????10?00 0000?????? ?00??0???? ????00?0?? ???0??000? ?????????? ????0?0??? ?????????? ?????????? ?????????? ?????????? ?????????? ???80????? ??0????0?? ?????????? ?????????0 ?0??0??0?0 0????????? ?????????? ?????????? ?????????? ?????????? ?????????? ?????????? ?????????? ?????????? ?????????? ?????????? ?????????? ?????????? ?????????? ?????????? ?????????? ?????????? ?????????? ?????????? ?????????? ?????????? ?????????? ?????????? ?????????? ?????????? ?????????? ?????????? ?????????? ?????????? ?????????? ?????????? ?????????? ?????????? ?????????? ?????????? ?????????? ?????????? ?????????? ?????????? ???

*Ctenomastax parvus* 100??00001 110?000000 0130?11010 01010000?0 1?00001100 011110210? 0???0000?1 011?002?00 1010000110 0210011001 0????00?10 000?1?121? ??00000000 0?0??????? ?00??010?0 00000???11 ?00000???? ?0001??000 1?02???011 010010??0? ?00?000020 2000100000 000000???? ?????????? ?????????? ?????????? ?????????? ?????????? ?????????? ?????????? ????0????? ?????????? ?????????? ?????????? ?????????? ?????????? ???800???? ??00?110?0 200?????01 ??0??????0 ????0200?? ?????????? ?????????? ?????????? ?????????? ?????????? ?????????? ?????????? ?????????? ?????????? ?????????? ?????????? ?????????? ?0000?000? 0010?000?? 00001??211 00001?00?? ?010?00000 0?00000000 0?00000000 00001?0??? ?????????? ??????0??? ?0??00???? ???00?001? ?000000?00 ???1?000?? ?????????? 000??????? ????0????? ????0000?1 011?2000?0 0000000000 00?0?????? ?????????? ?????????? ?????????? ?????????? ?????????? ??00?????? ???

*Temujinia ellisoni* 1000?00001 110?000000 ??30?11010 01010?0000 1000001100 011110210? ??????0001 011?002?00 1020000010 0210011001 ???????0?0 00001?1210 00000?0000 00000?0001 001??0?0?0 00000???1? ?000?0000? ?0101?0100 1002100001 0110?0?00? ?00?000?20 0400000000 0??000???? ?????????? ?????????? ?????????? ?????????? ?????????? ?????????? ?????????? ?????????? ?????????? ?????????? ?????????? ?????????? ?????????? ???800?0?0 1?00???0?0 ?00?????0? ??0????1?0 ??110110?? ?????????? ?????????? ?????????? ?????????? ?????????? ?????????? ?????????? ?????????? ?????????? ?????????? ?????????? ?????????? ?????????? ?0?0?000?? 00001??011 00001?00?? ?0100?0000 ???0000000 0?00000??0 0000110??? 0????????? ?????????? ????0????? ???0000010 0000000?00 00??00000? 0000?????? 000??????? ?0010?0??? 00?1??0001 0111200000 0000000000 00?0?????? ????0????? ?????????? ?????????? ?????????? ?????????? ???0?????? ???

*Saichangurvel davidsonae*  0000?00001 110?000000 0030?11010 ??010?0000 1000001100 0111102100 ????0??0?1 111?002?00 0010000110 021001?001 ????100??? ?0??????1? ?0000????? ??00??000? ?0???????? ????????1? ?0???0?00? ??????0??? ?????????? ?1?010???? ????000020 040000?000 ?00000???1 2000002??0 00??0?0?00 01??000??0 111?00?0?0 0???00000? 1000100000 10???????? ?????????? ?????????? ?????????? ?????????? ?????????? ?????????? ???80???0? 1?00?110?0 000?????01 ?????????0 ???10??010 006??????? ?????????? ?????????? ?????????? ?????????? ?????????? ?????????? ?????????? ?????????? ?????1???? ?????????? ?????????? ?00?0?010? ?010?????? 00?????011 00001?0??? ?010000000 ??000?000? 0?00000??0 00001??0?? ?????????? ?????????? ?????????? ?????????? ?0?0000??? ??0?000??? ?????????? ?????????? ?????????? ???????0?? ??1????0?0 0000000??? ???0?????? ??2??????0 ??1?????00 ???0??1000 11000????? ?110???000 0????0???? ???

*Gladidenagama semiplana* ??????2011 ????????00 ?0302??000 01000?00?0 10??001011 001110210? ????0????1 ?11?0???00 0010010010 0???001000 ????10001? 00???01201 ??1000000? 10000??00? ??10???0?0 0??00????? ??????0??? ???0???10? 02?2???00? ?110?0???? ?????????? 010[01]000000 ?00000???? ?????????? ?????????? ?????????? ?????????? ?????????? ?????????? ?????????? ?????????? ?????????? ?????????? ?????????? ?????????? ?????????? ???8?00??? 1?00?01??0 ?0???????? ???????1?? ?0??0100?? ?????????? ?????????? ?????????? ?????????? ?????????? ?????????? ?????????? ?????????? ?????????? ?????????? ?????????? ?????????? ?????????? ?????????? ?????????? ?????????? ?????????? ?????????? ?????????? ?????????? ?????????? ?????????? ?????????? ?????????? ?????????? ?????????? ?????????? ?????????? ?????????? ?????????? ?????????? ?????????? ?????????? ?????????? ?????????? ?????????? ?????????? ?????????? ?????????? ???

*Phrynosomimus asper* 0011002111 1100000000 1030?11000 01010?0000 1000001111 0111102100 0???00?0?1 011??02?00 0000010??0 0???001000 ????000010 0000100210 00100?0000 ??0????1?? ?????210?0 0????????? ??00?0000? ?000??010? 00??0?001? 010010??0? ?00?000?20 0101?00000 ???000???? ?????????? ?????????? ?????????0 1??????000 ?????????? ?????????? ?????????? ?????????? ?????????? ?????????? ?????????? ?????????? ?????????? ???8?????? 1?00???0?0 10??????0? ??0??????? ????0????? ?????????? ?????????? ?????????? ?????????? ?????????? ?????????? ?????????? ?????????? ?????????? ?????????? ?????????? ?????????? ????0?0001 ?010?000?? 0000???21? ?000??00?? ?0?????100 ??000???10 1?00000000 0000?????? ?000?????? ?0???????? ?????????? ??????0??? ?000000?00 ?0???????? ?0???????? 000??????? ?0???????? ????00???? ?????????? ??????0??0 00???????? ?????????? ?????????? ?????????? ?????????? ?????????? ??00?????? ???

*Flaviagama dzerzhinksii* 000??020?1 1102000000 0030211000 01000?00?0 ????00011? 001110210? ????000001 1110?021?0 000001001? 0???001000 ????1000?0 001010120? ?11000?00? ?0000??00? ???0??10?0 0??00???1? ?????0000? ?0????010? 001???0011 0100100??? ?00?000020 0001000000 ?00000???1 2?000?2??? ?????1???? ?????????? ?????????? ?????????? ?????????? ?????????? ?????????? ?????????? ?????????? ?????????? ?????????? ?????????? ???8?00??? 1?00?110?0 100?????0? ??0????1?? ?01?0111?? ?????????? ?????????? ?????????? ?????????? ?????????? ?????????? ?????????? ?????????? ?????????? ?????????? ?????????? ?????????? ?????????? ?????????? ?????????? ?????????? ?????????? ?????????? ?????????? ?????????? ?????????? ?????????? ?????????? ?????????? ?????????? ?????????? ?????????? ?????????? ?????????? ?????????? ?????????? ?????????? ?????????? ?????????? ?????????? ?????????? ?????????? ?????????? ?????????? ???

*Mimeosaurus crassus* 0000?02111 110?000000 0030211010 01010?0000 1000001111 001110210? ????0??0?1 011??02??0 000001??00 0???011000 ????000010 000?1?120? ??10000?00 ??0?0?0??? ??1???10?0 0??00???1? ??0??0000? ?00010010? 001????011 010010??0? ?00??0??20 0101100000 ?00000???1 2?00?????? ?????1???? ?????????? ?????????? ?????????? ?????????? ???????1?? ?????????? ?????????? ?????????? ?????????? ?????????? ?????????? ???80??0?0 ??01?1?0?0 100?????11 ?????????? ??0?0??0?? ?????????? ?????????? ?????????? ?????????? ?????????? ?????????? ?????????? ?????????? ?????????? ?????????? ?????????? ?????????? ?0000????? ?010?000?? 00001??2?? ?000??00?? ?0?????100 ??00010010 1100000000 00001?0??? ????010?0? ??????0??? ????0000?? ???00?0?10 00?0000?0? ???1?0000? ?0?0?????? 000??????? ?0???????? ???10?0??? 0???2??010 ??00?????0 00?0?????? ?????????? ?????????? ?????????? ?????????? ?????????? ??00?????? ???

*Arretosaurus ornatus* ??????2?11 ?????????? ?????????0 ??010?0?10 10??000111 01111?2100 0?0??000?1 011?02??00 0100010010 021001?000 ????0????? ??????02?? ??0???010? 0000?00001 ??00001000 0??00???10 ?000?0?00? ?000??010? 02????000? 0????????? ?00?000?20 0004????00 ???000???1 2000?????0 ??01?????? ?????????0 1????????? ????000001 01???????? ???????1?? ?????????? ?????????? ?????????? ?????????? ?????????? ?????????? ???8???0?? 2?0??????0 ?0???2?011 ?????0?1?0 ??0?0????0 ?????????? ??00?0???? ?????????? ?????????? ?????????? ?????????? ?????????? ?????????? ?????????? ?????????? ?????????? ?????????? ?????????? ?????????? ?????????? ?????????? ?????????? ?????????? ?????????? ?????????? ?????????? ?????????? ?????????? ?????????? ?????????? ?????????? ?????????? ?????????? ?????????? ?????????? ?????????? ?????????? ?????????? ?????????? ?????????? ?????????? ?????????? ?????????? ?????????? ???

*Priscagama gobiensis* 1000?02111 111??00000 1130211010 0101?00000 1000000011 01111?210? 0???0000?? 011??02?00 0100010110 0???011000 0???000010 000?101111 1010000000 0?0????00? ??0???1010 0???????1? ?00??0000? ?000100?00 00020?0011 010010???? ?00?00???0 0101??0000 ?00000???? ?????????? ?????????? ?????????? ?????????? ?0???????? ?????????? ???????1?? ?????????? ?????????? ?????????? ?????????? ?????????? ?????????? ???8???0?0 ??00?1?0?0 10??????11 ???00????0 ????0??0?? ?????????? ?????????? ?????????? ?????????? ?????????? ?????????? ?????????? ?????????? ?????????? ?????????? ?????????? ?????????? ?0000?0?01 1010?0?0?? 00001??2?? ?0001?000? 00?????100 ??00010010 0100000000 00001100?? 0000010?00 ?0?00?0?00 0?0?00000? ???0000010 0000000100 ?001?000?? 0000?????? 000??????? 000?00???? ???1000001 0111200000 0000000??0 0000?????? ?????????? ?????????? ?????????? ?????????? ?????????? ??00?????? ???

*Uromastyx aegyptia* 000001???? 1202000000 1030111000 0101000110 3?0000[01]110 0011102100 0?0?000001 1110002200 0000010000 1???020010 0000010010 0000100202 1100100100 0000010000 0010001001 00000?0011 200000000? ?000100100 01321001?? ?110100000 000?000010 0101100000 ?030000001 2011002?10 0101010000 0200001000 0110000010 011200000? 1001100000 11?100?1?1 00???????? ??000????? 0???0?0?1? ?01??????? ??????0??? ??[01]??????? ???60?0??? ?000???0?0 200?????22 ??0????1?0 ?00?0??0?0 02???1???? ?????????? ?????????? ?????????? ?????????? ?????????? ?????????? ?????????? ?????????? ?????????? ?????????? ?????????? ?[12][01]00?0??1 1010?0?0?? 00001??1?? ?000?1000? 00?????000 ??0001?000 0000000000 00001100?0 0000010010 ?0?00?0?00 000010000? 0??0000010 1000200110 0001000000 000000100? 000?000010 0001001011 10110020?1 0110100000 0000001??1 ???0110100 004?0??000 1010[01]0[01]100 11?0111000 100000111? 0110011000 0000?00010 000

*Agama agama* 100001???? 1102000000 0030111000 0101000000 1001000110 0111102100 0?0?000001 0110002200 0010000101 0???010000 0000000010 0000100210 1010000100 0000010100 0000001001 00000?0011 200000000? 0020110101 01321001?? ?10010?000 000?000020 0101100000 ?030000001 2000002?00 0001010000 0200001000 0101002000 011000000? 1001100000 11?100?1?1 00???????? ??0001???? 0?????0?1? ?????????? ??????0??? ?????????? ???[56]0?000? ?000?010?0 200?????21 ??0????1?0 ?00?0??0?0 004?600??? ?????????? ?????????? ?????????? ?????????? ?????????? ?????????? ?????????? ???0?11101 00022????? ?????????? ?????????? 00220?0?11 1010?0?0?? 00001??320 0000?0000? 00?????300 ??0001?000 0000000000 00001100?0 0000010000 ?0?00?0?00 000000000? 0??0000010 1000000100 0001000001 000000100? 000?000010 0001001011 00110020?1 0110000000 1000000??1 ???0010010 004?0??001 0000110000 11?0111000 1000101000 0110010000 0000?00010 000

*Brookesia brygoii* 0001002011 1202000101 0?30011000 0101?00100 3?00000111 0001103001 0?0?000013 0011?23201 1000020000 0011000000 01??110011 001010021? 1000000100 0201100??1 00201?1000 0001????41 ?200?1000? ?02010?101 01221201?? ?100001000 000?000101 0401000000 ?000001101 ?????????? ?????????? ?????????? ?????????? ?????????? ?????????? ???010?1?1 00010?0??? 00000?00?? 1????????? ?????????? ?????????? ?????????? ???10?0??4 2230111000 120?01?0?0 ??1????1?? ?0?10000?? ?1???00??? ?????21100 00?0??1??? ?????????? ?????????? ?????????? ?????????? ????00000? 020??????? ?????0???? ?????????? ?????????? ?0020?01?1 1000?0???? 10000????? ?00040000? 00?????200 ??0311?000 0000001110 100?0110?0 2????????? ??????0?00 ?00000000? 0??000001? 1000000100 00??000000 ?0110?100? 000????210 0001010011 0011002??? ?110100000 ??00000??1 ???02203?1 ?03?0??001 ??01??0011 11?1110101 011110111? 0111011110 1100?00011 0?0

*Brookesia superciliaris* 0011012011 1?02000001 0030111000 0101?01100 3?01000001 0001100001 0?0?000010 0011023201 0000000000 0??1010000 01??010011 0010100210 1010000000 0201100??0 00000?10?0 0??1???041 2200?1000? 002010?101 01321201?? ?12000?000 ?00?000120 0401000000 ?000001101 210??02?00 0101010010 0200000102 ?0010031?? 0?0000100? 1000001000 4??010?1?1 00000?0??? 0000020000 1?001?0?1? ?110?0???? ??????0??? ?????????? ???10?0?02 2230111000 ?20?03?0?0 ??1??????? ?0?11??0?0 11??300??? ???0?00?00 00?0??1??? ?????????? ?????????? ?????????? ?????????? ????0000?? ?20??????? ?????????? ?????????? ?????????? ?????????? ?????????? ?????????? ?????????? ?????????? ?????????? ?????????? ?????????? ?????????? ?????????? ?????????? ?????????? ?????????? ?????????? ?????????? ?????????? ?????????? ?????????? ?????????? ?????????? ?????????? ?????????? ?????????? ?????????? ?????????? ?????????? ?????????? ???

*Chamaeleo chamaeleon* 0001012011 1112000000 1130111000 0100001100 2001001111 0011104001 0?0?000011 0211001102 ??00020100 0??1010000 01??010011 0011101210 1010000100 0201100??0 0010001000 0001??0041 ?200?0000? 00200?0101 01321201?? ?11000?000 000?2???1? 0401000000 ?0?0001101 2100002?01 0101010000 0200000102 ?0010031?? 0?0000000? 1001001000 40?010?1?0 00000?0??? 00000?0000 1?????0?1? ?????????? ?????????? ?????????? ???40?0002 2120111000 ?20?02?000 ??0????1?? ?0111??0?0 111?300102 11?0?10?00 00?0??0??? ?????????? ?????????? ?????????? ?????????? ?????000?? ?????????? ?????????? ?????????? ?????????? ?????????? ?????????? ?????????? ?????????? ?????????? ?????????? ?????????? ?????????? ?????????? ?????????? ?????????? ?????????? ?????????? ?????????? ?????????? ?????????? ?????????? ?????????? ?????????? ?????????? ?????????? ?????????? ?????????? ?????????? ?????????? ?????????? ?????????? ???

*Rieppeleon brachyurus* 0000002011 1212000000 1131???000 0?00000000 3?01000011 0001101001 0?0?0100?1 0110003102 ??00011?00 0011010000 01??010011 001010?210 1010000000 0201100??0 00?0?01000 0001???041 ?200?0000? ?020??0101 01321201?? ?010?0?00? ?00?0001?0 0401000000 ?0?0001101 ?????????? ?????????? ?????????? ?????????? ?????????? ?????????0 ????00?1?1 00000?0??? 00000????? 1????????? ?????????? ?????????? ?????????? ???50?00?2 2100111000 ?20?11???0 ??0????1?? ???11??0?? ?1???1???? ???0??0?00 02?0??0??? ?????????? ?????????? ?????????? ?????????? ?????000?? ?????????? ?????????? ?????????? ?????????? ?????????? ?????????? ?????????? ?????????? ?????????? ?????????? ?????????? ?????????? ?????????? ?????????? ?????????? ?????????? ?????????? ?????????? ?????????? ?????????? ?????????? ?????????? ?????????? ?????????? ?????????? ?????????? ?????????? ?????????? ?????????? ?????????? ?????????? ???

*Rhampoleon boulengeri* 0101012011 1112000000 11302??000 0101000100 3?01000011 001110000? 0?0?010111 011?003102 ??00021?00 0011010000 01??000011 0010101210 1000000000 0201100??0 0000001000 0001????41 ?100?0000? ?020100101 01320?01?? ?11000?00? ?00?000100 0401000000 ?00000???? ?????????? ?????????? ?????????? ?????????? ?????????? ?????????? ????00?1?1 00000?0??? 00000????? ?????????? ?????????? ?????????? ?????????? ???50?00?2 2200011101 12???1?010 ??0????1?0 ?1110000?? ?1???0???? ???0??0001 08?0?00??? ?????????? ?????????? ?????????? ?????????? ????0000?? ?????????? ?????????? ?????????? ?????????? ?????????? ?????????? ?????????? ?????????? ?????????? ?????????? ?????????? ?????????? ?????????? ?????????? ?????????? ?????????? ?????????? ?????????? ?????????? ?????????? ?????????? ?????????? ?????????? ?????????? ?????????? ?????????? ?????????? ?????????? ?????????? ?????????? ?????????? ???

*Rhampholeon spectrum* 0000012011 11[01]2000000 1130111000 0101000100 3?01000001 0001100000 0?0?000011 0110003102 ??0001[01]000 0??1010000 01??0[01]0011 0010100210 1010000000 0201100??0 00000?10?0 0??1???041 2200?1000? 0020???101 01320?01?? ?11000?000 ?00?000120 0401000000 ?000001101 2100002?00 0101010010 0200000102 ?0010031?? 0?0000100? 1000001000 4??000?1?1 00000?0??? 0000020000 1?00??0?1? ???0?0???? ??????0??? ?????????? ???50?0??? ?100?110?0 ??0??????0 ?????????? ?0?11??0?0 11???00??? ???0??0?01 08?0??0??? ?????????? ?????????? ?????????? ?????????? ????0000?? ?????????? ?????????? ?????????? ?????????? ?????????? ?????????? ?????????? ?????????? ?????????? ?????????? ?????????? ?????????? ?????????? ?????????? ?????????? ?????????? ?????????? ?????????? ?????????? ?????????? ?????????? ?????????? ?????????? ?????????? ?????????? ?????????? ?????????? ?????????? ?????????? ?????????? ?????????? ???

*Phrynocephalus maculatus* 000000?000 1100000000 103000?000 0101000010 3?01001000 001110210? 0?0?0000?0 111?00?000 0010000101 ????010000 0000000010 0010100210 1000000000 000100000? 0000001000 00000?0011 ?00000000? ?020100101 01321101?? ?10000?00? ?00?000020 0101100000 ?00000???1 2????????? ?????????? ?????????? ?????????? ????00?0?? ?????????0 ????00?1?1 00000?0??? 00000????? 1????????? ?????????? ?????????? ?????????? ???[45]0?00?2 1000111000 00??02?022 ??1??0?000 ?00?1100?? ?2???00??? ??????1100 06?1?01??? ?????????? ?????????? ?????????? ?????????? ?????????? ?????????? ?????????? ?????????? ?????????? ?????????? ?????????? ?????????? ?????????? ?????????? ?????????? ?????????? ?????????? ?????????? ?????????? ?????????? ?????????? ?????????? ?????????? ?????????? ?????????? ?????????? ?????????? ?????????? ?????????? ?????????? ?????????? ?????????? ?????????? ?????????? ?????????? ?????????? ???

*Hypsilurus papuensis* 000001?000 1102000000 013021?010 0001000000 1001000010 0011102100 0?0?011011 021?002200 0000000111 0110010000 0000010010 0010101210 1010000000 ?0000001?1 00100010?0 00000?0011 ?00000010? ?020100101 01321101?? ?11000?00? ?00?000010 0401100000 ?00000???? ?????????? ?????????? ?????????? ?????????? ?????????? ?????????? ????00?1?0 00100?0??? 01000????? 1????????? ?????????? ?????????? ?????????? ???90?00?0 2000?00000 200?02?022 ??0??0?100 ?0010310?? ?0???1???? ???1011101 010?0????? ?????????? ?????????? ?????????? ?????????? ?????????? ?????????? ?????????? ?????????? ?????????? ?????????? ?????????? ?????????? ?????????? ?????????? ?????????? ?????????? ?????????? ?????????? ?????????? ?????????? ?????????? ?????????? ?????????? ?????????? ?????????? ?????????? ?????????? ?????????? ?????????? ?????????? ?????????? ?????????? ?????????? ?????????? ?????????? ?????????? ???

*Leiolepis reevesii* 000000?000 1100000000 0020211000 0101000000 1000001100 0111102000 0?0?0000?1 0212003200 0010000010 0110011001 0000000010 0010101211 0000010000 0000000001 0010001000 00000?0011 ?00000000? ?00110?101 01320?01?? ?10000?000 ?00?000010 0401100000 ?00000???? ?????????? ?????????? ?????????? ?????????? ?????????? ?????????? ????00?1?1 00000?0??? 00000?00?? 0?????0?1? ?????????? ?????????? ?????????? ???80?00?0 10001120?0 ?00?01?010 ??0????000 ?0011100?? ?0???1???? ??00?01111 12?1??0??? ?????????? ?????????? ?????????? ?????????? ?????????? ?????????? ?????????? ?????????? ?????????? ?????????? ?????????? ?????????? ?????????? ?????????? ?????????? ?????????? ?????????? ?????????? ?????????? ?????????? ?????????? ?????????? ?????????? ?????????? ?????????? ?????????? ?????????? ?????????? ?????????? ?????????? ?????????? ?????????? ?????????? ?????????? ?????????? ?????????? ???

*Physignathus cocincinus* 000000?000 1100000000 1030111000 0001000000 1001001010 0011102000 0?0?000001 0210002100 0010000000 0???020000 0000000010 0000101210 1000000000 0000010000 0000001000 0000000011 ?00000000? ?020100001 01321101?? ?12010000? ?00?000020 0401100000 ?03000??01 2????????? ?????????? ?????????? ?????????? ?????????? ?????????? ???100?1?0 00000?0??? ?0000????? 0?????0?1? ?01??????? ?????????? ?????????? ???90?01?0 1000?000?0 000??????1 ??0????1?0 ?0010010?? 00???00??? ??????0?01 08?0?00??? ?????????? ?????????? ?????????? ?????????? ?????????? ???0?01110 00000????? ?????????? ?????????? ?1120?0?11 1000?????? 00001??320 0000?0000? 00?????200 ??00010000 0000000000 00001100?0 0000010000 ?0?00?0?00 000000000? 0??0000010 1000000100 0001000001 000000100? 000?000010 0001001011 10110020?1 0111000000 1000000??1 ???0020110 004?0??000 1100010000 11?0111000 100010???? 0110011000 0000?00010 000

*Intellagama lesueurii* 000000?000 1110000000 1030211011 0001000010 1001001010 0111102100 0?0?100001 0210002200 0[01]00000100 0???020000 ?00?000010 0000101210 1010000000 0000010000 0010001000 00000?0011 ?0000????? ?????????? ?????????? ?????????? ?????????? 0401100000 ?00000???1 21020?2??? 0?01010010 020000???1 0100002000 000100000? 1001100000 1????????? ?????????? ??000????? 0?????0?1? ?????????? ?????????? ?????????? ???90?0?00 1000010000 100?12?012 ??0??0?1?0 ?0?10200?0 00???00112 11?1001101 08?0?01??? ?????????? ?????????? ?????????? ?????????? ?????????? ?????????? ?????????? ?????????? ?????????? ?????????? ?????????? ?????????? ?????????? ?????????? ?????????? ?????????? ?????????? ?????????? ?????????? ?????????? ?????????? ?????????? ?????????? ?????????? ?????????? ?????????? ?????????? ?????????? ?????????? ?????????? ?????????? ?????????? ?????????? ?????????? ?????????? ?????????? ???

*Amphibolurus muricatus* 1000002001 1100000000 0030211010 0001000010 3??1001010 0011102000 0?0?100001 0210002200 0010000100 0100020001 000?000010 0000100210 1000000000 0000010001 0010001000 00000?0011 ?00000010? ?020100001 01321101?? ?10010?10? ?00?000020 0401100000 ?03000???? ?????????? ?????????? ?????????? ?????????? ?????????? ?????????? ?????????? ?????????? ??000????? 0????????? ?????????? ?????????? ?????????? ???90?00?2 1100011000 100?02?022 ??0??0?100 ?0011010?? ?????00??? ???1001101 1??0?????? ?????????? ?????????? ?????????? ?????????? ?????????? ?????????? ?????????? ?????????? ?????????? ?????????? ?????????? ?????????? ?????????? ?????????? ?????????? ?????????? ?????????? ?????????? ?????????? ?????????? ?????????? ?????????? ?????????? ?????????? ?????????? ?????????? ?????????? ?????????? ?????????? ?????????? ?????????? ?????????? ?????????? ?????????? ?????????? ?????????? ???

*Pogona barbata* 0000012001 1000000000 103010?010 0001000000 3??1000010 0011102000 0?0?000011 0112012000 0000000101 0???020000 0000000010 0000100210 1110000000 0000010001 0010001000 0000000011 ?00000000? ?020100001 0132110001 010010?00? ?00?000020 0101100000 ?00000??01 2001002?00 0001010000 0200001?01 0100001000 011100000? 1001100000 21??0??1?1 0????????? ??000????? 0????????? ?????????? ??????1??? ?????????? ???9000010 2000111000 020?02?021 ??0????1?0 ?00?0??0?0 027?600??? ???1000?11 0??0?????? ?????????? ?????????? ?????????? ?????????? ?????????? ?????????? ?????????? ?????????? ?????????? ?????????? ?????????? ?????????? ?????????? ?????????? ?????????? ?????????? ?????????? ?????????? ?????????? ?????????? ?????????? ?????????? ?????????? ?????????? ?????????? ?????????? ?????????? ?????????? ?????????? ?????????? ?????????? ?????????? ?????????? ?????????? ?????????? ?????????? ???

*Calotes versicolor* 0000012000 1202000000 113010?000 0101000000 1001000111 0011102000 0?0?000001 0211012?00 0010000100 0110010000 01??000010 0010100210 1010000000 0000000001 0010011001 00001?0011 ?00000010? ?020100001 0132120001 011010?00? ?00?000000 0401100000 ?00000???1 2000?02?02 0001010000 02000000?0 01000010?? 0?1100200? 1000100000 11??00?0?0 00100?0??? 01000????? ?????????? ?????????? ??????0??? ?????????? ???[48]000000 2000010000 100?02?022 ??1??0?100 ?1110010?0 004?6001?? 1??1010?01 06?0?01??? ?????????? ?????????? ?????????? ?????????? ????00000? 02???????? ?????0???? ?????????? ?????????? ?20000011? 001??????? 00001??020 00?010000? 001??00300 ???00?00?? 0?0000?000 000001?0?? 0????????? ?????????? ??00000??1 ?000?00110 0000?00100 00010000?? 000??????? ???????01? ??0100???? ??110120?0 011?1?0000 0000000??1 ???0?????? ??3???0001 ??10000001 ??10???0?0 1000?????? 0100???00? 0?00?0???? ???

*Lyriocephalus scutatus* 0000012001 1202000000 1030210000 0101000010 3001001111 0011102101 0?0?000011 021?012?00 0020020102 1???010000 01??000011 1010101210 1010000110 ?200000??? ?000001001 00000?0011 ?10000000? ?020110101 0132120000 012000000? ?00?100010 0401100000 ?01000???? ?????????? ?????????? ?????????? ?????????? ?????????? ?????????? ?????????? ??0?0?0??? 01000????? 0????????? ?????????? ?????????? ?????????? ???70100?2 1020200000 010?01?022 ?????0?100 ?2111000?? ?1???00??? ???1020000 08?0?01??? ?????????? ?????????? ?????????? ?????????? ????00000? 01???????? ?????0???? ?????????? ?????????? ?00?010011 0010?????? 000?0????? ?0?010000? ?02??00200 0??01??0?? 0?0000?000 00001100?? 0????????? ?????????? ??0?0000?1 0??0?00010 0000000?00 010100???? ??00?????? 0???????10 ??0100???? ??1100?0?1 01100?0000 0020100??1 ???0?????? ?????????? ?????????? ?????????? ?????????? ?????????? ???0?????? ???

*Draco blanfordi* ?????1?000 1????????? ?????0?000 0101000000 1001001110 0011102000 0?0?000001 0111012200 0020000100 0110020000 01??000011 0010101210 1010000110 0200000??1 0010011010 00001?0011 ?000000100 2020100001 0132120001 011010?000 000?000100 0401100000 ?03000???1 20000?2??? 0??10?0?00 02??000?0? ?10?000??? 0????????? ???0100??? ????00?1?1 000?0?0??? 00000????? 0????????? ?????????? ?????????? ?????????? ???8000000 20001010?0 ?01102?021 ??0????100 ?0110000?? 00???00??? ??????1101 0??0??0??? ?????????? ?????????? ?????????? ?????????? ????01000? 00???????? ?????0???? ?????????? ?????????? ?00?00001? ?????????? 00001??020 00?021000? 002??00300 0??01?00?? 0?0000?000 000001?0?? 0????????? ?????????? ?????????? ???0????10 00????0100 ??01000??? ?????????? ???????11? ?????????? ????0020?1 011?0?0000 2020000??1 ???0?????? ?????????1 ??1??????? ?????????? ?????????? ?????????? ??00?????? ???

*Hydrosaurus amboiensis* 100001?000 1100000000 013010?010 0001000010 1010000010 0011102000 0?0?100011 0110112200 0100000100 0110020000 0000000010 0000101210 1000000000 0000010000 0010001010 0000000011 ?00000000? ?020110101 01320?0001 010010?000 000?000010 0101100000 ?030000001 21010?2?10 0?01010010 020000??00 0100011000 000100000? 1001100000 01??0??1?0 0????????? ??000????? 0????????? ?????????? ?????????? ?????????? ???[89]000000 20000?1010 220?02?011 ??0??0?100 ?00?0200?0 00??61???? ???1010?01 14?1?01??? ?????????? ?????????? ?????????? ?????????? ?????????? ?????????? ?????????? ?????????? ?????????? ?????????? ?????????? ?????????? ?????????? ?????????? ?????????? ?????????? ?????????? ?????????? ?????????? ?????????? ?????????? ?????????? ?????????? ?????????? ?????????? ?????????? ?????????? ?????????? ?????????? ?????????? ?????????? ?????????? ?????????? ?????????? ?????????? ?????????? ???

*Enyalius bilineatus* 100?000111 1100000000 1030010010 01010000?0 1000001110 0001102101 0?0?000011 0112002110 0010000011 0110010000 0000010011 0010201110 1010000000 0000000000 00100?1000 00000???11 ?000?0000? ?001110100 20220?0001 011010000? ?00?000020 0400000000 0000000001 20000?2?10 0??1010??? ????000??1 1100000000 000000000? 1000100000 01??10?1?0 00000?0??? ?0000????? 0????????? ?????????? ?????????? ?????????? ????000010 1000?1?0?0 100??????1 ??0??????? ??010210?? ?06??????? ??????1101 15?0?01??? ?????????? ?????????? ?????????? ?????????? ????001000 02???????? ?????0???? ?????????? ?????????? ?00000001? 0010?000?? 00000??020 000011000? ?010000000 0??00?00?? 0?0000?000 00000100?? ?????????? ?????????? ?????0???1 01?0?00010 0000000100 00110000?? 0000?????? 000????2?0 ??0?00?0?? ??1?00?0?1 0112200000 0000000000 00?0?????? ??3???0001 1210??0000 ???0???0?0 1?000010?? 0??0????0? 0??0??0011 ???

*Diplolaemus darwinii* 1000010011 1100000000 1030110010 0101000000 1000001001 0011102100 0?0?000011 011?002110 0010020100 0110011000 0000000010 0010101210 101000000? 0000000001 0000001000 0?000?0010 ?00000000? ?0300?0001 2002100001 011010100? ?00?000120 0400000000 000000???1 2000002?10 0???010000 0?00001??1 111?000000 001?00000? 1000200000 01??00?1?1 00000?0??0 00000????? 0????????? ?????????? ?????????? ?????????? ???20?0010 1000111000 001002?001 ??0??0?100 ?011030010 006??00??? ??00?11111 15?1?00??? ?????????? ?????????? ?????????? ?????????? ????00100? 0?3??????? ?????0???? ?????????? ?????????? ?000000000 0010?????? 000?0??020 001011000? 0010?00000 ???00?00?? 000000?000 00010100?? 0?0001???? ?????????? ????00???1 01?0?00010 1000?00100 00110000?? 0000?????? 000????210 ?00100???? ??1100?0?0 0?122?0000 0001000000 00?0?????? ??3?0?0000 1?10??0??0 ?11010?000 1?00?????? 011001?000 01?????0?1 ???

*Leiosaurus catamarcensis* 1000012011 1110000000 1030110000 0101000000 1000000111 0011102100 0?0?000011 0110002000 0010020111 0100010000 0000000010 0010100110 1010000100 2000000001 0000001000 00000?0010 ?000000000 1001110100 2011110001 0110100000 000?000122 0400000010 000000000? ?????????? ?????????? ?????????? ?????????? ?????????? ?????????? ?????0?1?1 00000?0??0 ?0000????? ?????????? ?????????? ?????????? ?????????? ???20?00?0 10001?1000 101001??01 ??0000?000 ?01?0110?? ?2???00??? ???0?00011 15?1?01??? ?????????? ?????????? ?????????? ?????????? ????0010?? ?????????? ?????0???? ?????????? ???0?????? ?2000?0001 0010?000?? 00001??020 000011000? 0010000000 0?00000010 0000000000 00001100?0 0000010000 ?0?00?0?00 000000000? 0??0000010 0000000100 0001000000 00000?100? 000?000010 0001001010 0011000001 0112200000 0000000000 00?0010100 0?3?0?0000 ?????????? 11?011?0?0 ?????????? 01?001?00? 0?00?00011 0??

*Urostrophus vautieri* 1000010111 110?000000 1030010000 0101000000 1000001110 0001102101 0?0?000001 1112002?00 0010000110 011001???? ?????????? ?????????? ?????????? ?????????? ?????????? ?????????? ?????????? ????1????? ?????????? ?????????? ?????????? ?????????? ?????????? ?????????? ?????????? ?????????? ?????????? ?????????? ?????????? ?????????? ?????????? ?????????? ??????1?0? ?????????? ?????????? ?????????? ?????????? ???????0?? ?????????? ?????????? ?????????? 0????????? ?????????? ?????????? ?????????? ?????????? ?????????? ?????????? ????????0? 0?3??????? ?????????? ?????????? ?????????? ?0000?0001 0010?000?? 00?????020 000011000? 0010000000 0?00000010 0000000000 00001100?0 0000010000 ?0?00?0?00 000000000? 0??0000010 0000000100 0001000000 000000110? 000?000210 0001000010 0011000001 0112200000 0000000000 00?0010100 ??3?0?000? ?????????? 11?011?0?0 ?????????? 01?001?00? 0?00?00011 0?0

*Anisolepis grilli* 1001012111 111?000000 ?030010000 0101000000 1000000111 0001102101 0?0?000001 0112002000 0010000110 01[01]0010000 00??110011 0010201110 1010000000 00000001?? 00000?1000 00001?0011 ?00000000? ?001110100 21020?0000 011010000? ?00?100120 0400000000 0000000001 2000002?10 0??1010000 03000001?1 110?001000 000?00000? 1000100000 ?1???????? ?????????? ?????????? ??????1?0? ?????????? ?????????? ?????????? ???2000000 1?00?1?0?0 1011?????1 ??000????0 ?0010310?? 006??00??? ?????????? ?????????? ?????????? ?????????? ?????????? ?????????? ????????0? 0????????? ?????????? ?????????? ?????????? ??????0001 0010?000?? ?????????? ?????????? ?????????? ?????????? ?????????? ?????????? ?????????? ?????????? ?????????? ?????????? ?????????? ?????????? ?????????? ?????????? ?????????? ?????????? ?????????? ?????????? ?????????? ?????????? ?????????? ?????????? ?????????? ?????????? ?????????? ???

*Polychrus marmoratus* 100000[02]011 1110000000 10?0?0?000 0101000010 1000000111 0001102101 0?0??000?1 011?003000 0000000111 0???010000 ?0??100010 0000201110 1010000000 00000101?1 00200?1000 0000000011 ?00000000? ?0001?0101 20?2120000 011010000? ?00?100120 0400000000 0000000001 2000012?10 0001010000 0?00000100 1110002000 010000000? 1000101000 01??10?1?1 0???0?0??? ?0000?1??? 0?????1?0? ?01??????? ?????????? ?????????? ???300000? ?00????0?? ?????????? ???00??1?0 ?0??0??0?? 00???00??? ?????????? ???????0?0 ??1?0????0 02?00?1??? ?????????? ?????????? ????????0? 020??????? ?????????? ?????????? ?????????? ?2000?000? 0010?000?? 00?????02? ?00011000? 0010000??? ???0002001 0?00000000 00001100?0 00000100?0 ???00?0?00 000000000? 0??0000010 0000000100 0001000000 000000100? 000?000010 0001000010 0011000001 1212200000 0000000000 00?0010010 004?0?0000 [01]0001[01]0100 11?0111000 1000001000 0110011000 0000?00011 000

*Polychrus femoralis* 100000[02]011 11?0000000 ?0?000?000 0101000010 1000000001 0011102101 0?0?100001 0110002000 0010000111 0???010000 000?100010 0010201[12]10 1010000000 0000010001 0010001000 0000000011 200001000? ?00011?100 2002100000 011010?000 000?000020 0400000000 0000000001 2000002?10 00?1010000 ?30000110? 1100002000 0?0000000? 1000000000 01011001?0 00001?0??0 00000?1000 00?01?1?0? ?01??????? ?????????? ?????????? ???[23]00000? ?00????0?? ????????0? ???00????0 ????0??0?? 00???00??? ?????????? ?????????? ?????????? ?????????? ?????????? ?????????? ????????0? 0?3??????? ?????????? ?????????? ?????????? ?????????? ?????????? ?????????? ?????????? ?????????? ?????????? ?????????? ?????????? ?????????? ?????????? ?????????? ?????????? ?????????? ?????????? ?????????? ?????????? ?????????? ?????????? ?????????? ?????????? ?????????? ?????????? ?????????? ?????????? ?????????? ?????????? ?????????? ???

*Afairiguana avius* 100??0???? 11?000?000 ?03??????? ??0???0??? ????00???? 0???1021?1 0??????001 0?10?02??? ???0?0??0? 0??00??00? ??????0?10 00????1?1? ?????????? ?????????? ?????????? ?????????? ?000?0???? ?????????? ???2??0??? ?1??????0? ?0??000?20 0?00?00000 ?0000????1 2?00?02??? 0???010??0 030000?1?1 1???00???? 0???00000? ?00010?000 ?????????? ????0?0??? ?????????? ?????????? ?????????? ?????????? ?????????? ???3?????? ?????1???? ?????????? ???00????0 ?????????0 006??????? ?????????? ?????????? ?????????? ?????????? ?????????? ?????????? ?????????? ?????????? ?????????? ?????????? ?????????? ?????????? ?????????? ?????????? ??0??????? ??????0??0 ?????????? ?????????? ?????????? ?????????? ?????????? ?????????? ?????????? ?????????? ?????????? ?????????? ?????????? ?????????? ?????????? ?????????? ??????00?? ?????????? ?????????? ??0??????0 ???0???000 10000????? ??1????000 00???????1 ???

*Anolis carolinensis* 100000[12]111 1210000000 ?030110010 0101000000 1000000011 0011100101 0?0?000001 0110002001 0000000100 0110010000 0000100110 0000101211 0010000000 0000000001 0000001000 00000?0011 ?00000000? ?0011?0101 20120?01?? ?110101000 000?001020 0400000000 0000000001 2000002?10 0001010000 030000?100 1110002000 000000000? 1000100000 01?00011?0 10000?0??? ?000001000 0????????? ?????????? ??????0??? ?????????? ???30?0?00 100011???? ?????????? ?????????? ?????????? ???????1?? 1????????? ?????????? ?????????? ?????????? ?????????? ?????????? ?????????? ?????????? ?????????? ?????????? ?????????? 00000?0001 0010?000?? 00001??020 000011000? 0010000000 0?00000011 0000000000 0000110000 000?010010 ?0?00?0?00 000000000? 0??0000110 0000000100 0011000000 000000100? 000?000010 0001001010 1011000??? ?212200000 0000000000 00?0010110 004?0?0000 0[02]00010000 11?0111000 1000001000 0110011000 0000?00011 0?0

anoles in amber 100?00???? ??0?000000 ?030?????? 0?01??00?? ????000??? 0???102101 0?0?0000?? ?????????0 ????0?010? ?1??01?00? 0???0001?0 00001?1210 ?0000?0??0 0?00??000? ??0????0?0 0???????1? ?000?????? ???????10? ?????????? ?11??????? ??0??00?20 0400000000 ?000000??1 2000??2??? ???1010000 03??000?00 110?00?000 ????00000? ????100000 ?1??00?1?? 00000?0??? 000?0????? ?????????? ?????????? ?????????? ?????????? ???3?????? ??0??????? ?????????? ?????????0 ????0??0?0 00???????? ?????????? ?????????? ?????????? ?????????? ?????????? ?????????? ?????????? ?????????? ?????????? ?????????? ?????????? ?????????? ?????????? ?????????? ?????????? ?????????? ?????????? ?????????? ?????????? ?????????? ?????????? ?????????? ?????????? ?????????? ?????????? ?????????? ?????????? ?????????? ?????????? ?????????? ?????????? ?????????? ?????????? ?????????? ?????????? ?????????? ?????????? ?????????? ???

*Phenacosaurus heterodermus* ?00000[01]111 1100000000 103000?010 0101000000 1001000011 0111102101 0?0?000001 0110002001 0000010001 0110010000 ?0??000110 00101?1210 11????0100 00000101?0 0000001000 0000000?11 ?000?0000? ?0011?0101 20321201?? ?1?010?00? ?00??0???? 0400000000 000000???? ?????????? ?????????? ?????????? ?????????? ?????????? ?????????? ???0?011?0 00000?0??? 0000?01000 0?????1?0? ?01??????? ?????????? ?????????? ????0?00?? ?00????0?? ?????????? ?????????0 ????0??0?? 00???1???? ?????????? ?????????? ?????????? ?????????? ?????????? ?????????? ????????0? 003??????? ??????00?1 0000011100 0000010000 0????????? ?????????? ?????????? ?????????? ?????????? ?????????? ?????????? ?????????? ?????????? ?????????? ?????????? ?????????? ?????????? ?????????? ?????????? ?????????? ?????????? ?????????? ?????????? ?????????? ?????????? ?????????? ?????????? ?????????? ?????????? ?????????? ?????????? ???

*Deiropteryx occulta* 101000?000 1300000000 003000?010 0101000000 1000000000 0011102101 0?0?000001 0110002001 0000000001 0210010000 ?00?000110 0000101110 1000000000 0000000001 0000001001 0000000011 ?000?0000? ?0311?0101 20120?0001 0110101000 000?000020 0400000000 0000000001 2000002?10 0001010000 0300000100 1100001000 000000000? 1000000000 01?0?011?0 00000?0??? 00000?1?0? 0?????1?0? ?????????? ?????????? ?????????? ???300001? ?00????0?? ?????????? ???00????0 ?0??0??0?0 00???00??? ?????????? ?????????? ?????????? ?????????? ?????????? ?????????? ????????0? 003??????? ?????????? ?????????? ?????????? ?????????? ?????????? ?????????? ?????????? ?????????? ?????????? ?????????? ?????????? ?????????? ?????????? ?????????? ?????????? ?????????? ?????????? ?????????? ?????????? ?????????? ?????????? ?????????? ?????????? ?????????? ?????????? ?????????? ?????????? ?????????? ?????????? ?????????? ???

*Deiropteryx vermiculata* 101?000111 1300000000 0030010110 0101000000 1000000011 0011102101 0?0?100001 0110100?01 0000000101 0110010000 ?00?000110 0000101100 1100000000 00000?01?1 ?000001000 0000000011 ?000?0000? ?001110101 2032100000 011010100? ?10?001020 0400000000 0000000001 2000002?10 0?01010000 0?00001??0 1110001000 000000000? 1000100000 01?0?011?0 00000?0??? 00000?1?0? 0?????1?0? ?????????? ?????????? ?????????? ???3000?0? ?00????0?? ?????????? ???00????0 ?0??0??0?0 00???00??? ?????????? ?????????? ?????????? ?????????? ?????????? ?????????? ????????0? 023??????? ?????????? ?????????? ?????????? ?????????? ?????????? ?????????? ?????????? ?????????? ?????????? ?????????? ?????????? ?????????? ?????????? ?????????? ?????????? ?????????? ?????????? ?????????? ?????????? ?????????? ?????????? ?????????? ?????????? ?????????? ?????????? ?????????? ?????????? ?????????? ?????????? ?????????? ???

*Isodontosaurus gracilis* 000??0?000 1310000000 0030?11000 ??010?00?0 1000001100 001110210? ????1??001 1110002?00 001000??00 0101010000 ?????????? 0???1002?? ?0???00??? 0??00????? ??1????0?? ????????1? ?000?00??? ?02010?100 0010??0011 011?1????? ?010000?10 0500000000 000000???? ?????????? ?????????? ?????????? ?????????? ?????????? ?????????? ?????????? ?????????? ?????????? ?????????? ?????????? ?????????? ?????????? ???800?0?? ??00?10??0 000??????1 ?????????0 ??0?0??0?? ?????????? ?????????? ?????????? ?????????? ?????????? ?????????? ?????????? ?????????? ?????????? ?????????? ?????????? ?????????? ????0?000? ?010?000?? 00001??1?? ?100??0??? ?010?00000 ??00000000 0?00?????? ????110??? ???????0?? ?????????? ????0????? ?????????0 00000001?0 ?0?1?000?? 0000?????? 000??????? ?00?0????? ???1002001 01112?0010 0000000000 00?0?????? ????0????? ?????????? ?????????? ?????????? ?????????? ??00?????? ???

*Leiocephalus carinatus* 0000000111 1100000000 003001?000 0101000000 1000001100 0111102100 0?0?000001 0110002200 0000000110 0110010000 0001100010 0000101200 1000010000 00000101?1 0010001000 0000000011 ?00000000? ?021110101 20020?0000 0100100000 000?000020 0400000000 000000???1 2000002?10 0?11010000 0100001?01 1100011010 010100000? 1001100000 01??1????0 00?00?0??? ?000001??? 0?????0?0? ?????????? ?????????? ?????????? ???3000000 1000???0?? 2?0??????? ?????????0 ?0??0??0?0 006??00??? ??????1001 08?0?01??? ?????????? ?????????? ?????????? ?????????? ?????????? ?????????? ?????????? ?????????? ?????????? ?????????? ?010?????? ?????????? ?????????? ?????????? ?????????? ?????????? ?????????? ?????????? ?????????? ?????????? ?????????? ?????????? ?????????? ?????????? ?????????? ?????????? ?????????? ?????????? ?????????? ?????????? ?????????? ?????????? ?????????? ?????????? ?????????? ?????????? ???

*Stenocercus scapularis* 0000000011 11[01]0000000 [01]010010000 010[01]000010 1000001100 0111102100 0?0?000001 0110002000 0010000100 0100010000 0000000010 0000101110 1010000100 0000000001 0010011000 0000000011 ?00000000? ?001110101 20020?0000 0110100000 000?000020 0400000000 0000000001 2000002?00 0011010000 0100001[01]00 111?001000 001100?00? 1000100000 00?010?1?1 00?00?0??? ?00000???? 0???0?1?0? ?00??????? ?????????? ?????????? ???2000??? ?00????0?? ?????????? ???00????0 ?0??0??0?0 00???????? ?????????? ?????????? ?????????? ?????????? ?????????? ?????????? ?????????? ?????????? ?????1???? ?????????? ?????????? ?????????? ?????????? ?????????? ?????????? ?????????? ?????????? ?????????? ?????????? ?????????? ?????????? ?????????? ?????????? ?????????? ?????????? ?????????? ?????????? ?????????? ?????????? ?????????? ?????????? ?????????? ?????????? ?????????? ?????????? ?????????? ?????????? ?????????? ???

*Liolaemus alticolor* 0000000011 1100000100 101000?000 0101000000 1000001101 0111102100 0?0?000001 0110002200 0010001?10 0110010000 0001110010 0000101110 1010000000 0000000001 00000?1000 0000000011 ?00001000? ?0011??101 1002100000 0110100000 000?000020 0400000000 0000000001 2000002?11 0?01010000 0100001??0 0110001000 001100?00? 1000200000 00?110?1?1 00???????? ??0?00???? 0???0?0?0? ?00??????? ???????0?? ?????????? ???200000? ?00????0?? ?????????? ???00????0 ?0??0??0?0 00???????? ?????????? ?????????? ?????????? ?????????? ?????????? ?????????? ?????????? ?????????? ?????????? ?????????? ?????????? ?0000????? ?????????? ?????????? ?????????? ?????????? ?????????? ?????????? ?????????? ?????????? ?????????? ?????????? ?????????? ?????????? ?????????? ?????????? ?????????? ?????????? ?????????? ?????????? ?????????? ?????????? ?????????? ?????????? ?????????? ?????????? ?????????? ?????????? ???

*Geiseltaliellus maarius*  100??00001 11??000?00 ?0?????000 ??010?00?? 1000000100 00111?210? ????????01 011?1?2?00 0000000100 01?001?000 ?????????? 00????0??? ??????0??? ??00?????? ??1????0?? ????????11 ?0000?000? ?00011?100 00?210000? 01?0100??? ?00?00??20 0400000000 0?0000???1 20???????? ?????????? ?20000???1 010?0?0000 0?0100000? 100?0?0000 ?1???????? ?????????? ?????????? ?????????? ?????????? ?????????? ?????????? ???4?0?0?0 1?00?1?000 ?00??2?0?? ????0????0 ?0010??0?? ?????????? ?????????? ?????????? ?????????? ?????????? ?????????? ?????????? ?????????? ?????????? ?????1???? ?????????? ?????????? ?????????? ?????????? ?????????? ?????????? ?????????? ?????????? ?????????? ?????????? ?????????? ?????????? ?????????? ?????????? ?????????? ?????????? ?????????? ?????????? ?????????? ?????????? ?????????? ?????????? ?????????? ?????????? ?????????? ?????????? ?????????? ?????????? ?????????? ???

*Aciprion formosum* 000??00001 110?000000 003001?000 ??01000000 1000001100 010110210? ????000001 011?002?00 000000010? 1???011000 ?0???????? ??????11?? ??????0100 000?0000?1 0??0001010 1000????1? ??00?1000? ?00110?000 1002100011 0110100000 000?001020 0400000000 000000???? ?????????? ?????????? ?????????? ?????????? ?????????? ?????????? ?????????? ?????????? ?????????? ?????????? ?????????? ?????????? ?????????? ???3000??0 1?00?100?0 000??????? ???00????? ?0010??0?? ?????????? ?????????? ?????????? ?????????? ?????????? ?????????? ?????????? ?????????? ?????????? ?????????? ?????????? ?????????? ????0?000? ?010?000?? 00001??1?? ?0001?10?? ?010?00000 0?00000000 000???0100 000?1????? 0????????? ?????????? ????0????? ????0?0?10 ?000000?00 00???000?? 0000?????? 000?000??? ?0??00???? ???10?0001 0112200000 0000000000 00?0?????? ?????????? ?????????? ?????????? ?????????? ?????????? ??00?????? ???

*Babibasiliscus alxi* ??0?000011 ??0?????00 0?30?1?000 010100?000 10000001?? ?1?110?100 0?0?0000?? ?????????? ?????????? ?????????? 000?100010 0010101110 1010000010 0000?11001 0010001000 00000?0??1 ???0?0?00? ?000100100 0002??00?0 0110100000 000?000120 0400??0000 0??000???? 2????????? ?????????? ?????????? ?????????? ?????????? ?????????? ?????????? ?????????? ?????????? ?????????? ?????????? ?????????? ?????????? ???30?00?0 1?00111000 1?0??2???? ??0??0??00 ?0010????? ?????????? ??0??????? ?????????? ?????????? ?????????? ?????????? ?????????? ?????????? ?????????? ?????????? ?????????? ?????????? ??????000? 1??????0?? ?????????? ?????????? ?????00000 0?00000010 0?0??????? ?????1???? ?000?100?? ??????0?00 000000000? 00????0010 0000000?00 00?1?????? ?????????? 000?????10 0??1?????0 0?????00?1 0112200000 0000000??0 0000?????? ?????????? ?????????? ?????????? ?????????? ?????????? ?????????? ???

*Basiliscus basiliscus* 1000010001 1100000000 0030011000 0[01]01000000 1000000110 0011102100 0?0?000001 01121110?0 10?0000100 0???020000 0000010010 0010100110 1010000000 0000011000 0010001000 0000000010 ?00000000? ?000100100 2002110011 011010?00? ?00?000020 0400000000 0000000001 2100002?01 0?01010000 0200001001 0100000000 000100000? 1000000000 010100?1?0 00000?0??? ?0000?1??? 0?????1?1? ?????????? ???????0?? ?????????? ???20?0010 1010110000 000??2?001 ??0000?1?0 ?0010210?0 016??00202 110???0110 08?1??1000 ?00?0????? ?????????? ?????????? ?????????? ????00100? 0231000020 100??2???? ?????????? ?????????? 02000?0001 0010?000?? 00001??2?? ?00011000? 0010000000 0?00000010 0?00000000 00001100?0 0000010000 ?0?00?0?00 000000000? 0??0000010 0000000000 0001000000 000000100? 000?000010 0001001010 0010000001 0112000000 0000000000 0000010100 003?0?0001 1100110[01]00 11?011?000 1000?0?000 011001?000 0000?00010 0?0

*Basiliscus galeritus* 1000000001 1100000000 0030011000 0001000000 1000001100 0011102100 0?0?000001 01121112?0 00?0000101 0???020000 0000100010 0010100111 0010000000 0000011000 0010001000 0000000011 ?00000000? ?001100100 1002100011 0110100000 000?000020 0410000000 000000???1 2000002?10 0001010000 0000001001 0100000000 000100000? 100?000000 01??00?1?0 00000?0??? ?1000????? 0?????1?1? ?????????? ???????0?? ?????????? ???20?0010 1000110000 000??2?001 ??0000?1?0 ?0010??0?0 016??00??? ??0???0100 04?0??0??? ?????????? ?????????? ?????????? ?????????? ????00000? 010??????? ?????????? ?????????? ?????????? ?????????? ?????????? ?????????? ?????????? ?????????? ?????????? ?????????? ?????????? ?????????? ?????????? ?????????? ?????????? ?????????? ?????????? ?????????? ?????????? ?????????? ?????????? ?????????? ?????????? ?????????? ?????????? ?????????? ?????????? ?????????? ?????????? ?????????? ???

*Basiliscus plumifrons* 1000000001 1100000000 0030011000 0001000000 1000001100 0011102100 0?0?000001 01121112?0 00?0000101 0???020000 0000100010 0010100111 0010000000 0000011000 0010001000 0000000011 ?00000000? ?001100100 1002100011 0110100000 000?000020 0410000000 0000000001 2100002?10 0001010000 0000001001 0100000000 000100000? 100[01]000000 01??00?1?0 00000?0??? ?0000????? 0?????1?1? ?????????? ???????0?? ?????????? ???20?0010 1000110000 000??2?001 ??0000?1?0 ?0010210?0 016?600??? ??0???0100 0600101??? ?????????? ?????????? ?????????? ?????????? ????00000? 003??????? ?????2???? ?????????? ?????????? ?????????? ?????????? ?????????? ?????????? ?????????? ?????????? ?????????? ?????????? ?????????? ?????????? ?????????? ?????????? ?????????? ?????????? ?????????? ?????????? ?????????? ?????????? ?????????? ?????????? ?????????? ?????????? ?????????? ?????????? ?????????? ?????????? ?????????? ???

*Basiliscus vittatus* 1000000001 1100000000 [01]031[01]11000 0001000000 11000001[01]0 0001102100 0?0?000001 01121112?0 00?0000101 0???020000 0000100010 0010101110 1010000000 0000011[01]01 0010001000 00000?0011 ?00000000? ?001100100 11021?0021 011010000? ?00?000020 0400000000 000000???1 2100002?01 0?01010000 000000?001 0100000000 000100000? 100[01]000000 01???????? ?????????? ?????????? 0?????1?1? ?????????? ???????0?? ?????????? ???20?0110 2000210000 000??1?001 ??0000?1?0 ?0010[23]00?0 016??00??? ??0???0110 00?1??1??? ?????????? ?????????? ?????????? ?????????? ????00100? 023??????? ??????02?? ??00011100 0000010000 0????????? ?????????? ?????????? ?????????? ?????????? ?????????? ?????????? ?????????? ?????????? ?????????? ?????????? ?????????? ?????????? ?????????? ?????????? ?????????? ?????????? ?????????? ?????????? ?????????? ?????????? ?????????? ?????????? ?????????? ?????????? ?????????? ?????????? ???

*Corytophanes cristatus* 0000000011 1110000000 10?1011000 0101000100 1000000111 0011102100 0?0?100001 00121110?0 01?0000110 0???020000 0000100010 0010100110 1011000000 00000001?1 0010001000 0000000011 ?00000000? ?000100100 20320?0000 011010?000 000?000020 0400000000 0000000001 2000002?10 0001010000 0200000000 0100000000 000100000? 1001100000 01?100???0 00100?0??? ?0000????? 0?????1?1? ?????????? ???????0?? ?????????? ???30?0010 1020101000 ?00??2?012 ??0000?1?0 ?0010210?0 016??01??? ??0???0001 08?0?01000 ?0010????0 00[12]01110?? ?????????? ?????????? ???000100? 0131000020 100??2???? ?????????? ?????????? 02000?000? ?010?000?? 00001??2?? ?00011000? 0000000000 0?00000010 0?00000000 00001100?0 0000010000 ?0?0000?00 000000000? 0??0000010 0000000000 0001000000 000000100? 000?000010 0001001010 0010000001 0112100000 0000000000 0000010110 004?0?0000 1200010000 11?0111000 1000101000 0110010000 0000?00010 0?0

*Corytophanes hernandezi* 0000000011 1100000000 10?1011000 0101000100 1000000111 0011102100 0?0?000001 00121110?0 01?0000110 0???020000 0000100010 0010100110 1010000000 00000001?1 0010001000 0000000011 ?00000000? ?000100100 20320?0000 011010?000 000?000020 0400000000 0000000001 2000002?10 0001010000 0200000000 0100000000 000100000? 1001100000 010100?1?0 00100?0??? ?0000?1??? 0?????1?1? ?01??????? ???????0?? ?????????? ???30?0010 10[12]0111000 ?00??2?012 ??0000?1?0 ?0010210?0 016??00??? ??????1101 ?0?0??0??? ?????????? ?????????? ?????????? ?????????? ????00100? 0?3??????? ?????????? ?????????? ?????????? ?????????? ?????????? ?????????? ?????????? ?????????? ?????????? ?????????? ?????????? ?????????? ?????????? ?????????? ?????????? ?????????? ?????????? ?????????? ?????????? ?????????? ?????????? ?????????? ?????????? ?????????? ?????????? ?????????? ?????????? ?????????? ?????????? ?????????? ???

*Corytophanes percarinatus* 0000000011 1100000000 10?1011000 0101000100 1000000111 0011102100 0?0?100001 00121110?0 01?0000110 0???020000 0000100010 0010100110 1010000000 00000001?1 0010001000 0000000011 ?00000000? ?000100100 20320?0000 011010?000 000?000020 0400000000 0000000001 2000002?10 0001010000 0200000000 0100000000 000100000? 1001100000 01?100???0 00100?0??? ?0000????? 0?????1?1? ?????????? ???????0?? ?????????? ???30?0010 1020??10?0 ?00?????12 ??0??0?1?? ??010??0?? ?16??0???? ??????0101 08?0??1??? ?????????? ?????????? ?????????? ?????????? ????00100? ?1???????? ?????????? ?????????? ?????????? ?????????? ?????????? ?????????? ?????????? ?????????? ?????????? ?????????? ?????????? ?????????? ?????????? ?????????? ?????????? ?????????? ?????????? ?????????? ?????????? ?????????? ?????????? ?????????? ?????????? ?????????? ?????????? ?????????? ?????????? ?????????? ?????????? ?????????? ???

*Laemanctus longipes* 1000010011 1100000000 0030111000 0101000000 1000000111 0111102100 0?0?000001 01121022?0 00?0000110 0110010000 0000100010 0010101110 1010000100 0000011001 0010001000 00000?0011 ?10000000? ?001100100 00?2120000 011010?000 000?000120 0400000000 0000000001 2000002??? 0001010000 020000???1 010?00?000 00??00000? 1000???000 110??0?1?0 00100?0??? ?1000????? 0?????1?1? ?????????? ???????0?? ?????????? ???20?0010 1000?100?0 100?????01 ??0000?1?0 ??010??0?0 016??00??? ??????0001 00?0??0000 ?00?0????? ?????????? ?????????? ?????????? ????00100? 020??????? ?????1???? ?????????? ?????????? ?????????? ?????????? ?????????? ?????????? ?????????? ?????????? ?????????? ?????????? ?????????? ?????????? ?????????? ?????????? ?????????? ?????????? ?????????? ?????????? ?????????? ?????????? ?????????? ?????????? ?????????? ?????????? ?????????? ?????????? ?????????? ?????????? ?????????? ???

*Laemanctus serratus* 1000010011 1100000000 0031111000 0101000000 1000000111 0111102100 0?0?000001 01121022?0 00?0000110 0110010000 0000100010 0010[01]01110 1010000100 0000011001 0010001000 00000?0011 ?10000000? ?001100100 00?2120000 011010?000 000?000120 0400000000 0000000001 2000002??? 0001010000 020000???1 010?00?000 00??00000? 1000???000 11???0?1?0 00100?0??? ?1000????? 0?????1?1? ?????????? ???????0?? ?????????? ???20?00?? ?00011200? ??0??2?001 ???000?1?0 ?0010210?0 016??00??? ??0???0001 08?0??1??? ?????????? ?????????? ?????????? ?????????? ????00100? 020??????? ?????????? ?????????? ?????????? ?????????? ?????????? ?????????? ?????????? ?????????? ?????????? ?????????? ?????????? ?????????? ?????????? ?????????? ?????????? ?????????? ?????????? ?????????? ?????????? ?????????? ?????????? ?????????? ?????????? ?????????? ?????????? ?????????? ?????????? ?????????? ?????????? ?????????? ???

*Suzanniwana patriciana* ?????????1 11?000???? ?????????? ???10????0 ????0?1100 0???1?200? 0?0?????11 0?1?1?2001 000?00???1 0??00??000 ?????????? ??????01?? ?????????? ?????????? ?????????? ?????????? ?????????? ???????10? 020????0?? ?????????? ?????????? 0400??0000 ????0????? ?????????? ?????????? ?????????? ?????????? ?????????? ?????????? ?????????? ?????????? ?????????? ?????????? ?????????? ?????????? ?????????? ???3?????? ?????????0 ?00??????1 ????0????? ???1?2???? ?????????? ?????????? ?????????? ?????????? ?????????? ?????????? ?????????? ?????????? ?????????? ?????1???? ?????????? ?????????? ?????????? ?????????? ?????????? ?????????? ?????????? ?????????? ?????????? ?????????? ?????????? ?????????? ?????????? ?????????? ?????????? ?????????? ?????????? ?????????? ?????????? ?????????? ?????????? ?????????? ?????????? ?????????? ?????????? ?????????? ?????????? ?????????? ?????????? ???

*Hoplocercus spinosus* 0000002001 1100000000 1011110000 0001000000 4001001100 0011102101 0?0?000001 0110002000 0010000101 0110010000 ?000100010 0010101111 0010000000 00000101?1 0010201000 1000100010 ?00000000? ?020110100 00[01]20?0010 0110100000 000?000120 0400000000 0000000001 2000002?11 0?01010000 0100000100 110000[01]000 001100000? 1001200000 00??00?1?0 00???????? ??0?00?000 0????????? ?????????? ?????????? ?????????? ???200001? ?00????0?0 ?????????? ???00????0 ?00?0??0?0 00???00??? ??????0000 08?1?01??? ?????????? ?????????? ?????????? ?????????? ????????0? 013??????? ??????02?? ??00011100 0000010000 0????????? ?????????? ?????????? ?????????? ?????????? ?????????? ?????????? ?????????? ?????????? ?????????? ?????????? ?????????? ?????????? ?????????? ?????????? ?????????? ?????????? ?????????? ?????????? ?????????? ?????????? ?????????? ?????????? ?????????? ?????????? ?????????? ?????????? ???

*Morunasaurus annularis* 0000002001 1100000000 003010?000 0001000000 1001001100 000110210? 0?0?100001 0110002?00 0100000101 0110001000 00??100010 0010101111 0010000000 00000?01?1 10100?1000 0000000011 ?00001000? ?02010?100 0002110000 011010000? ?00?000020 0400000000 000000???? ?????????? ?????????? ?????????? ?????????? ?????????? ?????????? ????00?1?0 00???????? ??0?00?000 0????????? ?????????? ?????????? ?????????? ???20001?? ?00????0?0 ?????????? ???00????0 ?00?0??0?? 00???????? ?????????? ?????????? ?????????? ?????????? ?????????? ?????????? ????????0? 010??????? ??????02?? ??00011100 0000010000 02000?0101 0010?000?? 00001??020 000011100? 0010000000 0?00000000 0?00000000 00001100?0 0000010000 ?0?00?0?00 000000000? 0??0000110 0001000000 0011000000 00000?100? 000?000010 0001001010 0011000001 1112200000 0000000000 00?0010110 003?0?0000 1110000100 11?0111000 1000000000 011001[01]000 0000?01010 0?0

*Enyalioides palpebralis* 0000002011 1100000000 10[01]000?000 0101000000 4001001100 0011102101 0?0?000001 0110002000 0000000101 0110010000 0000100010 0000101110 0010000000 00000101?1 0010001000 0000000011 200000000? ?000100100 00020?0000 0110100000 000?000020 0400000000 000000???1 2000002?10 0001010000 0100001100 11000[01]1000 001200000? 1001200000 01000001?0 00000?0??? ?0000?1000 00?00?0?0? ?01??????? ?????????? ?????????? ???2000??? ?00????0?0 ?????????? ???00????0 ?00?0??0?0 00???????? ????????01 11?00?0??? ?????????? ?????????? ?????????? ?????????? ????????0? 020??????? ?????????? ?????????? ?????????? ?2000?0001 0010?000?? 00??1??020 000011100? 0010000000 0?00000000 0?00000000 00001100?0 0000010000 ?0?00?0?00 000000000? 0??0000110 0000000000 0011000000 00000?100? 000?000010 0001011010 0011000001 0112200000 0000000000 0000010110 003?0?0000 1200010000 11?0111000 1000000000 0110011000 0001001010 0?0

*Oplurus cyclurus* 0010000111 1100000000 1030011000 0101000000 1000000101 0111102101 0?0?000001 0210002000 0110000100 1???010??? 0001100010 0010101110 1010010100 0000010000 0010001000 0000100011 ?00000000? ?030110101 00020?0001 0110100000 000?000020 0400000000 0000000001 20000?2?00 ??01?????? ?1???????? ??0???10?? ?0???????? ?????????? ???00011?1 00???????? ??0?0????? 0???0?0?0? ?11??????? ?????????? ?????????? ???10000?0 1000?1?0?0 200?????11 ??000??1?0 ?0110??0?? 00???1???? ??????0000 01000?1??? ?????????? ?????????? ?????????? ?????????? ????????0? 013??????? ?????????? ?????????? ?????????? ?00?0?0001 0010?000?? 00??1??1?? ?00011100? 0010000000 0?00000000 0000000000 00001100?0 0000010000 ?0?00?0?00 000000000? 0??0000010 0000000?10 0001000000 000000100? 000?000010 0001001000 0011000001 1212200000 0000000000 00?0010100 403?0?0000 12[01]0100000 11?0111000 10000010[01]0 011001[01]000 0000?00010 000

*Chalarodon madagascariensis* 0010000111 1100000000 1040110000 0101000000 1000000100 0111102101 0?0?000001 1110002000 00100001?0 1???010000 0000100010 0010101111 0010010100 0000000000 0010001000 00000?0011 ?00000000? ?001110100 20120?0000 0110100000 000?000021 0400000000 000000000? ????????1? ?????????? ?1???????? ??0???20?? ?0???????? ?????????? ???00011?0 00???????? ??0??????? 0???0?0?0? ?11??????? ?????????? ?????????? ???10?00?0 1000?0?0?0 ?00??????1 ??000????0 ?0010??0?? 00???1???? ??????0001 08?0?01??? ?????????? ?????????? ?????????? ?????????? ????????0? 013??????? ?????1???? ?????????? ?????????? ?1000?0001 0010?000?? 00??1??1?? ?00011100? 0010000000 0?00000000 0000000000 00001100?0 0000010000 ?0?00?0?00 000000000? 0??0000010 0000000?10 0001000000 000000100? 000?000010 000100101? 0011000001 1112200000 0000000000 00?0010110 003?0?0000 1200100000 11?0111000 1000001??? 0110010000 0000?00010 000

*Polrussia mongoliensis* 000????000 1???000?0? ?????0?010 ??110?0??? 10??00110? ?1111021?? 0?0??????1 ?01??????0 0000?00001 ????00?000 ????0????? ???0??01?? ?00??0???? ?????????? ?????????? ?????????? ?????0000? ?001100100 2??20?0000 011010?00? ?00??0??2? 0000000000 ???000???? ?????????? ?????????? ?????????? ?????????? ?????????? ?????????? ?????????? ?????????? ?????????? ?????????? ?????????? ?????????? ?????????? ???800?0?? ??0??????0 ?0???????? ???00????? ????0????? ?????????? ?????????? ?????????? ?????????? ?????????? ?????????? ?????????? ?????????? ?????????? ?????????? ?????????? ?????????? ?????????? ?0???000?? 0000?????? ?????????? ?010?00000 ??000?0?00 0?0000???? ????1?0??? ?????????? ?????????? ?????????? ???0????10 00000001?0 00?1?0???? 00???????? ?????????? ?00??????? ????0?00?1 021?2?0010 0000000000 0000?????? ????0????? ?????????? ?????????? ?????????? ?????????? ?????????? ???

*Brachylophus fasciatus* 10[01]00[01]2011 1100000000 0030010000 00010000[01]0 1000001[01]00 0001101101 0?0?000011 0011002000 0010000101 010[01]010001 0001000010 0010101110 1000010000 0000000000 0010001000 0000000011 ?00000000? 0000100100 2021100000 0110101000 000?000020 0400000000 0000000001 2101202?01 0?01010000 0200000100 0100010000 000000000? 1000100000 01?100?1?0 00000?0??? ?0000?1??? 0?????0?0? ?00??????? ?????????? ????1?0??? ???90?0000 1000?000?0 100??????2 ??000??0?0 ?00[12]0210?0 006?600??? ?????????? ?????????? ?????????? ?????????? ?????????? ?????????? ?????????? ???1110010 000112???? ?????????? ?????????? ?0000?0001 1010?000?? 00??1??020 000011100? 0010000000 0?00010000 0000000000 00011100?0 0000010000 ?0?00?0?00 000010000? 0??0000010 0000000?10 0001000000 00000?100? 000?000010 000101[01]010 0011000001 0111200010 0000000000 0000010110 404?0?0000 1200000000 11?0111000 100000???? 011001?000 0000?00010 0?0

*Armandisaurus explorator* 100??00011 110?000000 0?3?01?000 ??01?00?10 1000001100 011110210? ????0??001 0110001?00 0010?00100 0110010001 0???1????? ?????0121? ?00?0?0??? ?????????? ??1???10?0 0???????1? ?0???00??? ?0001?0000 20?1??0000 0100?0?00? ?00?000020 0400000000 ?00000???1 20????2??? ?????????? ?????????? ?????????? ?????????? ?????????? ?????????? ?????????? ?????????? ?????????? ?????????? ?????????? ?????????? ???30?00?0 1?00???0?0 100?????01 ?????????0 ??010200?? ?????????? ?????????? ?????????? ?????????? ?????????? ?????????? ?????????? ?????????? ?????????? ?????????? ?????????? ?????????? ?1000?0001 ?010?000?? 00??1??020 0000??10?? ?010000000 0?00010000 0?00000000 00011100?? 0000?10?00 ?0?00?0??0 0?00?0000? 0??00000?0 0000000?10 0001000000 00000????? 0001?????? ?0?1000??? ?0?10000?? 0111200000 ??00000000 00?0?????? ?????????? ?????????? ?????????? ?????????? ?????????? ?????????? ???

*Dipsosaurus dorsalis* 0000000011 1100000000 0031010000 0101000000 1000001100 0111102100 0?0?000001 0110001000 0010000100 0110010001 0001100010 0010101[12]10 0000010100 0000000000 0010001000 0000000010 200000000? ?000110100 2001110000 0100101000 000?000020 0400000000 0000000001 2100202?01 0011010000 1000000000 0100010000 001100000? 1000100000 01?100?1?0 00000?0??? ?0000?1??? 0?????0?0? ?00??????? ??????[01]??? ?????????? ???30?0000 1000?010?0 100?????02 ??00???0?0 ?0020200?0 006?600202 11???????? ?????????? ?????????? ?????????? ?????????? ?????????? ?????????? ?????????? ???211???? ?????????? ?????????? ?[12]000?0001 1010?000?? 00??1??020 000011100? 0010000000 ??00010000 0?00000000 00011100?0 0000010000 ?0?00?0??0 000010000? 0??0000010 0001000?10 0001000000 000000100? 000?000010 0001000010 0011000001 1111200000 0000000000 00?0010110 004?0?0000 1200000100 11?0111000 100000111? 0110011000 0000?00010 0?0

*Sauromalus ater* 100000?000 1100000100 1030010000 0001000000 1000001100 0111102100 0?0?000011 011100[12]000 0010000111 0110010000 0001100010 0000101[12]10 0000010100 0000010000 00[01]0001000 0000000010 ?00000000? ?000100100 2022120001 0100100000 ?00?000120 0400000000 0000000001 2000202?00 0011010000 1000001000 0110010000 001000000? 1001200000 01?100?1?1 00000?0??? ?1000?10?? 0?????0?0? ?00??????? ??????0??? ?????????? ???30?0000 1000?110?0 100?????02 ??000????0 ?0130??000 027?601??? ?????????? ?????????? ?????????? ?????????? ?????????? ?????????? ????????0? 003??????? ??????02?? ??00011100 0000010000 0[12]000?0001 1010?000?? 00??1??020 000011100? 0010000000 0?00000000 0?00000000 00011100?0 0000010000 ?0?00?0??0 000010000? 0??0000010 0001000?10 0001000000 000000100? 000?000010 0000001010 0011000011 0111200010 0000000000 00?0010110 403?0?0000 1200[01]10000 11?0111000 1000001??? 0110011000 0000?00010 0?0

*Ctenosaura pectinata* 1000000001 1100000000 1030011000 0001000000 1000001100 0101102000 0?0?000011 0111102000 0100000110 0110020000 0001100010 0000101110 10100[01]0100 0000010000 0000001000 00000?0010 ?00000000? ??01100000 20121[01]0001 010010000? ?00?000020 0400000000 0000000001 2101202?00 0011010000 1000000100 1110010000 000100000? 1000100000 01?100?1?0 00000?0??? ?0000?10?? 0?????0?0? ?????????? ?????????? ?????????? ???30?0[01]00 1000?010?0 [12]20??????2 ??000??1?0 ?0020??000 006?601??? ?????????? ?????????? ?????????? ?????????? ?????????? ?????????? ?????????? ?????????? ?????????? ?????????? ?????????? ?????????? ?????????? ?????????? ?????????? ?????????? ?????????? ?????????? ?????????? ?????????? ?????????? ?????????? ?????????? ?????????? ?????????? ?????????? ?????????? ?????????? ?????????? ?????????? ?????????? ?????????? ?????????? ?????????? ?????????? ?????????? ?????????? ?????????? ???

*Iguana iguana* 3000000011 1100000000 0030011000 00010000[01]0 1000001100 0111102100 0?0?000001 0211102000 0000000111 0110020000 0001100010 0010101010 1100010000 0000010000 00100?1000 0000000010 ?00000000? ?001100100 2012110001 010010000? ?00?000[01][12]0 0400000000 0000000001 2101202?10 0011010000 0000000100 1110010000 000100000? 1001100000 01?110?1?0 00000?0??? ?0000?10?? 0?????0?0? ?????????? ??????0011 1111?????? ???20?0000 1000?010?0 1210????00 ??000??1?0 ?004031000 006?601202 11???????? ???????001 1?000????0 01?01400?? ?????????? ?????????? ?????????? ???1010000 000112???? ?????????? ?????????? ?????????? ?????????? ?????????? ?????????? ?????????? ?????????? ?????????? ?????????? ?????????? ?????????? ?????????? ?????????? ?????????? ?????????? ?????????? ?????????? ?????????? ?????????? ?????????? ?????????? ?????????? ?????????? ?????????? ?????????? ?????????? ?????????? ?????????? ???

*Crotaphytus bicinctores* 0000000011 1100000000 0030010000 0101000010 1000000001 0111102100 0?0?000001 0112002000 0010000101 0100010000 0001100010 0000101[02]10 0010010000 0000010000 0000011000 0000000011 200000000? ?0[01]10?0100 2032110011 0110100000 000?000020 0400000000 0010000001 2000002?10 0011010000 0200000000 1110010000 001100000? 1000100000 010?0001?1 00000?0??? ?0000?1000 00?00?0?0? ?01??????? ?????????? ?????????? ???3000000 1000?110?0 2010????22 ??000????0 ?0010??0?0 006?61???? ?????????? ?????????? ?????????? ?????????? ?????????? ?????????? ????????0? 013??????? ?????????? ?????????? ?????????? ?????????? ?????????? ?????????? ?????????? ?????????? ?????????? ?????????? ?????????? ?????????? ?????????? ?????????? ?????????? ?????????? ?????????? ?????????? ?????????? ?????????? ?????????? ?????????? ?????????? ?????????? ?????????? ?????????? ?????????? ?????????? ?????????? ?????????? ???

*Crotaphytus collaris* 0000000111 1100000000 1030010000 0101000010 1000001001 0111102100 0?0?000001 0112002000 0010000101 0100010000 0001100010 0000101110 1000010000 0000010000 0000001000 0000000011 ?00000000? ?001110100 1032110001 011010?000 000?000020 0400000000 0010000001 2000002?10 0011010000 0200000?00 1110010000 010100000? 1000100000 01??00?1?1 00000?0??? ?0000?1000 0??0??0?0? ?01??????? ??????[02]??? ?????????? ???3000000 1000?110?0 100?????22 ??000??1?0 ?0010200?0 006?61?202 11???????? ?????????? ?????????? ?????????? ?????????? ?????????? ????????0? 0131110010 100211???? ?????????? ?????????? 0000000101 1010?000?? 00001??1?? ?00011000? 0010000000 0?00000010 0000001000 00001100?0 0000010000 ?0?00?0?00 000000000? 0??0000010 0000000010 0011000000 000000100? 0000000010 0001000010 0011002011 0112200000 0000100?00 0000010100 403?0?0000 1200010000 11?0111000 1000001000 0110011000 0000?00010 000

*Gambelia wislizenii* 100000?000 1100000000 003[01]00?010 0101000010 1000001000 0111102100 0?0?000001 0112002000 0010000101 0100010000 0001100010 0010101110 1000010000 0000010000 0020001000 0000000011 ?00000000? ?001110100 2032110000 011010?000 000?000020 0400000000 0010000001 2000002?10 0011010000 0200000?01 0110010000 011100000? 1000100000 01??00?1?1 00000?0??? ?000001000 0?????0?0? ?????????? ??????2??? ?????????? ???3000000 1000?110?0 000?????12 ??000??0?0 ?0010200?0 006?61?202 11???????? ?????????? ?????????? ?????????? ?????????? ?????????? ????????0? 003??????? ?????102?? ??00011100 0000010000 00000?0101 0010?????? 00001??1?? ?00011000? 0010000000 0?00000010 0?00001000 00001100?0 0000010000 ?0?00?0?00 000000000? 0??0000010 0000000010 0001000000 000000100? 000?000010 0000000010 0011000011 011?200000 0000100000 0000010100 403?0?0000 1200110000 11?0111000 1000001000 0110011000 0000?00010 000

*Tropidurus torquatus* 000000?000 1100000000 103000?000 0101000000 1000001100 0001102100 0?0?000011 0112002110 0010020110 0110010000 00?0000010 0000100110 1010000?00 0?0010?1?1 000?001000 00?00???11 ?00000000? ?001??0100 20?20?0000 011010000? ?00?001020 0400000000 0000000001 2000002?11 0001010000 0100001000 1110011000 011100000? 1001200000 01??00?1?1 00100?0?00 01000????? ?????????? ?????????? ?????????? ?????????? ???20??000 1000011000 000?02?0?1 ??0??0?0?0 ?011021000 006?600??? ???0?00?00 0?001?1??? ?????????? ?????????? ?????????? ?????????? ????00000? 013??????? ?????1???? ?????????? ???0?????? ?00000000? 0010?????? 00??1??020 000011100? 1010000000 00?00000?? 0??000??00 0001110??? 000?010??? ???0?????? ?00?00??00 01???00010 0000?00??? 000100?0?? 000???100? 000?000010 ??0?0??01? ??0100?0?1 121?20000? 0000100000 00?0?10?1? ?03?0?0000 1010110000 11?0111000 10000????? 0110011000 000??0???? ???

*Microlophus albemarlensis* 000000?000 1100000000 101000?000 0101000000 1000000100 0101102100 0?0?000001 0110002000 0020000110 0110010000 0001100010 0000100110 1000000100 00000001?1 001?001000 00001?0011 ?00000000? ?021110100 20120?0000 010010000? ?00?000020 0400000000 000000???1 2000002?10 0?01010000 01000010?0 1110011000 011100000? ???0100000 0???00?1?0 00100?0?00 01000?1??? 0?????1?0? ?????????? ?????????? ?????????? ???20?0??? ?00????0?? ?0???????? ???00????0 ?0?10??0?0 00???1???? ??????0?00 0??0??1??? ?????????? ?????????? ?????????? ?????????? ????00000? 013??????? ?????????? ?????????? ?????????? ?????????? ?????????? ?????????? ?????????? ?????????? ?????????? ?????????? ?????????? ?????????? ?????????? ?????????? ?????????? ?????????? ?????????? ?????????? ?????????? ?????????? ?????????? ?????????? ?????????? ?????????? ?????????? ?????????? ?????????? ?????????? ?????????? ?????????? ???

*Plica plica* 000000?000 1100000000 1031011000 0101000000 1000001110 0011102100 0?0?000011 1112002000 0010000110 0110010000 0001000010 0000100210 1010000000 00000101?1 0010001000 0000000011 ?00000000? ?030110001 20320?01?? ?11010?000 000?000020 0400000000 0010000001 2000002?11 0001010000 0100001000 0110011000 011100000? 1000200000 01??0????0 00?00?0??? ??000?1??? 0?????0?0? ?????????? ???????01? 1111010101 001?0?0000 100????0?? 200??????? ?????????0 ?0??0??0?0 006?600??? ?????????? ???????00? ??[12]00????0 01?01411?? ?????????? ?????????? ?????????? ?????????? ?????????? ?????????? ?????????? ?1000?000? 0010?????? 00?01??020 000011100? 0010000000 0?00000000 0?00000000 00001100?0 0000010000 ?0?00?0?00 000010000? 0??0000110 0000000000 000100?000 00000?100? 000?000010 0001001010 0011000?01 ?210200000 0000000000 00?0010110 103?0?0000 1200010000 11?0111000 1000101[01]00 0110011000 0000?00010 000

*Callisaurus draconoides* 001000?000 1100000000 1031010000 0101000010 1000001100 0111102000 0?0?010011 1112012010 0010000110 0110000000 0001100010 0010100210 1103000000 00000001?1 0010001000 0000000011 ?00000000? ?001110101 2032100001 010010?000 000?001000 0300000000 0000000001 2000102?01 0001010000 0100001?00 0100011000 011100100? 1000100000 01??0??1?1 00?00?0??? 0?00001??? 0?????0?0? ?00??????? ??????1??? ?????????? ???30?0000 1000101000 000?01?0?1 ??1????000 ?00?0200?0 005?601202 11???????? ?????????? ?????????? ?????????? ?????????? ?????????? ?????????? ?????????? ??????02?? ??00011100 0000010000 020?00000? ?010?????? 00?01??020 000011100? 0010000000 0??000000? 0??000?100 000011???? 000??10??? ?????????? ?00??0???? ??????0010 0000?000?0 000100?0?? 0000?????? 00??0??010 ???100?01? ??1?10?001 111?2?0000 0000000000 00?0?????? ??4?0?0000 1010100000 ???01?10?0 1?00?????? ???001?00? 0????0???? ???

*Petrosaurus mearnsi* 101000?000 1100000000 0031111000 0101000010 1000001000 0101102100 0?0?[01]10011 1112002200 0000000110 0100010000 0000100010 0010100210 1000000[01]00 00000101?1 0000001000 0000000011 ?00000000? ?001110000 2032100000 011010?000 000?001000 0400000000 0000000001 2000002?00 0?01010000 0100000?00 1100001000 011100100? 1000100000 01??0??1?1 00000?0??? 0?000?1??? 0?????0?0? ?????????? ?????????? ?????????? ???30?0100 100????0?? 2010?????? ?????????0 ?0??0??0?0 005??00??? ?????????? ?????????? ?????????? ?????????? ?????????? ?????????? ?????????? ?????????? ??????02?? ??00011100 0000010000 010?0?0101 0010?????? 00?01??020 000011100? 0010000000 0?00000000 0?00000100 00001100?0 0000010000 ?0?00?0?00 000000000? 0??0000010 0000000?00 000100?000 000000100? 000?000010 0001000010 1011000001 1212200000 0000000000 00?0010100 403?0?0000 1200010000 11?0111000 1000001000 0110010000 0000?00010 000

*Sceloporus olivaceus* 1010000011 1200000000 1030010000 0101000010 1000000000 0101102100 0?0?100001 0112002000 0020020111 0100010000 0000100010 0010100201 0000010100 0000010000 0010001000 0000000011 ?00000000? ?001110000 1012110010 0100100000 000?000020 0300000000 0000000001 2000102?11 0001010000 0100001?01 1110011000 011100000? 1000200000 01??1??1?1 00100?0??? 01000?1??? 0?????0?0? ?????????? ??????1??? ?????????? ???3000000 100?111000 201002?0?1 ??0????100 ?001021010 005?600??? ??????0?01 1??0??1??? ?????????? ?????????? ?????????? ?????????? ????00000? 023??????? ?????????? ?????????? ?????????? ?20000000? 0010?????? 00?0???020 0000111011 1010000000 ???000000? 0??0000100 0001110??? 000??1000? ?0???????? 000?00???0 0??0?00010 0000000?00 000100?0?? 0000??100? 000?0??010 ?00100?010 0?11000001 111?2?0000 0000000000 00?0?????? ??3?0?0000 10101100?? 11?0??1000 1000?????? 111001?000 00???????? ???

*Uta stansburiana*  1010000011 1100000000 0010111000 0101000010 1000001100 0101102100 0?0?000001 1112002200 0010020010 0110000000 0000100010 0010100211 0000010000 0000100000 00200?1000 00000?0011 ?000?00000 1001110000 1011120000 0110100000 000?000020 0300000000 0000000001 200??????? ?????????? ?????????? ?????????? ????00?00? ?????????? ????00?201 00100?0??? 01000????? 0????????? ?????????? ??????[02]??? ?????????? ???30000?0 1000?010?0 ?010?????1 ??1??????0 ?01?0200?0 00???1???? ?????????? ?????????? ?????????? ?????????? ?????????? ?????????? ?????????? ?????????? ??????02?? ??00011100 0000010000 00000?0001 0010?????? 00??1??020 000011100? 0010000000 0?00000000 0?00000100 00001100?0 0000010000 ?0?00?0?00 000000000? 0??0000010 0000000?00 000100?000 00000?100? 000?000010 0001000010 0011000001 1212200000 0000000000 00?0010100 403?0?0000 1200010000 11?0111000 1000001000 0110010000 0000?00010 000

*Urosaurus nigricaudus* 0010000011 1200000000 001000?000 0101000000 1000001100 0101102100 0?0?000001 101?002000 0000000000 1???010000 0001100010 0010100201 0000000000 00000001?1 0000001000 0000100011 ?00000000? ?030??0001 21?20?0??? ?11010?000 000?001000 0300000000 0000000001 2001?02?01 0001010000 0100001?00 11[01]0002000 011100100? 1000100000 01??0??1?1 00000?0??? 00000?1??? 0????????? ?????????? ?????????? ?????????? ???30?0000 100????0?? ?010?????? ?????????0 ????0??0?0 005?600??? ?????????? ?????????? ?????????? ?????????? ?????????? ?????????? ?????????? ?????????? ?????????? ?????????? ?????????? ?????????? ?????????? ?????????? ?????????? ?????????? ?????????? ?????????? ?????????? ?????????? ?????????? ?????????? ?????????? ?????????? ?????????? ?????????? ?????????? ?????????? ?????????? ?????????? ?????????? ?????????? ?????????? ?????????? ?????????? ?????????? ?????????? ?????????? ???

*Anqingosaurus brevicephalus* ??00?????? ?????????? ????????00 ???00????? ?????????? ????10200? ????0????1 0?1?0?3?00 100??1???? ?????????? ?????????? ?????????? ?????????? ?????????? ?????????? ????????0? ?2000????? ???????1?? ?????????? ?????0???? ?00??????? ????000??? ???000???? ?????????? ?????????? ?????????? ?????????? ?????????? ?????????? ?????????? ?????????? ?????????? ?????????? ?????????? ?????????? ?????????? ???80????? ??0??????0 ?0???2???? ?????????0 ????0????? ?????????? ?????????? ?????????? ?????????? ?????????? ?????????? ?????????? ?????????? ?????????? ?????????? ?????????? ?????????? ?????????? ?????????? ?????????? ?????????? ?????????? ?????????? ?????????? ?????????? ?????????? ?????????? ?????????? ?????????? ?????????? ?????????? ?????????? ?????????? ?????????? ?????????? ?????????? ?????????? ?????????? ?????????? ?????????? ?????????? ?????????? ?????????? ?????????? ???

**TEXT A in S1: COMPARATIVE MATERIAL**

Observations of the following specimens and studies were used for this study. Institutional abbreviations: AMNH,

Anguimorpha

*Xenosaurus grandis:* AMNH R 19380, AMNH R 98122; [1-5]

Gobiguania

*Anchaurosaurus gilmorei*: [6]

*Ctenomastax parva*: IGM 3/61; [7]

*Saichangurvel davidsonae*: IGM 3/858 (holotype); [7, 8]

*Temujinia ellisoni*: IGM 3/63 (holotype); [7]

*Zapsosaurus sceliphros*: IGM 3/71 (holotype); [7]

Chamaeleontiformes

*Agama agama*: AMNH R 74800, AMNH R 140545, AMNH R 140546, AMNH R 140548; [9]

*Amphibolurus muricatus*: AMNH R 120263

*Brookesia brygooi*: [10, 11]

*Brookesia superciliaris*: YPM R 11370, YPM R 16522, AMNH R 71484; [12]

*Calotes versicolor*: AMNH R 31011, AMNH R 56478

*Chamaeleo chamaeleon*: FMNH R 22385, FMNH R 31294; [13]

*Draco blanfordii*: UF 63233

*Hydrosaurus amboinensis*: AMNH R 140825, AMNH R 147633

*Hypsilurus papuensis*: AMNH R 92647

*Intellagama lesueurii*: AMNH R 8998, AMNH R 141146

*Leiolepis reevesii*: AMNH R 30728, AMNH R 30761

*Lyriocephalus scutatus*: AMNH R 102450

*Phrynocephalus maculatus*: AMNH R 88561

*Physignathus cocincinus*: FMNH R 255017; [14]

*Pogona barbata*: AMNH R 76196, AMNH R 76570

*Rhampholeon boulengeri*: AMNH R 39299

*Rieppeleon brachyurus*: [12, 13]

*Rieppeleon brachyurus*: [12, 13]

*Rhampholeon spectrum*: [12]

*Uromastyx aegyptia*: FMNH R 63961, FMNH R 22214, FMNH R 142494; [15]

Pleurodonta

*Aciprion formosum*: USNM 16566, YPM PU 10015; [16, 17]

*Afairiguana avius*: FMNH PR 2379 (holotype); [18]

*Anisolepis grilli*: AMNH R 120468

*Anolis carolinensis*: AMNH R 70089, AMNH R 70102, FMNH 229898; [11, 19, 20]*Diplolaemus darwinii*: UC MVZ 93036

*Anolis* sp. (amber): [21-23]

*Armandisaurus explorator*: [24]

*Babibasiliscus alxi*: UWBM 89090

*Basiliscus basiliscus*: AMNH R 57769, AMNH R 75615; [25, 26]

*Basiliscus galeritus*: AMNH R 81492; [25]

*Basiliscus plumifrons*: AMNH R 140793, AMNH R 147831; [25]

*Basiliscus vittatus*: AMNH R 17883, AMNH R 147829, AMNH R 147832, FMNH R 98361, FMNH R 98362, FMNH R 98363; [25]

*Brachylophus fasciatus*: AMNH R 17701, AMNH R 147839; [27]

*Callisaurus draconoides*: AMNH R 147840, AMNH R 147841, AMNH R 147846

*Chalarodon madagascariensis*: AMNH R 71461; Evans, 2008; [28]

*Corytophanes cristatus*: AMNH R 16390, FMNH 206165, UF 166339; [25]

*Corytophanes hernandesii*: AMNH R 147880; [25]

*Corytophanes percarinatus*: USNM 113171; [25] *Crotaphytus bicinctores*: AMNH R 108970, AMNH R 108972; [20, 29]

*Crotaphytus collaris*: AMNH R 2362, AMNH R 84489, AM*Deiropteryx occulta*: AMNH R 115547, AMNH R 147826

*Ctenosaura pectinata*: AMNH R 71837, AMNH R 75474, AMNH R 75523; [30]

*Deiropteryx vermiculata*: AMNH R 63062, AMNH 70093

*Dipsosaurus dorsalis*: AMNH R 77436, AMNH R 75797, AMNH R 154766, AMNH R 154778, AMNH R 154772, AMNH R 154765, AMNH R 154768, AMNH R 114714, AMNH R 75552, FMNH R 249785, FMNH R 249786; [31]

*Enyalioides palpebralis*: FMNH 40008

*Enyalius bilineatus*: AMNH R 148521

*Gambelia wislizenii*: AMNH R 147874, AMNH R 147875, UF 49499; [20, 29]

*Geiseltaliellus maarius*: [32-35]

*Hoplocercus spinosus*: AMNH R 89398, AMNH R 90384, AMNH R 90658, AMNH R 93807, UF 69436

*Iguana iguana*: AMNH R 74736, AMNH R 75522, AMNH R 75524, UF 23076, UF 55873; [36]

*Isodontosaurus gracilis*: AMNH FR 6647 (holotype), IGM 3/84, IGM 3/85, IGM 3/86, IGM 3/87, IGM 3/88, IGM 3/89, ZPAL MgR II/3; [7, 37]

*Laemanctus longipes*: FMNH R 213398, MCZ 29333, MCZ 33108; [25]

*Laemanctus serratus*: AMNH R 44982; [25]

*Leiocephalus carinatus*: AMNH R 57461, AMNH R 70575, FMNH R 22754

*Leiosaurus catamarcensis*: [38]

*Liolaemus alticolor*: AMNH R 77610

*Microlophus albemarlensis*: AMNH R 77624

*Morunasaurus annularis*: AMNH R 57178

*Oplurus cyclurus*: AMNH R 71462, AMNH R 138120; [39]

*Petrosaurus mearnsi*: AMNH R 60513, AMNH R 141107

*Phenacosaurus heterodermus*: AMNH R 44987

*Plica plica*: AMNH R 85313, AMNH R 141159

*Polrussia mongoliensis*: IGM 3/73; [7, 40]

*Polychrus femoralis*: FMNH 81405

*Polychrus marmoratus*: AMNH R 141130; [41]

*Sauromalus ater*: AMNH R 73359, AMNH R 74814, AMNH R 75606, AMNH R 73516, FMNH R 22248; [42, 43]*Stenocercus scapularis*: FMNH R 40612

*Sceloporus olivaceus*: AMNH R 93183, AMNH R 93186, AMNH R 93185

*Suzanniwana patriciana*: UCMP 167664 (holotype); [44]

*Tropidurus torquatus*: UF 99303

*Urosaurus nigricaudus*: AMNH R 141087

*Urostrophus vautieri*: REE 2507; [45]

*Uta stansburiana*: AMNH R 141083, AMNH R 154857, FMNH R 98463; [46, 47]

Priscagamidae

*Arretosaurus ornatus*: AMNH FR 6706 (holotype), AMNH FR 6708; [37]

*Flaviagama dzerzhinksii*: [48]

*Gladidenagama semiplana*: [49]

*Mimeosaurus crassus*: AMNH FR 6655, IGM 3/74, IGM 3/75, IGM 3/76; [7, 37, 50]

*Phyrnosomimus asper*: IGM 3/81; [7, 49]

*Priscagama gobiensis*: ZPAL MgR III/32 (holotype), IGM 3/77, IGM 3/78, IGM 3/79; [7, 49]

**TEXT B in S1: Descriptions of Added Morphological Characters**

Several recent phylogenetic analyses have used morphology to address the interrelationships of Squamata or squamate subsets. Among these are several from Conrad and Conrad with co-authors [51-53]. The latest of these analyses included 493 characters, of which 490 were used for phylogenetic reconstruction. Here, 310 characters are added to that data matrix, most of which taken from or adapted from a recent, large-scale squamate analysis by Gauthier et al. [11]. Descriptions of these characters as used in the current analysis are provided below. Note that characters 236, 242, and 364 were not used in the analysis. The first two of those characters were replaced by subsequent characters, as described previously [52, 53], and the last is a biogeography character that was not used in any of the analyses from which the current data matrix is derived [see [51-53]].

Molecular data are those from Vieira et al. [54].

494. Nasal capsule, fenestra septi nasi [32, 55]:

0. absent

1. present

495. Nasal capsule, fenestra septi nasi [32, 55]:

0. slit-like

1. large, extending for half-or more of the septum nasi length

496. Nasal capsule, fenestra superior nasi [32]:

0. perforates tectum nasi

1. tectum nasi whole

497. Nasal capsule, nasal process [32]:

0. present

1. absent

498. Nasal capsule, M. nasalis externus [32]:

0. absent

1. present

499. Nasal capsule, lateral nasal fissure [32]:

0. fully developed

1. partially closed

2. absent

500. Nasal capsule, nasal conch [32]:

0. present

1. absent

501. Nasal capsule, cavum conchale [32]:

0. present

1. absent

502. Nasal capsule, sphenethmoid commisure [32]:

0. fully connected with the interorbital cartilages

1. poorly developed

503. Nasal capsule, conch of Jacobson's organ [32]:

0. present

1. absent

504. Nasal capsule, nasal vestibule [32]:

0. short, with rostral entrance into cavum nasi

1. intermediate in length, with entrance into cavum nasi just posterior to Jacobson's organ

2. elongate, subequal to the nasal capsule or longer

505. Nasal capsule shape [32]:

0. main body straight

1. S-shaped in horizontal plane

2. U-shaped in vertical plane

506. Dentition, posterior marginal tooth crown widths [32, 56]:

0. tapered

1. parallel-sided

2. distinctly flared

507. Glossus, tongue papillae [57]:

0. present

1. absent, tongue keratinized

508. Glossus, form of tongue papillae [57]:

0. filamentous

1. peg-like

2. conical

509. Glossus, squamos papillae form [57]:

0. smooth

1. flattened

2. serrated

3. with finger-like projections

510. Glossus, filamentous tongue papillae [57]:

0. distinct, without reticulation

1. with reticulation

511. Glossus, filamentous tongue papillae distal shape [57]:

0. symmetrical

1. asymmetrical, with apices (pointed]

512. Glossus, reticulate filamentous papillae on hind tongue [57]:

0. absent

1. present

513. Glossus, lingual scales [57]:

0. absent

1. present

514. Glossus, foretongue plicae [57]:

0. absent

1. present

515. Glossus, hind tongue plicae [57]:

0. absent

1. present

516. Glossus, M. genioglossus buccal floor insertion [57]:

0. absent

1. present

517. Glossus, M. genioglossus division [57]:

0. absent, undivided

1. present, divided into medial and lateral parts

518. Glossus, M. transversalis and M. verticalis [57]:

0. M. transversalis insert onto the transverse septum

1. M. transversalis is contiguous with the M. verticalis, completely surrounding the hyoglossus bundles

519. Glossus, M. hyoglossus bundle number [57]:

0. two

1. multiple subdivisions

520. Glossus, foretongue muscular elaboration [57]:

0. absent, foretongue similar in breadth to the hyoglossal bundles

1. muscular elaboration such that the foretongue is broader than the hyoglossal bundles

521. Glossus, foretongue muscular elaboration bundles [57]:

0. bundles absent

1. bundles present

522. Glossus, M. verticalis fibers [57]:

0. no crossover of fibers from right to left

1. with crossing fibers

523. Glossus, laryngohyoid ligament division [57]:

0. undivided

1. bifurcated

524. Hyoid, entohyal connection with the body [57]:

0. fused

1. ligamentous

525. Glossus, foretongue epithelial cells [57]:

0. present

1. absent

526. Glossus, lingual mucocytes [57]:

0. muco-serous

1. sero-mucus

527. Glossus, foretongue tapering [57]:

0. absent

1. present

528. Glossus, foretongue barbing [57]:

0. absent

1. present

529. Glossus, foretongue tines [57]:

0. broadly attenuated

1. sharply attenuated

530. Glossus, transverse infralingual plicae [57]:

0. absent

1. present

531. Glossus, posterior limbs of tongue Glossus, transverse infralingual plicae [57]:

0. present

1. absent

532. Premaxilla, anterior ethmoidal foramen(-ina] exit [modified from [11]]:

0. premaxilla without notch or foramina

1. premaxilla forms medial margin of the ethmoidal foramen or notch

2. premaxilla with ethmoidal foramen

533. Maxilla, anterior ethmoidal foramen(-ina] exit [modified from [11]]:

0. maxilla without notch or foramen

1. maxilla with contributes lateral border

2. maxilla with ethmoidal foramen

534. Premaxilla, ventral ethmoidal foramina [11]:

0. small

1. large

2. absent

535. Premaxilla, dorsoventrally bifid internasal process [11]:

0. absent

1. present

536. Premaxilla, conspicuous vertical margin on the maxillary process (anterior narial wall] [11]:

0. absent

1. present

537. Nasal, descending lamina [11]:

0. absent

1. present, extending below the nasal-maxilla and/or nasal-frontal sutures

538. Nasal, supranasal process well developed and with lateral offset [11]:

0. present

1. absent

539. Nasal, contact with the maxilla [11]:

Modified and their state 3 synonymized with state 0.

0. maxilla primarily overlaps the nasal

1. nasal overlaps the maxilla

2. abutting contact

540. Nasal, posteroventral contact at midline ventral to the premaxillary nasal process [11]:

0. present

1. absent

541. Nasals, contact dorsal to the premaxillary nasal process [11]:

0. absent

1. present

2. present, depth of internasal contact subequal to the depth of the premaxillary nasal process

542. Nasals, length relative to frontals [11]:

0. nasals shorter

1. nasals longer

543. Nasal, anterior extent relative to maxillary tooth row [11]:

0. nasal extends to a level anterior to the anteriormost maxillary tooth

1. anterior margin of the nasal lies behind the level of the anterior tip of the maxillary tooth row

544. Nasal, contact between frontal lamina of nasal and frontal [11]:

Unordered.

0. present and broad

1. narrow, point contact

2. absent

545. Frontal(s], subolfactory process fusion and obliteration of midline suture [modified from [11]]:

0. absent

1. present

546. Frontal, contact between the subolfactory processes and the parabasisphenoid [11]:

0. absent

1. present

547. Frontal, accessory descending lamina from the subolfactory tube [11]:

0. absent

1. present

2. present, clasping the parabasisphenoid

548. Frontal, prefrontal lamina of the subolfactory process [11]:

0. absent

1. knob-like

2. well-developed, ventrally directed lamina

3. well-developed and ventrolaterally directed

549. Frontal, subolfactory ramus of the medial pillar process [11]:

0. straight

1. forms a thickened anterolaterally projecting flange

550. Frontal, fusion of medial pillar with the subolfactory process [modified from [11]]:

0. absent

1. present

551. Frontal, contact with the prefrontal [modified from [11]]:

0. frontal lies primarily medial to the prefrontal

1. frontal broadly overlaps the prefrontal dorsally

2. prefrontal broadly overlaps the frontal posterodorsally

552. Frontal, supraorbital shelf [modified [11]]:

0. absent

1. present

553. Frontal, prefrontal process extending into the prefrontal pocket [11]:

0. absent

1. present

554. Frontoparietal contact, frontal convexity fitting into parietal concativity in sagittal plane, at the midline [11]:

0. absent

1. present, rounded

2. present, attenuated

555. Frontoparietal contact, arrangement of bones at the lateral part of the contact [11]:

0. frontal overlaps parietal

1. parietal overlaps frontal

2. abutting

3. rounded convexity of frontal invades anterior margin of parietal

556. Frontoparietal contact, shape of suture in medial orbital wall [11]:

0. strongly posteroventrally oriented

1. mostly vertical

2. posterodorsally oriented

557. Frontal, suboptic shelf (posteroventral shelf participating in the floor of the optical foramen/canal] [11]:

0. absent

1. present, no parietal contact

2. present, parietal contact

558. Postfrontal, fusion [modified from [11]]:

0. free

1. extra state

2. fused to postorbital

3. fused to frontal

559. Postfrontal, parietal process [modified from [11]]:

0. subequal in length to the frontal process

1. less than 60 percent of frontal process

2. absent as a distinct process

560. Postfrontal, lateral/ventral tip shape [modified from [11]]:

0. single, does not bifurcate

1. bifid

561. Postfrontal, relationship with jugal [11]:

Unordered.

0. postfrontal broadly separated from jugal

1. postfrontal approaches the jugal, separated by a space that is less than anteroposterior breadth of the jugal

2. in contact

562. Postorbital, medial part on the dorsal skull roof (modified from [11]]:

0. abutting contact lacking anteromedial process

1. elongate anteromedial process

563. Postorbital, posteromedial process offset from the anteromedial one [11]:

0. present

1. absent

564. Postorbital, dorsomedial flange restricting the upper temporal fenestra [modified from [11]]:

Unordered.

0. absent

1. present, roofs the anterior part of the supratemporal fenestra

2. present, roofing the anterior and posterior part of the supratemporal fenestra (fenestra closed]

565. Postorbital, nature of the squamosal contact [11]:

0. postorbital lies on the lateral surface of the squamosal, fitting into a V-shaped recess in the squamosal

1. postorbital lies dorsomedial to the squamosal

2. postorbital lies dorsal to the squamosal

3. postorbital lies primarily dorsolateral to the squamosal

4. squamosal lies in a trough beneath the postorbital

566. Postorbital, suture with the squamosal [11]:

0. suture is firm and the gap between the bones is no wider than those among the surrounding elements

1. suture is loose, a signficiant sutural gap exists and is wider than that between the postorbital and postfrontal and/or the postorbital and jugal]

567. Postorbital, contact with skull roofing bones [modified from [11]]:

0. sutural

1. non-sutural, abutting

568. Parietal, prominent V-shaped and flat anterior process [11]:

0. absent

1. present

569. Parietal, contact with braincase at midline [modified from [11]]:

0. absent (states 0 and 3 from Gauthier et al., 2012]

1. present (character states 1, 2, and 4]

570. Parietal, contact with braincase at midline [modified from [11]]:

0. parietal overlaps the supraoccipital at midline (states 1, 4]

1. parietal abuts the anterior surface of the supraoccipital (state 2]

571. Parietal, parasagittal contact with braincase [modified from [11]]:

0. absent (state 0]

1. present (states 1, 2, 3, 4]

572. Parietal, bifid supraoccipital process [11]:

0. absent

1. present

2. present and clasping the supraoccipital

573. Parietal, orientation of the supratemporal processes [11]:

0. mediolaterally oriented

1. posterolaterally oriented

2. anteroposteriorly oriented

574. Parietal, shape of the tab-like decensus parietalis [modified from [11]]:

0. absent

1. short, less than twice as deep as the anteroposterior length

2. elongate, more than twice as deep as the anteroposterior length

575. Parietal, extent of parietal-prootic contact [11]:

0. absent (scored as “-”]

1. contact between the apex of the decensus parietalis and the apex of the alar process

2. extensive contact wherein the parietal has extensive dorsal overlap of the prootic along its length

3. discrete but extensive lateral overlap of the prootic alar process laterally by the parietal

576. Maxilla, height of nasal process [11]:

0. tall, extends onto the skull roof

1. short, extending significantly dorsally along the snout, but not reaching the skull roof

2. absent (delete]

577. Maxilla, suture with the palatine [11]:

0. strongly sutured

1. loosely ligamentous connection

2. maxilla free of palatine

578. Maxilla, posterior extent of the suborbital ramus [modified from [11]]:

0. with at least some suborbital component

1. extends to posterior quarter of the orbit

2. extends at least to a level near the posterior orbital margin

3. extends to a level beyond the posterior border of the orbit

579. Jugal, suborbital depth [11]:

0. suborbital ramus of the jugal not much deeper dorsoventrally below mid-orbit than postorbital ramus anteroposterior width

1. jugal very deep ventral to the orbit, 200 percent or more of the anteroposterior width of the postorbital ramus of the jugal

580. Maxilla, intramaxillary joint [11]:

0. absent

1. present

581. Prefrontal, orbitonasal margin [modified from [11]]:

Unordered.

0. medial margin of the prefrontal slopes ventrolaterally

1. medial margin of the prefrontal vertically oriented

2. medial margin of the prefrontal slopes ventromedially (234]

3. medial border of the prefrontal medially bowed

582. Prefrontal, ventromedial process on the orbitonasal margin [modified from [11]]:

0. absent

1. present

583. Prefrontal, nasolacrimal cornu [11]:

0. absent

1. present

584. Prefrontal, medial extent [11]:

0. across less than 50% of the frontal

1. extends more than 50% across the frontal

2. across more than 65% of the frontal

3. extends across more than 75% of frontal

4. extends across 85% or more of the frontal width

585. Prefrontal, anteroposterior length relative to dorsoventral depth [11]:

0. anteroposteriorly elongate (longer than tall]

1. short anteroposteriorly (taller than long]

586. Prefrontal, arching margin around the orbitonasal fenestra [11]:

0. absent, margin more or less linear

1. present, curved margin

587. Lacrimal, position relative to the lacrimal canal [11]:

0. forms the lateral wall from the maxilla ventrally to the prefrontal dorsally

1. reduced to floor of canal with little or no lateral exposure

2. lacrimal reduced ventrally and confined mostly to the dorsolateral margin of the lacrimal canal

3. lacrimal completely encircles the lacrimal canal

588. Prefrontal, contributions to the lacrimal canal [modified from [11]]:

0. prefrontal forms medial wall of canal

1. prefrontal contributes medial and lateral walls to the canal

2. prefrontal completely encloses the lacrimal canal and fused ventrally

589. Jugal, contact with the maxilla [modified from [11]]:

0. jugal with a rounded ventral margin fitting into a shallow groove--the groove is approximately as broad as deep

1. acute, ventrolateral maxillary process of the jugal fits into a deep groove on the maxilla--groove much deeper than wide

590. Jugal, V-shaped notch clasping the suborbital process of the maxilla [11]:

0. absent

1. present

591. Jugal, suborbital boss (Gauthier et al., 2012; character 151]:

0. absent

1. present

592. Jugal, orientation of the posteroventral process [11]:

0. posteriorly directed

1. posteroventrally directed

593. Jugal, medial ridge size and shape [11]:

0. weakly developed and lying directly lateral to the ectopterygoid

1. pronounced and lying posterolateral to the ectopterygoid

594. Squamosal, contact of temporal ramus and parietal [modified from [11]]:

0. ramus diverges from the parietal with only a short posterior contact

1. broad parietal contact with the parietal ramus convergent with the parietal

595. Squamosal, dorsomedial flange of the supratemporal ramus [modified from [11]]:

0. absent

1. present posteriorly

2. present and extends anteriorly to restrict the supratemporal fenestra

596. Squamosal, shape of the temporal ramus in cross-section [11]:

0. mediolaterally compressed

1. dorsoventrally depressed

597. Parietal, medial supratemporal notch receiving acute medial margin of the supratemporal [11]:

0. absent

1. present

598. Supratemporal, ventromedial contact on the parietal [modified from [11]]:

0. present

1. absent

599. Supratemporal, position relative to the parietal [modified from [11]]:

0. ventrolaterally

1. laterally

2. deleted state (-]

3. dorsally

600. Supratemporal, anterior terminus [11]:

0. posterior to the trigeminal notch/foramen

1. at level of or anterior to the trigeminal notch/foramen

601. Supratemporal, orientation [11]:

0. anteroposteriorly oriented

1. dorsoventrally oriented

602. Supratemporal, free posterior margin [modified from [11]]:

0. sutured to skull medially

1. free posteriorly, without medial suture

603. Supratemporal, completely hidden in dorsal view by the squamosal-parietal contact [11]:

0. absent

1. present

604. Supratemporal, exposure on the supratemporal process [modified from [11]]:

0. extends dorsally for less than 2/5 of the postermedial surface of the parietal supratemporal process

1. extends dorsally for more than 2/5 of the postermedial surface of the parietal supratemporal process

605. Quadrate, suprastapedial ramus of the posterior crest [modified from [11]]:

0. absent

1. present

606. Quadrate, contact with the pterygoid [modified from [11]]:

0. present, bone-on-bone (01]

1. absent, ligamentous connection (2]

607. Quadrate, dorsomedial process abutting braincase [11]:

0. absent

1. present

608. Quadrate, stylohyal process on medial quadrate surface [modified from [11]]:

0. absent (0]

1. present (1,2]

609. Quadrate, stylohyal process shape [modified from [11]]:

0. disc-shaped

1. cylindrical ridge/finger-like process

610. Stapes, canal [11]:

0. absent

1. present

611. Braincase, orientation of fenestra ovalis [11]:

0. laterally

1. anterolaterally

2. ventrolaterally

3. posterolaterally

612. Septomaxilla, lateral edge contact with maxilla [modified from [11]]:

0. present

1. absent

613. Septomaxilla, lateral edge contact with nasal [modified from [11]]:

0. absent

1. present

614. Septomaxilla, lateral edge contact with prefrontal [modified from [11]]:

0. absent

1. present

615. Septomaxilla, suture with maxilla [11]:

0. sutured

1. mobile, no suture

616. Septomaxilla, dorsal roof over the vomeronasal compartment [11]:

0. absent

1. present

617. Septomaxilla, shape of the dorsal surface [11]:

0. dorsally convex

1. dorsally concave

618. Septomaxilla, ventromedial processes [11]:

0. absent

1. present

619. Septomaxilla, dorsolateral flange [11]:

0. absent

1. present

2. present and elongate, reaching well above the medial roof

620. Septomaxilla, medial flange [11]:

0. absent

1. present

621. Septomaxilla, posterolateral process from the lateral ascending flange [11]:

0. absent

1. present

622. Septomaxilla, enclosure of the medial ethmoid nerve [11]:

0. absent

1. at least anteriorly

2. for at least 1/2 of the ethmoidal length

3. for the length of the ethmoid

623. Septomaxilla, medial fenestra of vomeronasal cupola [11]:

0. present

1. absent

624. Vomer, relative to the vomeronasal organ [11]:

0. vomer ventrally encapsulates vomeronasal organ

1. vomer encloses vomeronasal organ posteriorly and medially

2. with margins enxlosing the posterior wall sloping ventromedially

3. further expanded laterally to completely encapsulate the vomeronasal organ posteriorly

625. Vomer, passage of vomeronasal nerve [modified from [11]]:

0. vomer lies completely ventral to the passage of the nerve, without enclosing it

1. vomer dorsally grooved for passage of the vomeronasal nerve

2. vomeronasal nerve completely surrounded by the vomer (2, 3]

626. Vomer, vomeronasal nerve emargination of the vomer [modified from [11]]

0. single notch or foramen

1. sieve-like passage of the nerve

627. Vomer, extent of contact with palatine [modified from [11]]:

0. relatively short overlap of the vomer by the palatine, less than 1/2 of the snout depth

1. relatively long overlap of the vomer by the palatine, more than 1/2 of the snout depth

628. Vomer, transverse fenestra in septum [modified from [11]]:

0. absent

1. present

629. Vomer, vertical lamina [modified from [11]]:

0. absent

1. present

2. tall, with dorsomedial expansion of the flange

630. Vomer, posterodorsal margin with expanded hollow flange [11]:

0. absent

1. present

631. Vomer, transverse flange contacting the septomaxilla and forming the posterior wall of the vomeronasal space [11]:

0. absent

1. present

632. Vomer, contact with the frontal subolfactory process [11]:

0. absent

1. present

633. Palate, descending tubercle or ridge [11]:

0. absent

1. present as an anteroposteriorly short, but dorsoventrally deep, process

2. present as an elongate ridge on the vomer and sometimes the palatine

634. Vomer, medial palatal foramen(-ina] [11]:

0. paired

1. single (at the midline]

635. Palatine, contact with the jugal/lacrimal [modified from [11]]:

0. absent

1. present

636. Palatines, cross-sectional shape [11]:

0. mostly flat

1. U-shaped

2. inverted U-shaped

637. Vomer, nature of the palatine contact [modified from [11]]:

0. elongate overlap

1. reduced overlap; presumably a loose connection

2. no bone-on-bone contact

3. laterally abutting contact (added state]

638. Palatine, vomerine process articulation with vomer [modified from [11]]:

0. relatively flat

1. elongate, tongue-in-groove, articulation

639. Palatine, anterolateral vomerine buttress [11]:

0. absent

1. present

640. Palatine, invasion of the midline suture of the vomer [11]:

0. present

1. absent

641. Palatine, anteroventral palatine projection [11]:

0. absent

1. present

642. Palatine, ventromedial ramus from the maxillary process [modified from [11]]:

0. present

1. absent

643. Palatine, orientation of the ventromedial process [modified from [11]]:

0. ventrally

1. ventromedially oriented

644. Palatine, teeth anterior to the level of the maxillary process [modified from [11]]:

0. absent

1. present

645. Palatine, anterior division of the infraorbital canal [11]:

0. absent, foramen single anteriorly

1. canal double anteriorly, with medial palatine ramus small and lateral large

646. Palatine, contact between palatine choanal process and the vomer [11]:

0. strong articular contact

1. without articular facet on palatine

647. Palatine, main body (dorsomedial part; choanal process of the palatine according to Gauthier et al., 2012] [modified from [11]]:

Unordered

0. elongate

1. narrow, anteromedial, finger-like process

2. expressed only as short lamina, without a vomerine contact

648. Palatine, lateral flange development restricting the suborbital fenestra [modified from [11]]:

0. absent

1. present

649. Pterygoid, posterior extent [11]:

0. terminates anteriorly, not reaching beyond the midpoint between the basipterygoid processes and the spheno-basiocciptal suture

1. reaching beyond the level of the parabasisphenoid-basiocciptal suture

2. reaching to the level of the occipital condyle

3. extending well beyond the occipital condyle

650. Pterygoid, wrapping around the posteromedial/ventromedial part of the quadrate [modified from [11]]:

0. absent

1. present

651. Pterygoid, ventral flange [11]:

0. absent

1. present

652. Ectopterygoid [11]:

0. present

1. absent

653. Ectopterygoid, anteromedial flange expansion [modified from [11]]:

0. absent

1. present

654. Ectopterygoid, anterior separation from the palatine [modified from [11]]:

0. well separated

1. approaching or contacting

655. Ectopterygoid, contact with the maxilla [modified from [11]]:

0. ectopterygoid lies dorsally along the maxillary supradental shelf (character 274 state 1 and character 275 state 0]

1. ectopterygoid abuts the posteromedially maxillary corner

2. ectopterygoid with slot laterally clasping the maxilla

3. ectopterygoid overlapping the maxilla more ventrally than dorsally

4. interdigitating suture, with maxilla t least partly overlapping ectopterygoid dorsally

656. Ectopterygoid, anterior tip [11]:

0. unexpanded mediolaterally

1. expanded anteriorly

2. expanded anteriorly to more than three times the main shaft

657. Ectopterygoid, contact with maxilla [modified from [11]]:

0. present

1. absent

658. Maxilla, posterior point invades the palatine-ectopterygoid suture [modified from [11]]:

0. present

1. absent

659. Ectopterygoid, dorsal process [modified from [11]]:

0. present

1. absent

660. Ectopterygoid, contact with prefrontal [11]:

0. absent

1. present

661. Ectopterygoid, pronounced supra-ptergyoid process [modified from [11]]:

0. present

1. absent

662. Ectopterygoid, pterygoid process length [modified from [11]]:

0. short, overlap less than twice as long as the depth of the pterygoid

1. long, overlap more than twice as long as the depth of the pterygoid

663. Epipterygoid, contact with the parietal [11]:

0. absent

1. present

664. Epipterygoid, shape [11]:

0. anteroposteriorly elongate, mediolaterally flattened

1. columelliform

665. Supraoccipital [11]:

0. single

1. paired

666. Braincase, short posterolaterally-oriented nuchal crest [11]:

0. absent

1. present on the supraoccipital

2. present on the supraocciptial and the otoocciptal

667. Supraoccipital, overlap otooccipital at midline [modified from [11]]:

0. absent

1. present

668. Parietal, overlaps the level of the otooccipital at midline [modified from [11]]:

0. absent

1. present

669. Supraoccipital, contribution to the side wall of the neurocranium [11]:

0. present

1. absent

670. Epiotic foramen [11]:

0. absent

1. present

671. Prootic, crista prootica shape [modified from [11]]:

0. laterally smooth

1. laterally pointed, aliform

672. Otooccipital, crista tuberalis and crista prootica [11]:

0. separate

1. combined to surround the stapedial footplate and lateral aperture of the recessus scalae tympani

673. Otooccipital, crista interfenestralis [11]:

0. prominent

1. present, but without a strong distal keel

2. absent

674. Otooccipital, crista tuberalis development [11]:

0. well-developed (prominent]

1. present, but not extremely prominent

2. absent

675. Prootic, contributes to the margin of the medial aperture of the recessus scala tympani [11]:

0. absent

1. present

676. Opisthotic/otooccipital, contribution to the posterior auditory foramen [11]:

0. present

1. absent

677. Orbitosphenoid [11]:

question of homology; leave unordered

0. absent as an osteological element

1. present

678. Orbitosphenoid, orbitosphenoid shape [modified from [11]]:

0. large, tri-radiate

1. triangular, "reduced"

2. expanded plate anteroventrally (paired or fused; 01, character 318]

679. Orbitosphenoid, number [11]:

0. paired

1. fused

680. Prefrontal, contributes to the opening for the maxillary branch of the trigeminal nerve [11]:

0. absent

1. present

681. Parabasisphenoid, dorsum sellae [modified from [11]]:

0. well-differentiated by the presence of a crista sellaris

1. shallow depression

682. Parabasisphenoid, lateral walls of dorsum sella [modified from [11]]:

0. absent

1. present, lateral basisphenoid walls

683. Parabasisphenoid, extent of roofing of the dorsum sella by the crista sellaris [modified from [11]]:

0. narrow overlap of roofing

1. cup-like, at least subequal in height and anteroposterior depth

2. deeply cup-shaped, anteroposterior depth more than 1.5 times dorsoventral height

684. Parabasisphenoid, keel depth [modified from [11]]:

0. tubercle

1. "shallow"

2. "deep"

3. broad pedicel contiguous with the parasphenoid rostrum

685. Parasphenoid rostrum, attenuated dorsal ridge in cross-section [modified from [11]]:

0. absent

1. present

686. Parasphenoid rostrum, mediolateral constriction with narrow vertical lamina in cross-section [modified from [11]]:

0. absent

1. present

687. Parasphenoid rostrum, prominent ventrolateral alae that extend far beyond the level of the dorsal margin [modified from [11]]:

0. absent

1. present

688. Parabasisphenoid, cultriform process [modified from [11]]:

0. present, elongate

1. present

2. absent

689. Parietal, dorsal contribution to anterior opening of the Vidian canal [11]:

0. present

1. absent

690. Braincase, size of the posterior opening of the right Vidian canal relative to that of the left Vidian canal [modified from [58] and [11]]:

0. subequal in size

1. posterior aperture of the right Vidian canal larger

691. Trabeculae cranii [modified from [11]]:

0. tropibasic

1. platybasic

692. Basipterygoid, ossified basitrabecular process [modified from [11]]:

0. present

1. absent

693. Palate, basipterygoid-pterygoid sesamoid [11]

0. absent

1. present

694. Vidian canal formed by the basisphenoid enclosing the internal carotid artery and the base of the palatine artery as they pass over the basipterygoid process [11]:

0. absent

1. present

695. Basioccipital, position of the basal tubera [11]:

0. posterolaterally placed with the apex on the lateral edge of the basioccipital behind the base of the prootic-otooccipital suture

1. anteromedially placed, with the apex at the lateral juncture of the sphenoid and basioccipital, anterior and medial to the prootic-otooccipital suture

696. Opisthotics, midline contact excluding the basioccipital from the foramen magnum on the occipital condyle [modified from [11]]:

0. absent

1. present

697. Basioccipital, contribution to the medial aperture of the recessus scala tympani [modified from [11]]:

0. present

1. absent

698. Cranial nerve IX, passes through medial aperture of the rescesussus scala tympani [11]:

0. present

1. absent, exits dorsal to the medial aperture

699. Cranial nerve IX, passes laterally through the lateral aperture of the rescesussus scala tympani [11]:

0. absent, passes through the foramen magnum

1. present

2. absent, passes through vagus/jugular foramen

700. Otooccipital, hypoglossal foramen location [modified from [11]]:

0. at approximately the same anteroposterior level as the vagus foramen

1. extending posterior to the vagus foramen

701. Otooccipital, hypoglossal foramina confluent with the vagus foramen [modified from [11]]:

0. none

1. one

2. two

3. three

702. Otooccipital, orientation of the perilymphatic foramen [11]:

0. ventral

1. medial

2. lateral

3. posterior

703. Otooccipital, fusion [11]:

0. incomplete

1. complete--no remnant of the opisthotic-exoccipital suture in adults

704. Braincase, metotic fissure [11]:

0. open, undivided

1. metotic fissure subdivided by contact of the basal plate and otic capsule

705. Dentary, anterodorsal edge of the dental parapet tip [11]:

0. straight

1. "tipped down"

706. Dentary, size of the posteriormost mental foramen [11]:

0. similar to the others

1. enlarged relative to the others

707. Surangular, articular surface receiving the dentary [modified from [11]]:

0. flat articular surface

1. medially depressed surface or "groove"

2. deep, v-shaped, laterally-facing recess

708. Splenial, dorsal attachment to the dentary dorsal to Meckel's canal [modified from [11]]:

0. present for most of the length of the splenial

1. present only posterodorsally, posterior to the level of the anterior inferior alveolar foramen

709. Splenial, position of the anterior inferior alveolar foramen relative to the anterior mylohyoid foramen [11]:

0. aiaf anterior to the level of the anterior mylohyoid foramen

1. aiaf overlaps the level of the anterior mylohyoid foramen

710. Angular, posterior extent [modified from [11]]:

0. reaches the level of the mandibular condyle or the anterior buttress for the condyle

1. lies completely anterior to the level of the mandibular condyle and its anterior buttress

711. Angular, medial exposure [modified from [11]]:

0. "broad"; dorsal and ventral margins of the angular visible in medial view

1. "reduced"; angular primarily wrapping around the ventral mandibular margin with little or no exposure of the ventral suture

2. "narrow"; very little of the angular exposed in medial view such that little more than the dorsal suture is visible

712. Mandible, position of the posterior mylohyoid foramen [modified from [11]]:

0. state (0] omitted; coded as (-]

1. medial

2. ventral

3. lateral

713. Surangular, coronoid eminence [modified from [11]]:

0. present

1. absent

714. Coronoid, anteromedial process fits into a sulcus beneath the tooth-bearing border of the dentary [11]:

0. absent

1. present

2. present, with a process that wraps around the ventral margin of the dentary tooth-bearing border at the apex, posteriorly

715. Coronoid-surangular articulation [11]:

0. coronoid restricted to the medial aspect of the mandible

1. coronoid extends onto the dorsal surface of the surangular

2. coronoid reaches over the dorsal margin of the mandible to reach the lateral surangular face

716. Surangular, overlap with the dentary lateral to the intramandibular septum [modified from [11]]:

0. absent

1. present

2. present with elongate overlap forming a C-shaped ventral, lateral, and dorsal margins of the alveolar branch of the alveolar nerve

717. Surangular, posterior surangular foramen [11]:

0. present

1. absent

718. Mandible, medial wall of the adductor fossa [modified from [11]]:

0. distinct medial wall present

1. distinct medial wall subequal in height to the lateral wall

2. faint medial ridge

3. medial wall extends more dorsally than the lateral wall

719. Surangular, lateral adductor fossa on the external mandibular surface [11]:

0. fossa shallow and does not extend halfway down the lateral surangular surface

1. fossa distinct and deep, extending more than halfway down the lateral surangular surface

720. Prearticular, broad surangular contact anterior to the adductor fossa on the medial surface of the mandible [11]:

0. absent

1. present, length of contact more than one-quarter the length of the adductor fossa

721. Prearticular, dorsoventral orientation of the retroarticular process [11]:

0. oriented posteriorly

1. significantly downturned compared to the main mandibular axis (406:1]

2. upturned in lateral view

722. Prearticular, posterior emargination of the retroarticular process [11]:

0. absent

1. present

723. Dentition, length of the middle maxillary teeth compared with the anterior maxillary teeth [modified from [11]]:

0. similar in size

1. shorter

2. longer

724. Dentition, length of the middle maxillary teeth compared with the posterior maxillary teeth [modified from [11]]:

0. similar

1. shorter

2. longer

725. Dentition, form of the anterior marginal teeth [11]:

0. vertical

1. recurved

726. Dentition, form of the posterior marginal teeth [11]:

0. vertical

1. recurved

727. Dentition, anteroposterior fusion of teeth [11]:

0. absent

1. present

728. Dentition, position of the replacement teeth [11]:

0. lingual

1. posterolingual

2. inferior to the functional teeth

729. Dentition, orientation of replacement teeth [11]:

0. vertical

1. erupt horizontally and then rotate through 90 degrees about the base to come to the functional position

730. Dentition, tooth replacement [11]:

0. present

1. absent

731. Dentition, resorption pits [11]:

0. present

1. absent

732. Dentition, position of resorption pits [11]:

0. at tooth bases

1. on bony tooth pedicel

733. Dentition, relative size of posterior pterygoid teeth compared to palatal teeth anterior to that point [11]:

0. constant size

1. smaller posteriorly

734. Dentition, maxillary teeth with swollen shafts that expand distal to the parapet [11]:

0. absent

1. present

735. Basihyal, position relative to the braincase when the mouth is closed [11]:

0. anterior to braincase

1. ventral to the braincase

2. posterior to the braincase

736. Hyoid, length of lingual process [modified from [11]]:

0. less than 20 percent the mandibular length

1. more than 20 percent of the mandibular length

2. more than 40 percent of the mandibular length

3. more than 60 percent of the mandibular length

737. Hyoid, length of the first epibranchial [11]:

0. shorter than first ceratobranchial

1. subequal to or longer than first ceratobranchial

738. Hyoid, dorsolateral angulation of the first ceratobranchial when the tongue is retracted [modified from [11]]:

0. no dorsolateral angulation

1. weak dorsolateral angulation (has a distinct bend]

2. strong dorsolateral angulation

3. entire element oriented vertically

739. Hyoid, length of the second ceratobranchial [11]:

0. much shorter than the first ceratobranchial (less than one-half the lenght fo the first ceratobranchial]

1. more than one-half the lenght fo the first ceratobranchial

740. Hyoid cornu [11]:

0. less than the lenght of the epihyal

1. greater than or equal to the length of the epihyal

741. Hyoid, epihyal [11]:

0. simple, without flanges

1. proximal flattening

2. hook-like lateral process

3. lateral ala

4. medial flange

742. Hyoid, lateral flange at the midpoint of the epihyal [11]:

0. absent

1. present

743. Ribs, occurrence of the first rib [11]:

0. on the atlas

1. on the axis

2. on vertebra three

3. on vertebra four

4. on vertebra five

5. on vertebra six

6. on vertebra seven

744. Vertebra, intercentrum length compared to the pedicle length on vertebra two [11]:

0. intercentrum longer than pedicle

1. intercentrum shorter than the pedicle

745. Vertebra, anterior vertebra pedicle [11]:

0. absent

1. present

746. Vertebral pedicle, posterior extent [11]:

0. "in anterior half of vertebral column"

1. "throughout vertebral column"

747. Caudal vertebrae, groove on the dorsal surfaces of the posterior caudal neural spines [11]:

0. absent

1. present

748. Caudal vertebrae, pterapophysis [11]:

0. absent

1. present

749. Caudal vertebra, horizontal blade present on the distal tip of the anterior zygapophysis [11]:

0. absent

1. present

750. Suprascapula, size [11]:

0. subequal in length to the scapula

1. length less than 80 percent the length of the scapula

751. Clavicles, midline contact [11]:

0. absent

1. present

752. Interclavicle, position of the anterior end relative to the clavicles [11]:

0. interclavicle ventral to the clavicles

1. interclavicle dorsal to the clavicles

2. interclavicle abuts the clavicles

3. interclavicle lies posterior to the clavicles

753. Pubis, anteroposterior breadth of the symphysial portion [11]:

0. relatively broad, more than one-third the breadth of the thyroid fenestra

1. relatively narrow, less than one-third the breadth of the thyroid fenestra

754. Hypoischium [modified from [11]]:

0. present

1. absent

755. Hypoischium, shape [modified from [11]]:

0. with distal expansion

1. lacking distal expansion

756. Hypoischium, fenestra [modified from [11]]:

0. absent

1. present

757. Hyperischium [11]:

0. present

1. absent

758. Hyperischium, fenestra [11]:

0. absent

1. present

759. Ulnar patella [11]:

0. present

1. absent

760. Ulna, olecranon process [modified from [11]]:

0. prominent, with acute angle formed between articular and posterior borders

1. not prominent; without a strongly developed process including an acute, proximally oriented, process

761. Manus, "enlarged distal epiphysis that is nearly hemispherical in profile and fits into a concomitantly enlarged depression on the ulnare" [11]:

0. absent

1. present

762. Radius, styloid process on posterolateral surface of the distal epiphysis [11]:

0. absent

1. present

763. Carpus, intermedium size [11]:

0. large, similar in size to radiale or ulnare

1. small, much smaller than the radiale or ulnare

764. Carpus, spherically-concave cotyle formed by the radiale, ulnare, and pisiform [11]:

0. absent

1. present

765. Carpus, lateral centrale contact with the second distal carpal [11]:

0. absent

1. present

766. Carpus, medial centrale contact with the first metacarpal [11]:

0. absent

1. present

767. Palmar sesamoid [11]:

0. absent

1. present

768. Manus, length of metacarpals II-IV relative to their associated proximal phalanges [11]:

0. metacarpals longer

1. proximal phalanges longer

769. Metacarpals, distal sesamoids ventrally [11]:

0. absent

1. present

770. Manus, opposing digits [11]:

0. absent

1. present

771. Manus, relative dimensions of the penultimate and antepenultimate phalanges [11]:

0. penultimate phalanges subequal to or shorter than the antepenultimate phalanges

1. penultimate phalanges longer than the antepenultimate phalanges

772. Manus, sesamoid associated with the dorsal side of the distal head of the penultimate phalanges [11]:

0. present

1. absent

773. Femur, dorsoventral curvature [11]:

0. present

1. absent

774. Femur, distinct internal trochanter [11]:

0. present

1. absent

775. Tibial patella [11]:

0. present

1. absent

776. Tibial lunula [11]:

0. present

1. absent

777. Fibular lunula [11]:

0. present

1. absent

778. Knee, dorsal tibiofemoral lunula [modified from [11]]:

0. present

1. absent

779. Knee, ventral tibiofemoral lunula [modified from [11]]:

0. present

1. absent

780. Knee, fusion of dorsal and ventral tibiofemoral lunulae [modified from [11]]:

0. absent

1. present

781. Tibia, distal notch receiving a peg of the astragalocalcaneum [11]:

0. absent

1. present

782. Fibula, extent of contact between the distal fibular end with the astragalus-astragalocalcaneum [11]:

0. less than one half the distal fibular end contacts the tarsus

1. most of the distal fibula contacts the tarsus

783. Tibia, proximity of the distal end to the distal end of the fibula [11]:

0. separated by a gap of more than one-third the breadth of either bone

1. in close proximity such that the separation is less than one-third the distal width of either bone

784. Astragalocalcaenum, socket receiving a convex, semi-hemispherical, condyle formed by the distal tarsal [11]:

0. absent

1. present

785. Tarsus, third distal tarsal [11]:

0. present

1. absent

786. Tarsus, second distal tarsal [11]:

0. present

1. absent

787. Pes, tarsometatarsal sesamoid I [11]:

0. present

1. absent

788. Metatarsals II-IV, length relative to their respective proximal phalanges [11]:

0. longer

1. shorter

789. Metatarsal V, hooking [11]:

0. present

1. absent

790. Metatarsals, sesamoids ventral to the distal ends [11]:

0. absent

1. present

791. Pes, opposing digits [11]:

0. absent

1. present

792. Pes, sesamoids dorsal to the distal heads of the penultimate phalanges [11]:

0. present

1. absent

793. Palpebral, osteoderm below the supraorbital scale [11]:

0. absent

1. present

794. Orbit, supraorbital scale osteoderms [modified from [11]]:

0. absent

1. present

795. Orbit, compound supraorbital scale osteoderms [modified from [11]]:

0. absent, osteoderms single

1. present, supraorbital osteoderms compound

796. Integument, osteoderms in caudal scales [11]:

0. absent

1. present

797. Integument, mineralized cranial scale hinges [11]:

0. absent

1. present

798. Scleral ossicles, shape [11]:

0. complex and irregularly shaped with distally expanded crests

1. square/regular in shape

799. Ears, statolithic masses [11]:

0. absent

1. present

800. Head, calcified endolymph [modified from [11]]:

0. absent

1. present

801. Hemipenis, morphology of the mineralized parts [11]:

0. absent

1. present, comblike

2. sleevelike

3. spinelike

802. Myology, attachment rectus abdominus to the belly skin [11, 59]:

0. absent

1. present

803. Egg-laying [11]:

0. present

1. absent

**References**

1. Barrows S, Smith HM. The skeleton of the lizard *Xenosaurus grandis* (Gray). Univ Kans Science Bull 1947; 31: 227-281.

2. Rieppel O. The phylogeny of anguinimorph lizards. Basel: Naturforschenden Gesellshaft; 1980.

3. Haas G. On the trigeminus muscles of the lizards *Xenosaurus grandis* and *Shinisaurus crocodilurus*. Amer Mus Novit 1960; 2017: 1-54.

4. Bhullar B-A. The enigmatic fossils *Exostinus* and *Restes*: resolving the stem and the crown of *Xenosaurus*, the knob-scaled lizards. J Vert Paleontol 2007; 27 (suppl. 3): 48A.

5. Bhullar B-AS. The power and utility of morphological characters in systematics: a fully resolved phylogeny of *Xenosaurus* and its fossil relatives (Squamata: Anguimorpha). Bull of the Mus Comp Zool 2011; 160: 65-181.

6. Gao K-Q, Hou L. Systematics and taxonomic diversity of squamates from the Upper Cretaceous Djadochta Formation, Bayan Manahu, Gobi Desert, People's Republic of China. Can J Earth Sci 1996; 33: 578-598.

7. Gao K-Q, Norell MA. Taxonomic composition and systematics of Late Cretaceous lizard assemblages from Ukhaa Tolgod and adjacent localities, Mongolian Gobi Desert. Bull Am Mus Nat Hist 2000; 249: 1-118.

8. Conrad JL, Norell MA. A well-preserved iguanian (Squamata: Reptilia) from the Cretaceous of the Gobi and identification of a new clade of Iguania. Amer Mus Novit 2007; 3584: 1-47.

9. The Deep Scaly Project. *Agama agama*. 2008; Available: http://digimorph.org/specimens/Agama_agama.

10. The Deep Scaly Project. *Brookesia brygooi*. 2009; Available: http://digimorph.org/specimens/Brookesia_brygooi.

11. Gauthier JA, Kearney M, Maisano JA, Rieppel O. Behlke DB. Assembling the squamate tree of life: perspectives from the phenotype and the fossil record. Bull of the Peabody Mus Nat Hist 2012; 53: 3-308.

12. Rieppel O. The phylogenetic relationships within the Chamaeleonidae, with comments on some aspects of cladistic analysis. Zool J Linn Soc 1987; 89: 41-62.

13. Rieppel O, Crumly C. Paedomorphosis and skull structure in Malagasy chamaeleons (Reptilia: Chamaeleoninae). J Zool 1997; 243: 351-380.

14. Maisano JA, The Deep Scaly Project. *Physignathus cocincinus*. 2003; Available: http://digimorph.org/specimens/Physignathus_cocincinus.

15. The Deep Scaly Project. *Uromastyx aegyptius*. 2008; Available: http://digimorph.org/specimens/Uromastyx_aegyptius.

16. Estes R. Sauria terrestria, Amphisbaenia. New York: Gustav Fischer Verlag; 1983.

17. Gilmore CW. Fossil lizards of North America. Mem Natl Acad Sci 1928; 22: 1-197.

18. Conrad JL, Rieppel O, Grande L. An Eocene iguanian (Squamata: Reptilia) from Wyoming, U.S.A. J Paleontology 2007; 81: 1375-1383.

19. The Deep Scaly Project. *Anolis carolinensis*. 2006; Available: http://digimorph.org/specimens/Anolis_carolinensis.

20. Scheltinga DM, Jamieson BGM, Espinoza RE, Orrell KS. Descriptions of the mature spermatozoa of the lizards *Crotaphytus bicintores*, *Gambelia wislizenii* (Crotaphytidae), and *Anolis carolinensis* (Polychrotidae) (Reptilia, Squamata, Iguania). J Morphol 2001; 247: 160-171.

21. Polcyn MJ, Rogers JV, II, Kobayashi Y, Jacobs LL. Computed tomography of an *Anolis* lizard in Dominican amber: systematic, taphonomic, biogeographic, and evolutionary implications. Palaeontologia Electronica 2002; 5: 1-13.

22. Polcyn MJ, Rogers JV, II, Kobayashi Y, Jacobs LL. *Anolis* sp. 2002; Available: http://digimorph.org/specimens/Anolis_sp.

23. Rieppel O Green anole in Dominican amber. Nature 1980; 286: 486-487.

24. Norell MA, de Queiroz K. The earliest iguanine lizard (Reptilia: Squamata) and its bearing on iguanine phylogeny. Amer Mus Novit 1991; 2997: 1-16.

25. Lang M. Phylogenetic and biogegraphic patterns of basiliscine iguanians (Reptilia: Squamata: "Iguanidae"). Bonn Zool Monogr 1989; 28: 1-172.

26. Evans SE. The skull of lizards and tuatara. In: C. Gans, A. S. Gaunt and K. Adler, editors. Biology of the Reptilia, volume 20: Morphology H, the skull of Lepidosauria. Ithica, New York: Society for the Study of Amphibians and Reptiles; 2008. pp. 1-344.

27. The Deep Scaly Project. *Brachylophus fasciatus*. 2008; Available: http://digimorph.org/specimens/Brachylophus_fasciatus.

28. The Deep Scaly Project. *Chalarodon madagascariensis*. 2011; Available: http://digimorph.org/specimens/Chalarodon_madagascariensis.

29. McGuire JA. Phylogenetic systematics of crotaphytid lizards (Reptilia: Iguania: Crotaphytidae). Bull of Carnegie Mus Nat Hist 1996; 32: 1-143.

30. Oelrich TM. The anatomy of the head of *Ctenosaura pectinata* (Iguanidae). Misc Publ: Mus Zool, Univ Mich 1956; 94: 1-122.

31. Maisano JA. and The Deep Scaly Project. *Dipsosaurus dorsalis*. 2003; Available: http://digimorph.org/specimens/Dipsosaurus_dorsalis.

32. Smith KT. Eocene lizards of the clade *Geiseltaliellus* from Messel and Geiseltal, Germany, and the early radiation of Iguanidae (Reptilia: Squamata). Bull of the Peabody Mus Nat Hist 2009; 50: 219-306.

33. Rossmann T. Osteologische Beschreibung von *Geiseltaliellus longicaudus* Kuhn, 1944 (Squamata: Iguanoidea) aus dem Mittleren Eozän der Fossillagerstätten Geiseltal und Grube Messel (Deutschland), mit einer Revision der Gattung *Geiseltaliellus*. Palaeontographica Abteilung a Palaeozoologie Stratigraphie 2000; 258: 117-158.

34. Rossmann T. *Geiseltaliellus longicaudus* Kuhn (Lacertilia: Iguanoidea) aus dem Eozän von Mitteleuropa: Neue Erkenntnisse zur Paläobiologie und Paläobiogeographie [*Geiseltaliellus longicaudus* Kuhn (Lacertilia: Iguanoidea) from the Eocene of central Europe: new palaeobiological and palaeobiogeographical results]. Neues Jahrb Geol Palaontol Abh 2001; 221: 1-33.

35. Smith KT. The Middle Eocene *Geiseltaliellus* Geiseltal and Messel Germany, and the evolutio of corytophanid lizards (Squamata: Iguania). J Vert Paleontol 2004; 24 (suppl. 3): 49A-50A.

36. Vieira GHC, Colli GR and Báo SN. The ultrastructure of the spermatozoon of the lizard *Iguana iguana* (Reptilia, Squamata, Iguanidae) and the variability of sperm morphology among iguanian lizards. J Anatomy 2004; 204: 451-464.

37. Gilmore CW. Fossil lizards of Mongolia. Bull Am Mus Nat Hist 1943; 81: 361-384.

38. The Deep Scaly Project. *Leiosaurus catamarcensis*. 2010; Available: http://digimorph.org/specimens/Leiosaurus_catamarcensis.

39. The Deep Scaly Project. *Oplurus cyclurus*. 2007; Available: http://digimorph.org/specimens/Oplurus_cyclurus.

40. Borsuk-Białynicka M Alifanov VR. First Asiatic 'iguanid' lizards in the Late Cretaceous of Mongolia. Acta Palaeontol Pol 1991; 36: 325-342.

41. The Deep Scaly Project. *Polychrus marmoratus*. 2008; Available: http://digimorph.org/specimens/Polychrus_marmoratus.

42. de Queiroz K. Phylogenetic systematics of iguanine lizards: a comparative osteological study. Univ Calif Publ Zool 1987; 118: 1-203.

43. Hollingsworth BD. The systematics of chuckwallas (*Sauromalus*) with a phylogenetic analysis of other iguanid lizards. Herpetological Monographs 1998; 12: 38-191.

44. Smith KT. A new lizard assemblage from the earliest Eocene (zone Wa0) of the Bighorn Basin, Wyoming, USA: biogeography during the warmest intervale of the Cenozoic. J Syst Palaeontol 2009; 7: 299-358.

45. The Deep Scaly Project. *Urostrophus vautieri*. 2010; Available: http://digimorph.org/specimens/Urostrophus_vautieri.

46. The Deep Scaly Project. *Uta stansburiana*. 2006; Available: http://digimorph.org/specimens/Uta_stansburiana.

47. Scheltinga DM, Jamieson BGM, Trauth SE and McAllister CT. Morphology of the spermatozoa of the iguanian lizards *Uta stansburiana* and *Urosaurus ornatus* (Squamata, Phrynosomatidae). J Submic Cytol Pathol 2000; 32: 261-271.

48. Alifanov VR. New priscagamids (Lacertilia) from the Upper Cretaceous of Mongolia and their systematic postion among Iguania. Paleontol J 1989; 1989: 68-80.

49. Alifanov VR. Lizard families Priscagamidae and Hoplocercidae (Sauria, Iguania): phylogenetic position and new representatives from the Late Cretaceous of Mongolia. Paleontol J 1996; 1996: 100-118.

50. Alifanov VR. The fossil record of Cretaceous lizards from Mongolia. In: M. J. Benton, M. A. Shishkin, D. M. Unwin and E. N. Kurochkin, editors. The age of dinosaurs in Russia and Mongolia. Cambridge: Cambridge University Press; 2000. pp. 368-389.

51. Conrad JL. Phylogeny and systematics of Squamata (Reptilia) based on morphology. Bull Am Mus Nat Hist 2008; 310: 1-182.

52. Conrad JL, Ast JC, Montanari S and Norell MA. A combined evidence phylogenetic analysis of Anguimorpha (Reptilia: Squamata). Cladistics 2011; 27: 230-277.

53. Conrad JL, Balcarcel AM and Mehling CM. Earliest example of a giant monitor lizard (*Varanus*, Varanidae, Squamata). PLoS One 2012; 7: 1-14.

54. Vieira GHC, Colli GR and Báo SN. Phylogenetic relationships of corytophanid lizards (Iguania, Squamata, Reptilia) based on partitioned and total evidence analyses of sperm morphology, gross morphology, and DNA data. Zool Scr 2005; 34: 604-625.

55. Hallermann J. Zur Morphologie der Ethmoidalregion der Iguania (Squamata): eine vergleichend-anatomische Untersuchung. Bonn Zool Monogr 1994; 35: 1-133.

56. Etheridge R and de Queiroz K. A phylogeny of Iguanidae. In: R. Estes and G. Pregill, editors. Phylogenetic relationships of the lizard families. Stanford: Stanford University Press; 1988. pp. 283-367.

57. Schwenk K. Comparative morphology of the lepidosaur tongue and its relevance to squamate phylogeny. In: R. Estes and G. Pregill, editors. Phylogenetic relationships of the lizard families. Stanford: Stanford University Press; 1988. pp. 569-597.

58. Kluge AG. Cladistic relationships among gekkonid lizards. Copeia 1983; 1983: 465-475.

59. Bhullar B-AS. A reevaluation of the unusual abdominal musculature of squamate reptiles (Reptilia: Squamata). Anat Rec 2009; 292: 1154-1161.
